# Development of a Shapley Additive Explanations (SHAP)-Interpretable Model for Cardiovascular Risk Stratification in Type 2 Diabetes Mellitus: Implications of MDA-Modified Protein Adducts and Autoantibodies

**DOI:** 10.1021/acsomega.6c05628

**Published:** 2026-08-13

**Authors:** Chien-Yi Hsu, Shou-Cheng Lu, Liang-Wei Lin, Chu-Heng Yen, Hung-Tse Lin, Shao-Chun Lin, Ching-Yu Lin, Chao-Lien Liu

**Affiliations:** † Division of Cardiology, Department of Internal Medicine, School of Medicine, College of Medicine,Taipei Heart Institute, Taipei Medical University, Taipei 11031, Taiwan; ‡ Division of Cardiology and Cardiovascular Research Center, Department of Internal Medicine, Taipei Medical University Hospital, Taipei 11031, Taiwan; § Department of Laboratory Medicine, Shuang Ho Hospital, Taipei Medical University, New Taipei City 23561, Taiwan; ∥ Department of Laboratory Medicine, Taiwan Adventist Hospital, Taipei 10556, Taiwan; ⊥ School of Medical Laboratory Science and Biotechnology, College of Medical Science and Technology, Taipei Medical University, Taipei 11031, Taiwan; # Department of Laboratory Medicine, LinKou Chang Gung Memorial Hospital, Taoyuan 33305, Taiwan; ∇ Department of Medical Biotechnology and Laboratory Science, College of Medicine, Chang Gung University, Taoyuan 33302, Taiwan; ○ Department of Pharmacy, Chia Nan University of Pharmacy & Science, Tainan City 717301, Taiwan; ◆ Ph.D. Program in Medical Biotechnology, College of Medical Science and Technology, Taipei Medical University, Taipei 11031, Taiwan

## Abstract

Coronary artery disease (CAD) is the leading cause of mortality in type 2 diabetes mellitus (T2DM). This study explores the two-hit oxidative–immune hypothesis by evaluating malondialdehyde (MDA)-modified protein (MDA-protein) adducts and their corresponding autoantibodies for precise and fair CAD severity stratification and classification. Novel MDA-peptide epitopes (apolipoprotein B-100 (ApoB-100), fibronectin (FINC), and complement C4A/C4B (C4A/C4B)) were identified via proteomics, and plasma levels of MDA, adducts, and autoantibodies were measured in 165 Taiwanese T2DM patients. Multivariate logistic regression estimated odds ratios (ORs) per 1-SD increase. Machine learning with Shapley additive explanations (SHAP)-interpretable analysis and nested cross validation distinguished obstructive from nonobstructive CAD. Fairness was assessed across age and sex subgroups. MDA (OR 3.148) and MDA-protein adducts (OR 2.090) were independent markers associated with advanced CAD severity, while IgG anti-ApoB-100^1662–1683^ MDA (OR 0.481) was independently associated with a lower risk. In differentiating obstructive CAD in T2DM patients, random forest achieved a pooled AUC of 0.958, a Brier score of 0.089, and a net benefit of 0.433 at the 20% threshold, outperforming conventional markers. SHAP analysis highlighted IgG anti-C4A/B^167–187^ as the dominant positive contributor within the machine learning framework, suggesting a possible proinflammatory signature, underscoring nonlinear push–pull dynamics between oxidative–immune signatures. As an exploratory finding, fairness analysis demonstrated consistent performance across sexes and a notable benefit in older patients, while revealing age-related imbalance in calibration and decision utility. Integrating MDA-related immune signatures into interpretable, fairness-aware categorical boosting and logistic regression models provides a robust, noninvasive framework for CAD severity stratification in T2DM, clarifying immunometabolic interactions and supporting equitable clinical decision-making.

## Graphical abstract

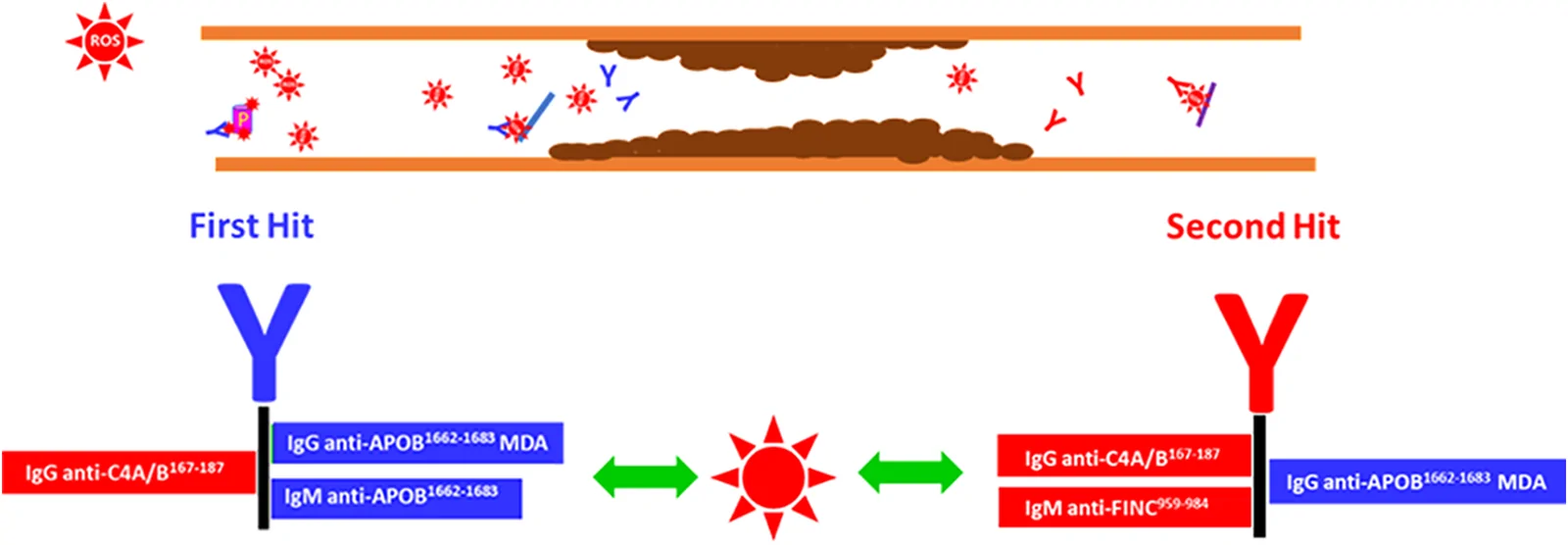

## 1 Introduction

In type 2 diabetes mellitus (T2DM), the coexistence of coronary artery disease (CAD) markedly increases cardiovascular risk and mortality.^1^ Globally, about 32.2% of individuals with type 2 diabetes (T2DM) have cardiovascular disease (CVD).^2^ The global diabetes population is expected to reach 853 million by 2050.^3^ In Taiwan, a retrospective cohort reported a mean CAD incidence of 16.80 per 1000 person-years among T2DM participants, independent of hypertensive complications.^4^ T2DM and CAD share several risk factors, such as obesity, hypertension, dyslipidemia, and sedentary lifestyle, which contribute to endothelial injury and inflammation.^5^ However, standard diagnostic methods are not sensitive enough to detect early immune alterations.

Growing evidence indicates that CAD progression in T2DM is shaped by a complex immunometabolic profile characterized by an oxidative–immune signature. Persistent oxidative stress, clinically reflected by elevated malondialdehyde (MDA), serves as a pivotal contributor to vascular inflammation, accelerating plaque formation and maladaptive vascular remodeling.^5,6^ Hyperglycemia promotes excessive reactive oxygen species, resulting in lipid peroxidation and the formation of MDA, a highly reactive aldehyde that modifies proteins and lipoproteins within the vascular microenvironment.^7^ MDA reacts with apolipoprotein B-100 (ApoB-100) and structural proteins to generate oxidation-specific epitopes (OSEs), which function as damage-associated molecular patterns (DAMPs) to trigger innate immune pathways.^8^ A key manifestation is the formation of MDA-acetaldehyde-modified LDL (MAA-LDL), which provokes stronger proinflammatory responses than standard MDA-LDL, driving macrophage activation and vascular wall injury.^9^ Recognition of these epitopes by pattern recognition receptors, including scavenger receptors and complement components, amplifies vascular inflammation, thereby accelerating atherosclerotic plaque formation.^10^ Natural IgM autoantibodies confer early vascular protection, but this effect wanes as stenosis progresses.^11^ This immunopathological shift is further amplified by epitope spreading, a process where chronic inflammation diversifies immune responses from lipoprotein OSEs to extracellular matrix components.^12^ These changes foster immune complex deposition and complement activation, ultimately generating the membrane attack complex (MAC), which drives endothelial damage and inflammatory cell recruitment.^13^ Such a broad immunometabolic breakdown culminates in plaque rupture or erosion, precipitating acute cardiovascular events.

Oxidative stress biomarkers and immune responses interact in complex, often nonlinear ways, making traditional statistics less effective. To better capture these patterns, we applied ensemble learning with four tree-based models, namely, random forest (RF), eXtreme Gradient Boosting (XGBoost), light gradient boosting machine (LightGBM), and categorical boosting (CatBoost), together with logistic regression (LR), which served as the baseline.^14,15^ To reduce overfitting and data leakage in small clinical data sets, we used nested cross validation (nested CV) to enable unbiased performance evaluation.^16^ For clinical transparency, we applied Shapley additive explanations (SHAP) to deconstruct the models’ decision-making process,^17^ quantifying each biomarker’s contribution and visualizing the push–pull dynamics of cardiovascular risk, thereby improving stratification in T2DM.^18,19^

In this study, we employed a proteomics-based approach to identify CAD-specific MDA-peptide adducts and to characterize the profiles of MDA, MDA-protein adducts, and their autoantibodies in T2DM patients across varying degrees of coronary stenosis. By combining immunological biomarker profiling with interpretable machine learning, we highlight the clinical relevance of MDA-related biomarkers, elucidate the complex immunometabolic patterns associated with disease progression, and provide a transparent framework to visualize the cardiovascular risk dynamics for improved stratification in T2DM.

## 2 Materials and Methods

### 2.1 Study Population

We analyzed 195 serum samples from the Cardiovascular Center and Department of Laboratory Medicine, Lo-Hsu Medical Foundation, Luodong Poh-Ai Hospital (Yilan, Taiwan), and the Department of Family Medicine and Department of Laboratory Medicine, Shuang-Ho Hospital (New Taipei City, Taiwan). The cohort included 45 T2DM patients with nonobstructive CAD (T2DM/CAD < 70% stenosis; 64.40 ± 9.87 years), 60 T2DM patients with obstructive CAD (T2DM/CAD ≥ 70% stenosis; 64.80 ± 9.87 years), 30 patients with obstructive CAD (CAD ≥ 70% stenosis; 64.97 ± 11.05 years), and 60 age- and sex-matched T2DM controls without CAD (58.30 ± 11.48 years). T2DM patients were diagnosed per American Diabetes Association criteria.^20^ CAD patients underwent quantitative coronary angiography (QCA) by a cardiologist, confirming ≥70% stenosis in at least one major epicardial vessel. Stenosis was quantified as (1–[diseased/reference diameter]) × 100%.^21^ Significant coronary stenosis was defined as ≥70% luminal narrowing in major coronary arteries or ≥50% in the left main artery.^22^

### 2.2 Study Protocols

Figure 1 outlines the study design. From 195 patients, 90 plasma samples (*n* = 30 per group: T2DM, T2DM/CAD ≥ 70% stenosis, CAD ≥ 70% stenosis) were randomly selected to assess protein MDA modification, as well as autoantibody isotypes and levels against bovine serum albumin (BSA), MDA-BSA adducts, and unmodified and MDA-peptide adducts. We identified novel MDA-peptide adducts using trichloroacetic acid (TCA)–acetone precipitation, in-solution digestion, nanoliquid chromatography–tandem mass spectrometry (nano-LC–MS/MS), and PEAKS 7 software (Bioinformatics Solutions, Waterloo, OA, Canada) from pooled samples for each group. Protein MDA modifications were validated by immunoprecipitation and western blotting (IP-WB) from individual and pooled samples. Protein levels were quantified using an enzyme-linked immunosorbent assay (ELISA) of individual samples. Additionally, ELISA was performed in 165 individuals, T2DM (*n* = 60), T2DM/CAD < 70% stenosis (*n* = 45), and T2DM/CAD ≥ 70% stenosis (*n* = 60), to measure MDA, MDA-protein adducts, and autoantibody isotypes against unmodified and MDA-peptide adducts. Independent associations of MDA, MDA-protein adducts, and autoantibodies with disease severity or protection were analyzed between T2DM/CAD ≥ 70% stenosis and T2DM and T2DM/CAD < 70% stenosis. Finally, assay data were analyzed through feature selection, ranking, modeling, interpretation, and fairness assessment. To enhance prediction, we applied an ensemble of LightGBM, CatBoost, XGBoost, Random Forest, and LR, with SHAP analysis for clinical interpretability. CatBoost and LR served as the primary severity stratification model, and fairness evaluation ensured consistent performance across demographic groups.^14,16,17,23^ Sample size estimation and power analysis addressed cohort limitations (Table S1). Plasma was stored at −80 °C. The study protocol was approved by the Taipei Medical University-Joint Institutional Review Board (N201806020 [July 10, 2018] and N202305043 [May 23, 2023]) and by the Institutional Review Board of Cathay General Hospital (CGH-LP106001, [June 15, 2017]). All volunteers provided informed consent before participating.

**Figure 1:**
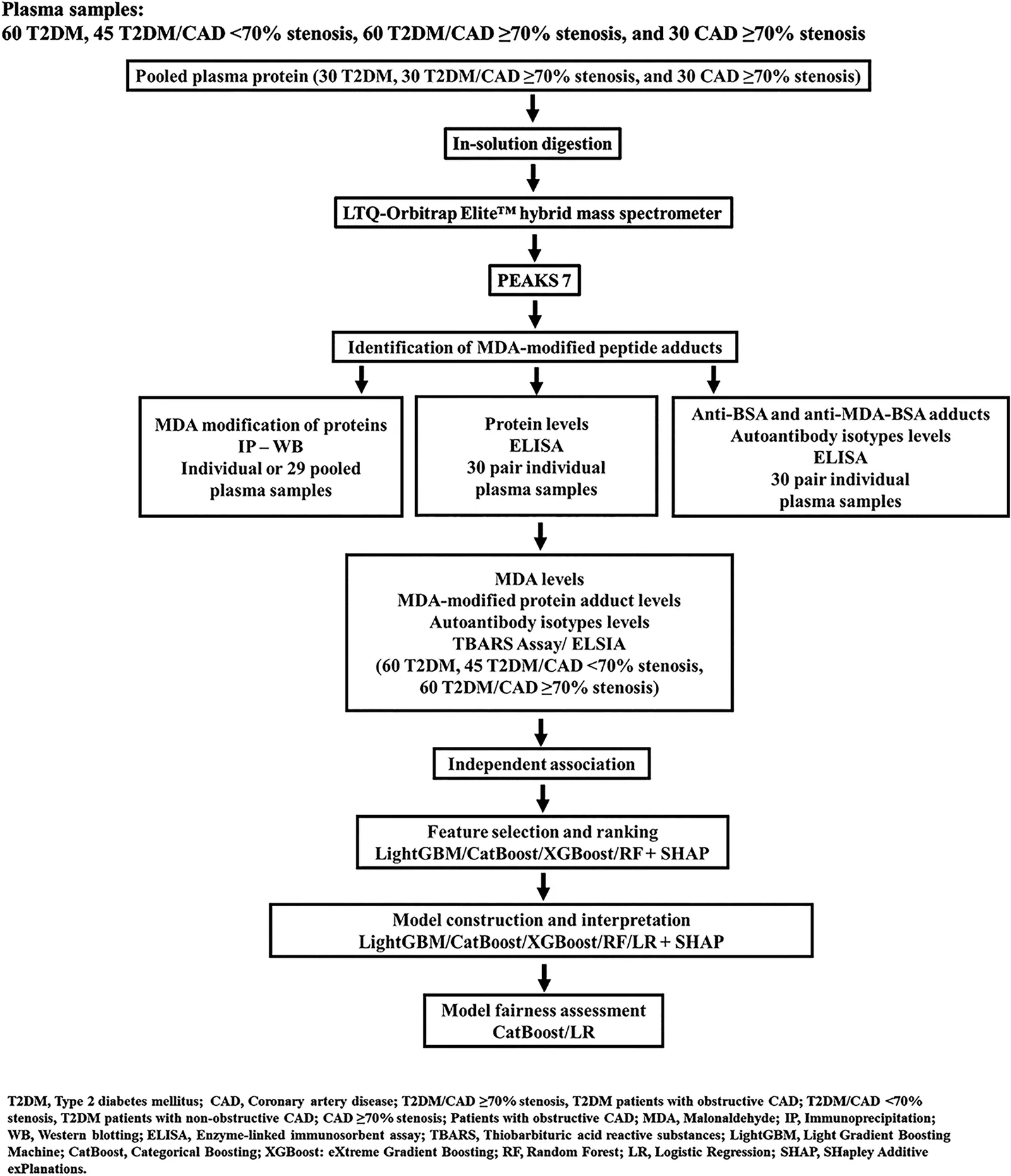
Flowchart.

### 2.3 TCA–Acetone Precipitation, In-Solution Digestion, and Identification of MDA and MAA Modifications via Nano-LC–MS/MS and PEAKS 7 Software

Plasma proteins were isolated using TCA–acetone precipitation (Poltep method) and quantified using the Pierce Coomassie Plus (Bradford) Assay Kit (Thermo Fisher Scientific, Waltham, MA, USA) following manufacturer’s instructions.^24^ Three micrograms of protein underwent in-solution digestion using a modified Glatter protocol.^25^ Tryptic peptides were analyzed on a nanoAcquity Nano-LC-nanoESi-MS/MS system (Waters, Milford, MA, USA) coupled to an LTQ-Orbitrap Elite hybrid mass spectrometer (Thermo Fisher Scientific, Bremen, Germany) with a PicoView nanospray interface. De novo sequencing with the PeaksPTM module of PEAKS 7 software identified novel MDA- and MAA-peptide sequences and protein sites. MS/MS data are available in the ProteomeXchange PRIDE database (PXD046723). Details are provided in the Supporting Information.

### 2.4 Confirmation of MDA-Modified Proteins via IP-WB

IP was conducted using TCA–acetone-derived plasma proteins with antibodies, including goat antihuman ApoB-100 polyclonal antibody (pAb) (Cloud-Clone, Houston, TX, USA), rabbit antihuman fibronectin (FINC) pAb (Boster Biological Technology, Pleasanton, CA, USA), and rabbit antihuman complement C4A/complement C4B (C4A/C4B) monoclonal antibody (mAb) (Cell Signaling Technology, Danvers, MA, USA). MDA-protein modification was confirmed via WB using a rabbit anti-MDA pAb (Cell Biolabs, San Diego, CA, USA). Details are provided in the Supporting Information.

### 2.5 Evaluation of Protein Levels via ELISA

Plasma protein concentrations were measured using ELISA kits according to the manufacturer’s instructions. Kits included ApoB-100 (human apolipoprotein B matched antibody pair kit; Antibodies.com, Cambridge, UK), FINC (human fibronectin ELISA kit EZ; Boster Biological Technology), C4A (human C4A ELISA kit; Elabscience Biotechnology, Houston, TX, USA), and C4B (human C4B ELISA kit; Cusabio Technology, Houston, TX, USA). Details are provided in the Supporting Information.

### 2.6 Quantification of MDA, MDA-Protein Adducts, Autoantibodies Recognizing BSA, MDA-BSA Adducts, and Unmodified and MDA-Peptide Adducts via ELISA

Plasma MDA was measured using a thiobarbituric acid-reactive substances (TBARS) assay.^26^ MDA-protein adducts were assessed by ELISA with minor modifications from Choudhuri et al.^27^ Polypeptides synthesized by Yao-Hong Biotechnology (New Taipei City, Taiwan) included ApoB-100^1662–1683^, FINC^959–984^, C4A^167–187^, and C4B^167–187^ (C4A/C4B^167–187^). Peptides were modified with MDA bis­(dimethyl acetal) (Merck, Darmstadt, Germany).^27^ MDA-peptide adducts were named ApoB-100^1662–1683^ MDA, FINC^959–984^ MDA, and C4A/C4B^167–187^ MDA. Autoantibody isotypes against BSA, MDA-BSA adducts, and unmodified and MDA-peptide adducts were evaluated in duplicate.^27^ Further details are provided in the Supporting Information.

### 2.7 Feature Selection and Ranking

Feature selection employed four tree-based models (LightGBM, CatBoost, XGBoost, and RF) to distinguish T2DM/CAD ≥ 70% stenosis from T2DM/CAD < 70% stenosis and T2DM.^14^ Within the Snap-Cov framework, SHAP values quantified each variable’s contribution, and bootstrap resampling (100 iterations) produced 95% confidence intervals (95% CIs) for SHAP with coefficient of variation (SHAP-Cov), AUC, and Brier score.^28,29^ Features were retained only if SHAP-Cov (95% CI) excluded zero and showed consistent effects across Q1 and Q4.^30^ The final feature was optimized for maximum AUC with a Brier score <0.2, while multicollinearity was controlled by requiring a variance inflation factor (VIF) < 5.^31^ Ranking was based on mean |SHAP value|, balancing discrimination, calibration, and interpretability. Details are provided in the Supporting Information.

### 2.8 Model Construction and Interpretation

We applied five models (RF, LightGBM, CatBoost, XGBoost, and LR) for comparing the T2DM, T2DM/CAD < 70% stenosis, and T2DM/CAD ≥ 70% stenosis groups. Missing values were imputed using multivariate imputation by chained equations (MICE) and standardized; incomplete data were observed only in fasting plasma glucose (Glucose AC, 20.6%), glycated hemoglobin (HbA1c, 3.6%), and total cholesterol (TC, 19.4%), while other features were complete.^16^ Six blood tests (glucose AC, HbA1c, high-density lipoprotein-cholesterol (HDL-c), low-density lipoprotein-cholesterol (LDL-c), TC, triglycerides (TGs)) were reduced to four cardiometabolic indices: TyG index, the HbA1c/HDL-c ratio, Castelli risk index I (CRI I), and Castelli risk index II (CRI II).^32^ To prevent data leakage, we implemented a nested CV framework. The outer loop used repeated stratified *K*-fold CV (five folds, repeated five times), combined with 2000 bootstrap resamples, to evaluate performance metrics and derive 95% CIs as measures of uncertainty. Within this structure, an inner three-fold CV loop was used for feature selection and hyperparameter tuning. Within each inner loop’s training partition, redundant predictors were sequentially filtered using thresholds for Spearman’s rank correlation (ρ) > 0.60, VIF < 10,^31^ and a bootstrap-based SHAP stability assessment (SHAP-Cov < 1.50 across 500 resamples). Sequential forward selection (SFS) was then performed within the same inner loop under strict events-per-variable (EPV) constraints (EPV ≥ 15), yielding highly parsimonious, model-tailored subsets of just 2–3 predictors. To strictly prevent data leakage, these selected feature configurations and their optimized model parameters were subsequently evaluated on the held-out outer-loop test partitions to obtain an unbiased estimate of the framework’s generalization performance.^28,33^ The hyperparameter search spaces used to fine-tune the five machine learning models are detailed in Table S2. Further details are provided in the Supporting Information.

### 2.9 Model Fairness

A calibrated CatBoost model was benchmarked against an uncalibrated LR baseline in a nested CV setting. The outer loop used a fivefold repeated stratified CV (repeated five times), with a three-fold grid search in the inner loop.^16^ OOF predictions were aggregated into a cohort-level risk profile. Performance was evaluated by pooled AUC, Brier score, and NB at 20%, with uncertainty estimated by 1000 bootstrap resamples.^28^ Fairness was examined across sex and age (≥65 vs <65) through subgroup analyses to detect bias and disparities in clinical utility.^23^ The hyperparameter search spaces for both machine learning models are provided in Table S2. Details are provided in the Supporting Information.

### 2.10 Statistical Analysis

Data normality was tested with the Kolmogorov–Smirnov method. For normally distributed variables, comparisons used Student’s *t*-test or one-way ANOVA; for non-normal data, the Mann–Whitney *U*-test, Kruskal–Wallis test, or chi-squared test was applied. Statistical significance was set at *p* < 0.05. In three-group analyses, Scheffé’s posthoc test with Bonferroni correction was used (*p* < 0.0167). Associations among features were examined using Spearman’s *ρ*. Independent associations between plasma biomarkers and CAD severity were assessed by logistic regression, comparing T2DM and T2DM/CAD < 70% stenosis (lower risk) with T2DM/CAD ≥ 70% stenosis (higher risk). Each biomarker was analyzed in three models: model A (unadjusted), model B (adjusted for age and sex), and model C (further adjusted for LDL-c, HbA1c, and PCr). Biomarkers were standardized (z-scores), and covariates were retained in the original scale. Odds ratios per one standard deviation (per 1-SD OR) with 95% CIs and two-sided *p*-values were reported, with multiple testing controlled by Benjamini–Hochberg to yield false discovery rate (FDR)-adjusted *q*-values (*q* < 0.05 significant). Power was calculated using a two-sample *t*-test (two-tailed α = 0.05), with effect size estimated by Cohen’s d from group means and pooled SDs. Model performance was evaluated by mean and pooled AUC, sensitivity, specificity, and F1-score (95% CI). Calibration was optimized using Platt scaling and assessed by the Brier score and calibration slope (95% CI). Clinical utility was examined by decision curve analysis (DCA), focusing on net benefit at 20% threshold probability (NB at 20%).^16^ Fairness was assessed by the overall AUC, Brier score, and NB at 20%. Interpretability was achieved with SHAP analysis, using bar, summary, and dependence plots for global insights and waterfall and force plots for local explanations. Analyses were conducted in Python 3.12 (scikit-learn 1.2 and SHAP 0.42) and SPSS Statistics 18.0 (IBM, Armonk, NY, USA). Power estimation was performed using Python 3.12 and statsmodels 0.14.0. Further details are provided in the Supporting Information.

## 3 Results

### 3.1 Baseline Characteristics

Patient demographics and clinical characteristics for T2DM, T2DM/CAD, and CAD are summarized in Table 1. IgM/IgG anti-BSA, IgG anti-MDA-BSA adduct, and C4B did not differ among T2DM, T2DM/CAD ≥ 70% stenosis, and CAD ≥ 70% stenosis groups, while age, sex, IgM anti-MDA-BSA adduct, ApoB-100, FINC, and C4A differed significantly. In T2DM, T2DM/CAD < 70% stenosis, and T2DM/CAD ≥ 70% stenosis groups, sex, alcohol consumption, smoking, hypertension, glucose AC, HDL-c, TG, TyG index, and most antibodies were similar. Significant differences appeared in age, end-stage renal disease (ESRD), lipid-lowering agents, HbA1c, TC, LDL-c, plasma creatinine (PCr), HbA1c/HDL-c ratio, CRI I/II, MDA, MDA-protein adducts, and multiple IgM/IgG autoantibodies against ApoB-100, FINC, and C4A/C4B or MDA epitopes.

**Table 1:** Demographic and Clinical Characteristics of Participants Who Provided Plasma Samples: 60 T2DM, 45 T2DM/CAD < 70% Stenosis, 60 T2DM/CAD ≥ 70% Stenosis, and 30 CAD ≥ 70% Stenosis

		T2DM/CAD^b^ stenosis (%)			CAD^b^ stenosis (%)	
characteristic^a^	T2DM^b^	<70	≥70		≥70	*p* ^c^
Samples	*n* = 30	N/A	*n* = 30		*n* = 30	
Age (years)	56.23 ± 11.25	N/A	64.57 ± 9.88^d^ ^k^		64.97 ± 11.05^e^ ^j^	0.001
Sex						
Male, *n* (%)	40 (66.67)	N/A	43 (71.67)		52 (86.67)	0.0318
IgM anti-BSA (OD_450/620_)	0.55 ± 0.06	N/A	0.55 ± 0.06		0.57 ± 0.06	0.960
IgM anti-MDA-BSA adduct (OD_450/620_)	2.32 ± 0.12	N/A	2.07 ± 0.16		1.88 ± 0.12^e^ ^i^	0.047
IgG anti-BSA (OD_450/620_)	1.45 ± 0.10	N/A	1.85 ± 0.11^d^ ^j^		0.57 ± 0.06	0.055
IgG anti-MDA-BSA adduct (OD_450/620_)	4.53 ± 0.21	N/A	4.81 ± 0.20		4.19 ± 0.22^h^ ^i^	0.125
ApoB-100 (mg/dL)	53.87 ± 32.31	N/A	90.18 ± 39.57^d^ ^k^		100.46 ± 47.15^e^ ^k^	0.0001
FINC (μg/mL)	0.74 ± 0.21	N/A	0.88 ± 0.29^d^ ^j^		0.80 ± 0.18	0.0035
C4A (μg/mL)	1.22 ± 0.02	N/A	1.52 ± 0.39^d^ ^k^		1.36 ± 0.17^e^ ^k^	<0.0001
C4B (μg/mL)	31.06 ± 25.99	N/A	36.50 ± 31.26		24.71 ± 26.37	0.2017
						
Samples	*n* = 60	*n* = 45	*n* = 60		N/A	
Age (years)	58.30 ± 11.48	64.40 ± 9.87	64.80 ± 9.87		N/A	0.0014
Sex						
Male, *n* (%)	40 (66.67)	25 (55.56)	43 (71.67)		N/A	0.2237
Drinker, *n* (%)	N/A	5 (8.33)	11 (18.33)		N/A	0.4134
Used to smoke, *n* (%)	N/A	6 (10.00)	15 (25.00)		N/A	0.2172
Current smoker, *n* (%)	N/A	9 (15.00)	20 (33.33)		N/A	0.1856
ESRD, *n* (%)	N/A	1 (1.67)	15 (25.00)		N/A	0.0009
Hypertension, *n* (%)	N/A	41 (91.11)	4 7 (78.33)		N/A	0.1088
Lipid-lowering agents, *n* (%)	N/A	10 (22.22)	26 (43.33)		N/A	0.0371
Blood tests						
Glucose AC (mg/dL)	159.6 ± 53.8	147.19 ± 69.04	144.62 ± 56.71		N/A	0.293
HbA1c (%)	7.84 ± 1.61	6.98 ± 1.33^f^ ^k^	7.54 ± 1.60^g^ ^i^		N/A	0.006
HDL-c (mg/dL)	43.47 ± 7.26	44.55 ± 6.25	43.36 ± 7.39		N/A	0.34
LDL-c (mg/dL)	105.26 ± 37.35	76.39 ± 32.38^f^ ^j^	71.09 ± 36.02^d^ ^k^		N/A	<0.0001
TC (mg/dL)	189.17 ± 41.93	148.53 ± 36.26^f^ ^k^	142.41 ± 43.96^d^ ^k^		N/A	<0.0001
TG (mg/dL)	202.17 ± 199.55	150.61 ± 132.7	176.24 ± 109.16		N/A	0.062
PCr (mg/dL)	0.86 ± 0.22	0.96 ± 0.41	1.31 ± 1.01^d^ ^k^		N/A	0.001
Cardiometabolic indices						
TyG index	9.37 ± 0.84	9.10 ± 0.63	9.30 ± 0.48		N/A	0.094
HbA1c/HDL-c ratio (%/mg/dL)	0.18 ± 0.08	0.15 ± 0.06^f^ ^j^	0.16 ± 0.06^g^ ^i^		N/A	0.006
CRI I	4.53 ± 1.57	3.52 ± 1.15^f^ ^k^	3.52 ± 0.99^d^ ^k^		N/A	<0.0001
CRI II	2.54 ± 1.09	1.75 ± 0.69^f^ ^k^	1.70 ± 0.95^d^ ^k^		N/A	<0.0001
MDA (nmol/L)	253.57 ± 156.52	263.78 ± 119.92	384.34 ± 279.00^d^ ^g^ ^i^		N/A	<0.0001
MDA-protein adduct (ng/mL)	181.75 ± 44.15	234.00 ± 42.23^f^ ^k^	260.29 ± 104.02^d^ ^k^		N/A	<0.0001
IgM anti-ApoB-100^1662–1683^ (ng/mL)	640.04 ± 442.82	614.89 ± 828.33^f^ ^i^	569.48 ± 688.46^d^ ^j^		N/A	0.011
IgM anti-ApoB-100^1662–1683^ MDA (ng/mL)	2750.9 ± 2303.58	2164.68 ± 1760.36	1911.85 ± 1677.45^d^ ^k^		N/A	0.034
IgG anti-ApoB-100^1662–1683^ (ng/mL)	383.85 ± 175.59	394.6 ± 80.63^f^ ^i^	403.85 ± 93.23^d^ ^j^		N/A	0.012
IgG anti-ApoB-100^1662–1683^ MDA (ng/mL)	2081.75 ± 640.72	1432.34 ± 542.59^f^ ^k^	1388.01 ± 585.47^d^ ^k^		N/A	<0.0001
IgM anti-FINC^959–984^ (ng/mL)	660.94 ± 284.29	912.55 ± 880.32	738.51 ± 446.75		N/A	0.576
IgM anti-FINC^959–984^ MDA (ng/mL)	3500.23 ± 3173.18	3186.26 ± 2832.1	2893.42 ± 5033.67^d^ ^i^		N/A	0.081
IgG anti-FINC^959–984^ (ng/mL)	286.66 ± 58.15	317.69 ± 69.38^f^ ^j^	294.54 ± 54.71		N/A	0.006
IgG anti-FINC^959–984^ MDA (ng/mL)	2536.11 ± 1324.26	2254.24 ± 1237.54	1874.47 ± 931.55^d^ ^i^		N/A	0.051
IgM anti-C4A/C4B^167–187^(ng/mL)	407.31 ± 304.79	554.64 ± 705.16	467.22 ± 393.25		N/A	0.832
IgM anti-C4A/C4B^167–187^ MDA (ng/mL)	1880.89 ± 1608.35	1645.46 ± 1907.98	1372.23 ± 1551.86^d^ ^k^		N/A	0.023
IgG anti-C4A/C4B^167–187^ (ng/mL)	296.08 ± 38.85	386.63 ± 64.62^f^ ^k^	371.32 ± 80.42^d^ ^k^		N/A	<0.0001
IgG anti-C4A/C4B^167–187^ MDA (ng/mL)	1432.67 ± 728.09	1035.93 ± 347.48^f^ ^k^	1022.06 ± 415.71^d^ ^j^		N/A	0.0033

a Glucose AC (hexokinase, G6PDH; Abbott Alinity c; cutoff <100 mg/dL), HbA1_C_ (capillary electrophoresis; Sebia capillary 3 tera; cutoff <6.0%), total cholesterol (enzymatic, cholesterol oxidase, peroxidase; Abbott Alinity c; cutoff <200 mg/dL), HDL-c (homogeneous, cholesterol oxidase, peroxidase, cholesterol esterase; Abbott Alinity c; cutoff >40 mg/dL), LDL-c (homogeneous, liquid-selective detergent, cholesterol oxidase, cholesterol esterase; Abbott Alinity c; cutoff <130 mg/dL), TG (glycerol phosphate oxidase; Abbott Alinity c; cutoff <150 mg/dL), and PCr (colorimetry, kinetic alkaline pirate; Abbott Alinity c; cutoff <1.2 mg/dL). ApoB-100, apolipoprotein B-100; FINC, fibronectin; C4A, complement C4A; C4B, complement C4B; MDA, malondialdehyde; MDA-protein adduct, malondialdehyde-modified protein adduct; Ig, immunoglobulin.

b T2DM, type 2 diabetes mellitus; CAD, coronary artery disease; T2DM/CAD ≥ 70% stenosis, T2DM patients with obstructive CAD; T2DM/CAD < 70% stenosis, T2DM patients with nonobstructive CAD; CAD ≥ 70% stenosis, patients with obstructive CAD; ESRD, end-stage renal disease; HbA1c, glycated hemoglobin; TC, total cholesterol; HDL-c, high-density lipoprotein-cholesterol; LDL-c, low-density lipoprotein-cholesterol; TG, triglyceride; PCr, plasma creatinine; TyG index, triglyceride–glucose index, calculated as the natural logarithm (ln) of [fasting triglycerides (mg/dL) × fasting glucose (mg/dL)/2]; HbA1c/HDL-c ratio, ratio of glycated hemoglobin (%) to high-density lipoprotein-cholesterol (mg/dL); CRI I, Castelli risk index I, calculated by dividing total cholesterol by high-density lipoprotein-cholesterol (TC/HDL-c); CRI II, Castelli risk index II, determined as the ratio of low-density lipoprotein-cholesterol to high-density lipoprotein-cholesterol (LDL-c/HDL-c); N/A, not available.

c *p* < 0.05 was used to determine statistical significance in comparisons among the three groups. Statistical significance among the two groups was adjusted to *p* < 0.0167 using Scheffé’s method with Bonferroni correction.

d T2DM vs T2DM/CAD ≥ 70% stenosis.

e T2DM vs CAD ≥ 70% stenosis.

f T2DM vs T2DM/CAD < 70% Stenosis.

g T2DM/CAD < 70% stenosis vs T2DM/CAD ≥70% stenosis.

h T2DM/CAD ≥ 70% stenosis vs CAD ≥ 70% stenosis.

i *p* < 0.05.

j *p* < 0.01.

k *p* < 0.001.

### 3.2 Identification and Confirmation of Novel MDA Modifications of Proteins

Novel MDA-peptide adducts were detected in T2DM, T2DM/CAD ≥ 70% stenosis, and CAD ≥ 70% stenosis patients (Table 2). No MS/MS signals were found for MAA-peptide adducts. Sequence coverage averaged 53.0% for ApoB-100, 63.2% for FINC, and 32.2% for C4A/C4B (Table S3).

**Table 2:** Identification of MDA-Peptide Adducts in Patients with T2DM, T2DM/CAD ≥ 70% Stenosis, and CAD ≥ 70% Stenosis

protein^a^	modified peptide^b^		position^c^	Obs.^d^	Calc.^e^	dM^f^	dM^g^	sequence found with modification^a^		
								T2DM	T2DM/CAD	CAD
									≥70% stenosis	
ApoB-100		C(+54.01)SLLVLENELNAELGLSGASMK	1662–1683	782.395	782.396	–0.00019	0.2	–	+	+
ApoB-100		YRITEN(+54.01)DIQIALDDAK	2149–2164	644.662	644.662	–0.00019	0.3	+	–	–
FINC		N(+54.01)TFAEVTGLSPGVTYYFKVFAVSHGR	959–984	967.822	967.824	0.00164	–1.7	–	+	+
FINC		GPGPGLLLLAVQCLGTAVPSTGASKSK(+54.01)	4–30	859.474	859.475	0.00057	–0.7	+	–	–
C4A		M(+54.01)R(+54.01)PSTDTITVMVENSHGLRVR	167–187	836.422	836.421	–0.00065	0.8	–	+	+
C4A		LVNGQSH(+54.01)ISLSK	305–316	446.245	446.245	0.00087	–2.0	+	–	–
C4B		M(+54.01)R(+54.01)PSTDTITVMVENSHGLRVR	167–187	836.422	836.421	–0.00065	0.8	–	+	+
C4B		LVNGQSH(+54.01)ISLSK	305–316	446.245	446.245	0.000876	–2.0	+	–	–

a ApoB-100, apolipoprotein B-100; FINC, fibronectin; C4A, complement C4A; C4B, complement C4B; T2DM, type 2 diabetes mellitus; CAD, coronary artery disease; T2DM/CAD ≥ 70% stenosis, T2DM patients with obstructive CAD; T2DM/CAD < 70% stenosis, T2DM patients with nonobstructive CAD; CAD ≥ 70% stenosis; CAD ≥ 70% stenosis, patients with obstructive CAD; MDA-peptide adducts, malondialdehyde-modified peptide adducts.

b Site of the modified peptide.

c Amino acid positions of the first and last residues in the accession number.

d Obs., observed *m*/*z* of the modified peptides.

e Calcd., calculated *m*/*z* of the modified peptides.

f dM (observed–calculated): mass accuracy.

g Modified peptide mass accuracies (ppm).

Figure S1 shows MS/MS spectra of MDA-peptide adducts analyzed by PEAKS 7. Four peptides were identified in ApoB-100^1662–1683^, FINC^959–984^, C4A^167–187^, and C4B^167–187^ from T2DM/CAD ≥ 70% and CAD ≥ 70% patients, each with MDA modifications causing a 54.010567 Da mass shift. Four additional peptides, ApoB-100^2149–2164^, FINC,^4–30^ C4A^305–316^, and C4B^305–316^, bearing similar modifications were found in T2DM patients. Plasma protein MDA modifications were confirmed by IP-WB in 29 pooled and single-paired samples, with bands at approximately 560, 140, and 210 kDa corresponding to ApoB-100, FINC, and C4A/C4B. A duplicate gel was stained with CBB for loading control (Figure S2).

### 3.3 Determination of Protein Levels in Plasma

In Table 1, ELISA analysis of four plasma proteins showed that ApoB-100 (*p* = 0.0001), FINC (*p* = 0.0035), and C4A (*p* < 0.0001) were significantly elevated in patients with T2DM/CAD ≥ 70% stenosis or CAD ≥ 70% stenosis compared with those with T2DM alone. Specifically, ApoB-100, FINC, and C4A increased by 1.67-fold (*p* < 0.0001), 1.19-fold (*p* = 0.0018), and 1.25-fold (*p* < 0.0001), respectively, in patients with T2DM/CAD ≥ 70% stenosis, while ApoB-100 and C4A increased by 1.86-fold (*p* = 0.0005) and 1.11-fold (*p* = 0.0007), respectively, in patients with CAD ≥ 70% stenosis.

### 3.4 Determination of Plasma MDA, MDA-Protein Adducts, and Autoantibody Isotypes to BSA, MDA-BSA Adducts, and Unmodified and MDA-Peptide Adducts

In Table 1, IgM anti-MDA-BSA adduct levels (*p* = 0.047) were significantly reduced in patients with T2DM/CAD ≥ 70% stenosis or CAD ≥ 70% stenosis compared with those with T2DM. In particular, patients with CAD ≥ 70% stenosis showed a 1.23-fold reduction in IgM anti-MDA-BSA (p = 0.014) compared with T2DM, while patients with T2DM/CAD ≥ 70% stenosis exhibited a nonsignificant 1.12-fold reduction. The IgG anti-MDA-BSA adduct level in patients with T2DM/CAD ≥ 70% stenosis increased 1.06-fold compared with those with T2DM (not significant) but decreased 1.15-fold compared with those with CAD ≥ 70% stenosis (*p* = 0.037). The IgG anti-BSA adduct level in patients with T2DM/CAD ≥ 70% stenosis increased 1.28-fold compared with those with T2DM (*p* = 0.009), while patients with CAD ≥ 70% stenosis showed a 1.14-fold increase without significance. However, IgM anti-BSA adduct levels presented no significant differences among T2DM, T2DM/CAD ≥ 70% stenosis, and CAD ≥ 70% stenosis groups.

Table 1 demonstrates markedly higher MDA (*p* < 0.0001) and MDA-protein adducts (*p* < 0.0001) in T2DM, T2DM/CAD < 70%, and ≥70% stenosis groups. In the T2DM/CAD ≥70% stenosis group, MDA increased 1.52-fold (*p* = 0.0002) and MDA-protein adducts increased 1.44-fold (*p* < 0.0001) compared with the T2DM group; MDA-protein adducts were 1.28-fold higher (*p* < 0.0001) in the T2DM/CAD < 70% stenosis group.

As shown in Table 1, autoantibody profiles differed among T2DM, T2DM/CAD < 70%, and ≥ 70% stenosis groups. IgM anti-ApoB-100^1662–1683^ (*p* = 0.011), IgM anti-ApoB-100^1662–1683^ MDA (*p* = 0.034), IgG anti-ApoB-100^1662–1683^ MDA (*p* < 0.0001) levels were decreased, while IgG anti-ApoB-100^1662–1683^ levels were increased (*p* = 0.012). IgG anti-C4A/C4B^167–187^ levels were elevated (*p* < 0.0001), while IgG anti-C4A/C4B^167–187^ MDA levels were decreased (*p* = 0.0033). In contrast, IgM and IgG titers for anti-FINC^959–984^ and anti-FINC^959–984^ MDA, as well as IgM anti-C4A/C4B^167–187^ and anti-C4A/C4B^167–187^ MDA, showed no significant differences. Compared with patients with T2DM, those with T2DM/CAD ≥ 70% stenosis showed decreased levels of IgM anti-ApoB-100^1662–1683^, IgM anti-ApoB-100^1662–1683^ MDA and IgG anti-ApoB-100^1662–1683^ MDA (1.44-fold, *p* = 0.00092; 1.12-fold, *p* = 0.0049; 1.50-fold, *p* < 0.00001), whereas IgG anti-ApoB-100^1662–1683^ levels were increased (1.05-fold, *p* = 0.0047). IgM and IgG anti-FINC^959–984^ MDA levels were also decreased (1.21-fold, *p* = 0.0269; 1.35-fold, *p* = 0.0137, respectively). For C4A/C4B^167–187^, IgM anti-C4A/C4B^167–187^ MDA levels were lower (1.37-fold, *p* = 0.00075), IgG anti-C4A/C4B^167–187^ levels were higher (1.25-fold, *p* < 0.00001), and IgG anti-C4A/C4B^167–187^ MDA levels were reduced (1.40-fold, *p* = 0.004), with no significant IgM change. Compared with patients with T2DM, those with T2DM/CAD < 70% stenosis showed decreased IgM anti-ApoB-100^1662–1683^ levels (1.04-fold, *p* = 0.023), increased IgG anti-ApoB-100^1662–1683^ levels (1.03-fold, *p* = 0.015), and decreased IgG anti-ApoB-100^1662–1683^ MDA levels (1.45-fold, *p* < 0.00001). IgG anti-FINC^959–984^ levels were also elevated (1.11-fold, *p* = 0.002). For C4A/C4B^167–187^, IgM responses showed no significant differences, whereas IgG anti-C4A/C4B^167–187^ levels were increased (1.31-fold, *p* < 0.00001) and IgG anti-C4A/C4B^167–187^ MDA levels were decreased (1.38-fold, *p* = 0.0003).

### 3.5 Independent Associations of Standardized MDA, MDA-Protein Adducts, and Autoantibody Isotypes in T2DM/CAD (<70% vs ≥70% Stenosis)

The study classified T2DM and T2DM/CAD < 70% stenosis as lower risk, whereas T2DM/CAD ≥ 70% stenosis as higher risk. Associations between 1-SD increases in feature levels and CAD severity were tested using multivariate logistic regression (model C), adjusted for age, sex, LDL-c, HbA1c, and PCr. Under strict thresholds (*p* < 0.05, *q* < 0.05, power >0.8), only three features remained independently associated with T2DM/CAD severity in the adjusted model. Table 3 outlines the associations between oxidative stress markers and autoantibody isotypes and advanced disease. A 1-SD increase in MDA (OR 3.148) and MDA-protein adducts (OR 2.090) served as a strong indicator of advanced disease severity. IgG anti-ApoB-100^1662–1683^ MDA (OR 0.481) showed an inverse association. IgG anti-FINC^959–984^ MDA (OR 0.616) suggested protection, although significance was marginal after FDR correction (*q* = 0.075). After adjusting for metabolic covariates, most antibody responses lost significance, indicating confounding by lipid, glycemic, and renal disturbances rather than independent predictive value.

**Table 3:** Standardized Odds Ratios for T2DM/CAD Progression (<70% and ≥ 70% Stenosis) Associated with MDA, MDA-Protein Adducts, and Autoantibodies

			T2DM/CAD^b^						
feature^a^			stenosis (%)		multivariate logistic regression^c^				
			<70	≥70					
		Cutoff	*n* = 105	*n* = 60	Model	Per 1-SD OR (95%CI)	*p*	*q*	Power
MDA (nmol/L)	≤	271.96	44	10	A	3.607 (1.831, 6.850)	0.0002	0.001	0.970
	>	271.96	61	50	B	3.505 (1.794, 6.845)	0.0002	0.001	0.970
					C	3.148 (1.620, 6.115)	0.001	0.006	0.970
MDA-protein adduct (ng/mL)	≤	241.79	87	40	A	2.417 (1.504, 3.462)	0.0001	0.001	0.996
	>	241.79	18	20	B	2.290 (1.497, 3.501)	0.0001	0.001	0.996
					C	2.090 (1.354, 3.226)	0.001	0.006	0.996
IgM anti-ApoB-100^1662–1683^ (ng/mL)	>	4014.16	26	16	A	0.905 (0.646, 1.276)	0.577	0.736	0.086
	≤	4014.16	79	44	B	0.865 (0.608, 1.231)	0.421	0.583	0.086
					C	0.816 (0.573, 1.161)	0.258	0.357	0.086
IgM anti-ApoB-100^1662–1683^ MDA (ng/mL)	>	4014.16	26	18	A	0.767 (0.479, 1.033)	0.073	0.164	0.448
	≤	4014.16	79	42	B	0.654 (0.440, 0.973)	0.036	0.082	0.448
					C	0.744 (0.492, 1.124)	0.159	0.303	0.448
IgG anti-ApoB-100^1662–1683^ (ng/mL)	≤	389.28	63	34	A	1.147 (0.823, 1.539)	0.461	0.693	0.115
	>	389.28	42	26	B	1.053 (0.761, 1.459)	0.754	0.851	0.115
					C	1.085 (0.758, 1.554)	0.654	0.785	0.115
IgG anti-ApoB-100^1662–1683^ MDA (ng/mL)	>	1959.01	41	34	A	0.490 (0.324, 0.716)	0.0003	0.001	0.975
	≤	1959.01	64	26	B	0.462 (0.306, 0.698)	0.0002	0.001	0.975
					C	0.481 (0.309, 0.749)	0.001	0.006	0.975
IgM anti-FINC^959–984^ (ng/mL)	≤	1022.92	75	44	A	0.909 (0.680, 1.317)	0.743	0.787	0.062
	>	1022.92	30	16	B	0.963 (0.672, 1.379)	0.835	0.885	0.062
					C	1.016 (0.709, 1.455)	0.932	0.932	0.062
IgM anti-FINC^959–984^ MDA (ng/mL)	>	1717.0	40	25	A	0.862 (0.599, 1.262)	0.462	0.693	0.115
	≤	1717.0	65	35	B	0.770 (0.513, 1.156)	0.207	0.373	0.115
					C	0.775 (0.536, 1.121)	0.176	0.303	0.115
IgG anti-FINC^959–984^ (ng/mL)	>	336.83	83	48	A	0.943 (0.663, 1.262)	0.587	0.736	0.084
	≤	336.83	22	12	B	0.868 (0.621, 1.212)	0.405	0.583	0.084
					C	0.715 (0.489, 1.044)	0.082	0.212	0.084
IgG anti-FINC^959–984^ MDA (ng/mL)	≤	2971.2	65	44	A	0.590 (0.429, 0.872)	0.007	0.020	0.802
	>	2971.2	40	16	B	0.550 (0.379, 0.800)	0.002	0.008	0.802
					C	0.616 (0.414, 0.916)	0.017	0.075	0.802
IgM anti-C4A/C4B^167–187^ (ng/mL)	>	158.76	94	54	A	0.949 (0.722, 1.366)	0.967	0.967	0.050
	≤	158.76	11	6	B	0.948 (0.676, 1.329)	0.757	0.851	0.050
					C	0.970 (0.687, 1.368)	0.861	0.912	0.050
IgM anti-C4A/C4B^167–187^ MDA (ng/mL)	>	1716.86	64	40	A	0.780 (0.535, 1.095)	0.143	0.285	0.317
	≤	1716.86	41	20	B	0.713 (0.489, 1.039)	0.078	0.156	0.317
					C	0.825 (0.553, 1.232)	0.347	0.446	0.317
IgG anti-C4A/C4B^167–187^ (ng/mL)	≤	315.3	53	23	A	1.640 (1.180, 2.320)	0.004	0.013	0.862
	>	315.3	52	37	B	1.554 (1.093, 2.207)	0.014	0.041	0.862
					C	1.325 (0.900, 1.950)	0.154	0.303	0.862
IgG anti-C4A/C4B^167–187^ MDA (ng/mL)	>	1014.63	55	39	A	0.592 (0.404, 0.892)	0.011	0.030	0.747
	≤	1014.63	50	21	B	0.607 (0.405, 0.910)	0.016	0.041	0.747
					C	0.641 (0.407, 1.007)	0.054	0.194	0.747

a T2DM, type 2 diabetes mellitus; CAD, coronary artery disease; T2DM/CAD ≥ 70% stenosis, T2DM patients with obstructive CAD; T2DM/CAD < 70% stenosis, T2DM patients with nonobstructive CAD; per 1-SD OR, odds ratios per one standard deviation; CI, confidence interval. The sample size of 105 individuals with T2DM/CAD < 70% stenosis was defined as comprising 60 individuals with T2DM and 45 with T2DM/CAD < 70% stenosis.

b MDA, malondialdehyde; MDA-protein adduct, malondialdehyde-modified protein adduct; ApoB-100, apolipoprotein B-100; FINC, fibronectin; C4A/C4B, complement C4A/complement C4B; LDL-c, low-density lipoprotein-cholesterol; HbA1c, glycated hemoglobin; PCr, plasma creatinine. Model A, unadjusted; model B, adjusted for age and sex; model C, adjusted for age and sex, LDL-c, HbA1c, and PCr.

### 3.6 SHAP-Based Feature Selection and Model Construction

Feature selection used a multimodel consensus. Of 21 candidates, 19 advanced, with IgG anti-ApoB-100^1662–1683^ MDA and MDA-protein adducts emerging as the most stable predictors, meeting strict thresholds (SHAP-Cov (95% CI), Brier score <0.2, VIF < 5) in Table S4. IgG anti-ApoB-100^1662–1683^ MDA ranked first (AUC 0.803–0.876), while the MDA-protein adduct ranked sixth (AUC 0.757–0.840). Other strong features, including IgG anti-FINC^959–984^ MDA and MDA, were excluded for failing SHAP-Cov significance. IgG anti-ApoB-100^1662–1683^ MDA was selected as the seed feature due to its discriminative strength and statistical validity.

In Table 4, across the four clinical comparison contexts, the four machine learning models consistently outperformed LR in discrimination, calibration alignment, and NB at 20%. RF and LightGBM delivered the most pronounced gains in the initial two T2DM baseline cohorts. In contrast, in the latter two, more challenging contexts, LR maintained competitive performance and, in certain instances, surpassed several boosting frameworks. RF demonstrated excellent performance in distinguishing T2DM from T2DM/CAD ≥ 70% stenosis (mean AUC, 0.933; pooled AUC, 0.958; sensitivity, 90.0%; F1-score, 88.4%; specificity, 86.6%; Brier score, 0.089; calibration slope, 1.203; NB at 20%, 0.433), and the SHAP-derived feature set comprised IgG anti-ApoB-100^1662–1683^ MDA, IgM anti-FINC^959–984^, and IgG anti-C4A/B^167–187^. No feature had a VIF > 10, whereas IgM anti-FINC^959–984^ MDA, IgM anti-APOB-100^1662–1683^ MDA, and CRI I were excluded due to ρ > 0.60. Furthermore, LightGBM showed the best performance in distinguishing T2DM from T2DM/CAD < 70% stenosis (mean AUC, 0.946; pooled AUC, 0.942; sensitivity, 89.0%; F1-score, 87.9%; specificity, 90.1%; Brier score, 0.081; calibration slope, 1.008; NB at 20%, 0.367), and the SHAP-derived feature set included IgG anti-ApoB-100^1662–1683^ MDA, IgM anti-ApoB-100^1662–1683^, and IgG anti-C4A/B^167–187^. No feature had a VIF > 10; however, IgG anti-FINC^959–984^ MDA, IgG anti-C4A/B^167–187^ MDA, IgM anti-FINC^959–984^ MDA, IgM anti-APOB^1662–1683^ MDA, and CRI I were excluded due to ρ > 0.60. Discrimination and calibration were lower for T2DM and T2DM/CAD < 70% vs T2DM/CAD ≥ 70% stenosis (pooled AUC, 0.742–0.769; Brier scores, 0.175–0.193) and for T2DM/CAD < 70% vs ≥ 70% stenosis (pooled AUC, 0.545–0.641; Brier scores, 0.230–0.251). An EPV of 15–20 was used to ensure statistically robust performance and reduce the risk of overfitting.

**Table 4:** Comparative Evaluation of RF, XGBoost, LightGBM, CatBoost, and LR Performance Metrics and Clinical Utility for Distinguishing T2DM and T2DM/CAD

T2DM vs T2DM/CAD < 70% stenosis^a^									
model/feature	EPV	mean AUC (95% CI)	pooled AUC (95% CI)	sensitivity (95% CI)	F1-score (95% CI)	specificity (95% CI)	Brier score (95% CI)	CS (95% CI)	NB at 20% (95% CI)
RF	15	0.927 (0.904, 0.949)	0.926 (0.857, 0.980)	89.0% (79.2%, 97.7%)	88.9% (81.2%, 95.0%)	91.7% (83.9%, 98.3%)	0.093 (0.060, 0.131)	1.213 (1.026, 1.370)	0.369 (0.276, 0.469)
Selected SHAP-derived feature set: IgG anti-ApoB-100^1662–1683^ MDA, MDA-protein adduct, IgG anti-C4A/B^167–187^									
Feature (VIF > 10): MDA-protein adduct, IgG anti-C4A/B^167–187^									
Excluded feature (ρ > 0.60): IgG anti-FINC^959–984^ MDA, IgG anti-C4A/B^167–187^ MDA, IgM anti-FINC^959–984^ MDA, IgM anti-APOB^1662–1683^ MDA, CRI I									
XGBoost	15	0.939 (0.918, 0.959)	0.927 (0.859, 0.982)	84.5% (73.2%, 94.7%)	87.3% (79.0%, 94.1%)	93.5% (86.7%, 98.5%)	0.087 (0.051, 0.128)	1.053 (0.831, 1.231)	0.352 (0.260, 0.445)
Selected SHAP-derived feature set: IgG anti-ApoB-100^1662–1683^ MDA, MDA-protein adduct, IgG anti-C4A/B^167–187^									
Feature (VIF > 10): MDA-protein adduct, IgG anti-C4A/B^167–187^									
Excluded feature (ρ > 0.60): IgG anti-FINC^959–984^ MDA, IgG anti-C4A/B^167–187^ MDA, IgM anti-FINC^959–984^ MDA, IgM anti-APOB^1662–1683^ MDA, CRI I									
LightGBM	15	0.946 (0.927, 0.965)	0.942 (0.884, 0.986)	89.0% (79.2%, 97.7%)	87.9% (80.0%, 94.5%)	90.1% (82.1%, 96.8%)	0.081 (0.045, 0.123)	1.008 (0.789, 1.196)	0.367 (0.271, 0.467)
Selected SHAP-derived feature set: IgG anti-ApoB-100^1662–1683^ MDA, IgM anti-ApoB-100^1662–1683^, IgG anti-C4A/B^167–187^									
Feature (VIF > 10): N/A									
Excluded feature (ρ > 0.60): IgG anti-FINC^959–984^ MDA, IgG anti-C4A/B^167–187^ MDA, IgM anti-FINC^959–984^ MDA, IgM anti-APOB^1662–1683^ MDA, CRI I									
CatBoost	15	0.929 (0.901, 0.954)	0.927 (0.858, 0.981)	86.7% (75.6%, 95.7%)	87.6% (79.5%, 94.1%)	91.7% (83.9,% 98.1%)	0.092 (0.056, 0.135)	1.079 (0.850, 1.278)	0.359 (0.271, 0.457)
Selected SHAP-derived feature set: IgG anti-ApoB-100^1662–1683^ MDA, MDA-protein adduct, IgG anti-C4A/B^167–187^									
Feature (VIF > 10): MDA-protein adduct, IgG anti-C4A/B^167–187^									
Excluded feature (ρ > 0.60): IgG anti-FINC^959–984^ MDA, IgG anti-C4A/B^167–187^ MDA, IgM anti-FINC^959–984^ MDA, IgM anti-APOB^1662–1683^ MDA, CRI I									
LR	15	0.837 (0.795, 0.876)	0.883 (0.813, 0.943)	75.7% (63.0%, 87.5%)	77.1% (66.7%, 86.0%)	84.9% (75.4%, 93.5%)	0.138 (0.104, 0.178)	1.123 (0.882, 1.353)	0.303 (0.212, 0.395)
Selected SHAP-derived feature set: IgG anti-ApoB-100^1662–1683^ MDA, IgG anti-C4A/B^167–18^, CRI II									
Feature (VIF > 10): N/A									
Excluded feature (ρ > 0.60): IgG anti-FINC^959–984^ MDA, IgG anti-C4A/B^167–187^ MDA, IgM anti-FINC^959–984^ MDA, IgM anti-APOB^1662–1683^ MDA, CRI I									

T2DM vs T2DM/CAD ≥ 70% stenosis									
model/feature	EPV	mean AUC (95% CI)	pooled AUC (95% CI)	sensitivity (95% CI)	F1-score (95% CI)	specificity (95% CI)	Brier score (95% CI)	CS (95% CI)	NB at 20% (95% CI)
RF	20	0.933 (0.908, 0.956)	0.958 (0.922, 0.985)	90.0% (81.4%, 96.8%)	88.4% (81.8%, 93.8%)	86.6% (77.6%, 94.2%)	0.089 (0.065, 0.115)	1.203 (1.028, 1.355)	0.433 (0.338, 0.525)
Selected SHAP-derived feature set: IgG anti-ApoB-100^1662–1683^ MDA, IgM anti-FINC^959–984^, IgG anti-C4A/B^167–187^									
Feature (VIF > 10): N/A									
Excluded feature (ρ > 0.60): IgM anti-FINC^959–984^ MDA, IgM anti-APOB-100^1662–1683^ MDA, CRI I									
XGBoost	20	0.920 (0.895, 0.944)	0.949 (0.904, 0.982)	90.1% (81.8%, 96.9%)	88.5% (82.0%, 94.0%)	86.6% (77.6%, 94.4%)	0.086 (0.057, 0.119)	1.098 (0.923, 1.247)	0.433 (0.340, 0.525)
Selected SHAP-derived feature set: IgG anti-ApoB-100^1662–1683^ MDA, IgM anti-FINC^959–984^, IgG anti-C4A/B^167–187^									
Feature (VIF > 10): N/A									
Excluded feature (ρ > 0.60): IgM anti-FINC^959–984^ MDA, IgM anti-APOB-100^1662–1683^ MDA, CRI I									
LightGBM	20	0.920 (0.900, 0.941)	0.923 (0.867, 0.968)	86.7% (77.2%, 94.9%)	88.8% (82.1%, 94.4%)	91.7% (84.1%, 98.1%)	0.095 (0.058, 0.137)	1.042 (0.849, 1.217)	0.423 (0.331, 0.513)
Selected SHAP-derived feature set: IgG anti-ApoB-100^1662–1683^ MDA, IgM anti-FINC^959–984^, IgG anti-C4A/B^167–187^									
Feature (VIF > 10): N/A									
Excluded feature (ρ > 0.60): IgM anti-FINC^959–984^ MDA, IgM anti-APOB-100^1662–1683^ MDA, CRI I									
CatBoost	20	0.887 (0.861, 0.915)	0.882 (0.810, 0.944)	78.4% (66.7%, 88.3%)	80.9% (72.2%, 88.2%)	85.0% (75.0%, 93.4%)	0.131 (0.092, 0.172)	1.041 (0.837, 1.236)	0.373 (0.281, 0.469)
Selected SHAP-derived feature set: IgG anti-ApoB-100^1662–1683^ MDA, IgM anti-FINC^959–984^, IgG anti-C4A/B^167–187^									
Feature (VIF > 10): N/A									
Excluded feature (ρ > 0.60): IgM anti-FINC^959–984^ MDA, IgM anti-APOB-100^1662–1683^ MDA, CRI I									
LR	20	0.852 (0.832, 0.872)	0.858 (0.784, 0.924)	76.6% (65.1%, 87.5%)	75.8% (66.7%, 83.9%)	74.9% (63.6%, 86.0%)	0.153 (0.115, 0.194)	1.061 (0.858, 1.264)	0.351 (0.256, 0.448)
Selected SHAP-derived feature set: IgG anti-APOB^1662–1683^ MDA, Age, CRI II									
Feature (VIF > 10): N/A									
Excluded feature (ρ > 0.60): IgM anti-FINC^959–984^ MDA, IgM anti-APOB-100^1662–1683^ MDA, CRI I									

T2DM and T2DM/CAD < 70% stenosis vs T2DM/CAD ≥ 70% stenosis^a^									
model/feature	EPV	mean AUC (95% CI)	pooled AUC (95% CI)	sensitivity (95% CI)	F1-score (95% CI)	specificity (95% CI)	Brier score (95% CI)	CS (95% CI)	NB at 20% (95% CI)
**RF**	20	0.764 (0.731, 0.796)	0.783 (0.700, 0.856)	48.4% (35.7%, 61.4%)	56.2% (44.0%, 67.9%)	86.7% (79.4%, 92.9%)	0.175 (0.147, 0.205)	1.240 (1.063, 1.414)	0.155 (0.094, 0.220)
Selected SHAP-derived feature set: IgG anti-ApoB-100^1662–1683^ MDA, PCr, CRI II									
Feature (VIF > 10): N/A									
Excluded feature (ρ > 0.60): CRI I, IgM anti-APOB-100^1662–1683^ MDA, IgG anti-FINC^959–984^ MDA, IgM anti-C4A/B^167–187^ MDA									
XGBoost	20	0.734 (0.696, 0.771)	0.769 (0.683, 0.843)	51.7% (38.6%, 65.0%)	57.8% (45.8%, 68.9%)	84.8% (77.1%, 91.4%)	0.179 (0.146, 0.215)	0.911 (0.645, 1.149)	0.164 (0.103, 0.232)
Selected SHAP-derived feature set: IgG anti-ApoB-100^1662–1683^ MDA, PCr, CRI II									
Feature (VIF > 10): N/A									
Excluded feature (ρ > 0.60): CRI I, IgM anti-APOB-100^1662–1683^ MDA, IgG anti-FINC^959–984^ MDA, IgM anti-C4A/B^167–187^ MDA									
LightGBM	20	0.715 (0.676, 0.752)	0.766 (0.689, 0.839)	45.2% (32.8%, 57.8%)	53.9% (41.9%, 65.3%)	87.6% (80.8%, 93.5%)	0.185 (0.153, 0.219)	0.867 (0.497, 1.172)	0.145 (0.088, 0.205)
Selected SHAP-derived feature set: IgG anti-ApoB-100^1662–1683^ MDA, PCr, CRI II									
Feature (VIF > 10): N/A									
Excluded feature (ρ > 0.60): CRI I, IgM anti-APOB-100^1662–1683^ MDA, IgG anti-FINC^959–984^ MDA, IgM anti-C4A/B^167–187^ MDA									
CatBoost	20	0.716 (0.685, 0.747)	0.748 (0.666, 0.825)	46.6% (33.9%, 58.9%)	53.1% (41.0%, 63.8%)	83.9% (76.2%, 90.3%)	0.193 (0.167, 0.222)	1.041 (0.562, 1.485)	0.144 (0.086, 0.203)
Selected SHAP-derived feature set: IgG anti-ApoB-100^1662–1683^ MDA, PCr, CRI II									
Feature (VIF > 10): N/A									
Excluded feature (ρ > 0.60): CRI I, IgM anti-APOB-100^1662–1683^ MDA, IgG anti-FINC^959–984^ MDA, IgM anti-C4A/B^167–187^ MDA									
LR	20	0.748 (0.708, 0.785)	0.742 (0.657, 0.819)	45.0% (32.8%, 57.4%)	54.3% (41.9%, 65.5%)	88.6% (81.7%, 94.4%)	0.192 (0.163, 0.223)	0.867 (0.145, 1.252)	0.146 (0.089, 0.206)
Selected SHAP-derived feature set: IgG anti-ApoB-100^1662–1683^ MDA, PCr, CRI II									
Feature (VIF > 10): N/A									
Excluded feature (ρ > 0.60): CRI I, IgM anti-APOB-100^1662–1683^ MDA, IgG anti-FINC^959–984^ MDA, IgM anti-C4A/B^167–187^ MDA									

T2DM/CAD < 70% stenosis vs T2DM/CAD ≥ 70% stenosis^a^									
model/feature	EPV	mean AUC (95% CI)	pooled AUC (95% CI)	sensitivity (95% CI)	F1-score (95% CI)	specificity (95% CI)	Brier score (95% CI)	CS (95% CI)	NB at 20% (95% CI)
RF	15	0.607 (0.555, 0.660)	0.641 (0.530, 0.745)	76.8% (66.0%, 87.2%)	70.7% (61.8%, 79.1%)	46.6% (31.7%, 61.5%)	0.230 (0.198, 0.264)	0.700 (0.096, 1.254)	0.381 (0.279, 0.490)
Selected SHAP-derived feature set: IgG anti-ApoB-100^1662–1683^ MDA, IgG anti-FINC^959–984^, Sex									
Feature (VIF > 10): N/A									
Excluded feature (ρ > 0.60): CRI I, IgM anti-APOB-100^1662–1683^ MDA, IgM anti-C4A/B^167–187^ MDA									
XGBoost	15	0.604 (0.569, 0.643)	0.632 (0.515, 0.739)	68.4% (55.2%, 80.6%)	64.4% (53.7%, 74.5%)	42.2% (27.5%, 58.1%)	0.240 (0.203, 0.278)	0.921 (0.396, 1.195)	0.329 (0.224, 0.443)
Selected SHAP-derived feature set: IgG anti-ApoB-100^1662–1683^ MDA, MDA-protein adduct, IgM anti-FINC^959–984^									
Feature (VIF > 10): N/A									
Excluded feature (ρ > 0.60): CRI I, IgM anti-APOB-100^1662–1683^ MDA, IgM anti-C4A/B^167–187^ MDA									
LightGBM	15	0.558 (0.515, 0.598)	0.555 (0.441, 0.667)	71.7% (59.3%, 82.5%)	64.0% (53.3%, 73.1%)	31.0% (18.0%, 44.9%)	0.251 (0.220, 0.283)	0.005 (−0.926, 0.948)	0.335 (0.224, 0.445)
Selected SHAP-derived feature set: IgG anti-ApoB-100^1662–1683^ MDA, IgG anti-FINC^959–984^, IgG anti-FINC^959–984^ MDA									
Feature (VIF > 10): N/A									
Excluded feature (ρ > 0.60): CRI I, IgM anti-APOB-100^1662–1683^ MDA, IgM anti-C4A/B^167–187^ MDA									
CatBoost	15	0.518 (0.475, 0.559)	0.545 (0.434, 0.662)	90.1% (82.1%, 96.8%)	70.5% (61.5%, 78.3%)	13.4% (4.3%, 24.2%)	0.245 (0.228, 0.263)	1.005 (−1.051, 3.257)	0.421 (0.305, 0.538)
Selected SHAP-derived feature set: IgG anti-ApoB-100^1662–1683^ MDA, MDA, IgG anti-FINC^959–984^ MDA									
Feature (VIF > 10): N/A									
Excluded feature (ρ > 0.60): CRI I, IgM anti-APOB-100^1662–1683^ MDA, IgM anti-C4A/B^167–187^ MDA									
LR	15	0.583 (0.536, 0.631)	0.613 (0.499, 0.721)	83.2% (73.0%, 92.3%)	70.2% (61.2%, 78.7%)	29.1% (16.3%, 43.1%)	0.234 (0.214, 0.255)	1.418 (−0.071, 2.152)	0.399 (0.290, 0.510)
Selected SHAP-derived feature set: IgG anti-APOB-100^1662–1683^ MDA, Sex, TyG Index									
Feature (VIF > 10): N/A									
Excluded feature (ρ > 0.60): CRI I, IgM anti-APOB-100^1662–1683^ MDA, IgM anti-C4A/B^167–187^ MDA									

a T2DM, type 2 diabetes mellitus; CAD, coronary artery disease; T2DM/CAD ≥ 70% stenosis, T2DM patients with obstructive CAD; T2DM/CAD < 70% stenosis, T2DM patients with nonobstructive CAD; MDA, malondialdehyde; MDA-protein adduct, malondialdehyde-modified protein adduct; ApoB-100, apolipoprotein B-100; FINC, fibronectin; C4A/C4B, complement C4A/complement C4B; RF, random forest; XGBoost: eXtreme Gradient Boosting; LightGBM, light gradient boosting machine; CatBoost, categorical boosting; LR, logistic regression; EPV, events per variable; mean AUC, mean area under the receiver operating characteristic curve (AUC) across the fivefold cross validation; pooled AUC, pooled area under the receiver operating characteristic curve from out-of-fold (OOF) predictions; CS, calibration slope; NB at 20%, net benefit at a 20% threshold probability; CI, confidence interval; SHAP, Shapley additive explanations; VIF, variance inflation factor; ρ, Spearman’s rank correlation; N/A, not available; PCr, plasma creatinine; TyG index, triglyceride–glucose index, calculated as the natural logarithm (ln) of [fasting triglycerides (mg/dL) × fasting glucose (mg/dL)/2]; HbA1c/HDL-c ratio, ratio of glycated hemoglobin (%) to high-density lipoprotein-cholesterol (mg/dL); CRI I, Castelli risk index I, calculated by dividing total cholesterol by high-density lipoprotein-cholesterol (TC/HDL-c); CRI II, Castelli risk index II, determined as the ratio of low-density lipoprotein-cholesterol to high-density lipoprotein-cholesterol (LDL-c/HDL-c).

Across five models with T2DM vs T2DM/CAD ≥ 70% stenosis and T2DM vs T2DM/CAD < 70% stenosis, IgG anti-C4A/B^167–187^, IgM anti-FINC^959–984^, MDA-protein adduct, and age were positive predictors, while IgG anti-ApoB-100^1662–1683^ MDA, IgM anti-ApoB-100^1662–1683^, and CRI II was negative predictors. SHAP-Cov values (1.058–1.468), below the stability threshold of 1.5, confirmed consistency and reliability in the prediction of severe CAD (Table S5). Individual outer-fold logs from repeated stratified nested cross validation are documented in Table S6. Calibration curves are plotted in Figure S3.

### 3.7 SHAP-Based Model Interpretation Reveals Oxidative–Immune Signatures Differentiating T2DM and T2DM/CAD ≥ 70% Stenosis

In Figure 2A, SHAP bar plot analysis identified IgG anti-C4A/B^167–187^ as the strongest predictor (mean |SHAP| 0.210), followed by IgG anti-ApoB-100^1662–1683^ MDA (0.155) and IgM anti-FINC^959–984^ (0.145). In Figure 2B, the direction of these associations is illustrated. The SHAP summary plot showed that IgG anti-C4A/B^167–187^ levels (red dots) were positively correlated with severe stenosis, while high IgG anti-ApoB-100^1662–1683^ MDA levels (red dots) were inversely related, suggesting protection. IgM anti-FINC^959–984^ also correlated positively, although with greater variability. SHAP plots revealed nonlinear thresholds in the relationship between autoantibody levels and predictions. A sigmoidal relationship was observed between IgG anti-C4A/B^167–187^ levels and their contribution to the model. Concentrations below ∼330 were associated with a negative impact (SHAP −0.35), with an inflection near 340 and a positive plateau (+0.2 to +0.5) beyond 360. Interaction coloring indicated that elevated IgM anti-FINC^959–984^ slightly reduced the positive effect at high IgG levels (Figure 2C). IgG anti-APOB^1662–1683^ MDA showed a downward nonlinear trend in its contribution to the model. Concentrations below 1400 contributed positively (SHAP +0.1 to +0.25), with an inflection near 1500 marking the transition to negative impact. Values above 1700 consistently reduced prediction scores (SHAP −0.1 to −0.25). The negative effect was most clear in patients with lower IgG anti-C4A/B^167–187^ (Figure 2D). IgM anti-FINC^959–984^ exhibited a U-shaped pattern in its contribution to the model. Low (<250) and high (>1150) concentrations increased risk (SHAP up to +0.40), while the midrange (250–1100) was protective (SHAP −0.25). Interaction coloring suggested that high IgG anti-C4A/B^167–187^ reduced protection in the intermediate range, whereas low IgG increased risk at high IgM values (Figure 2E). These immune-oxidative signatures were interpreted as reflecting complement activation, lipid oxidation, and matrix remodeling.

**Figure 2:**
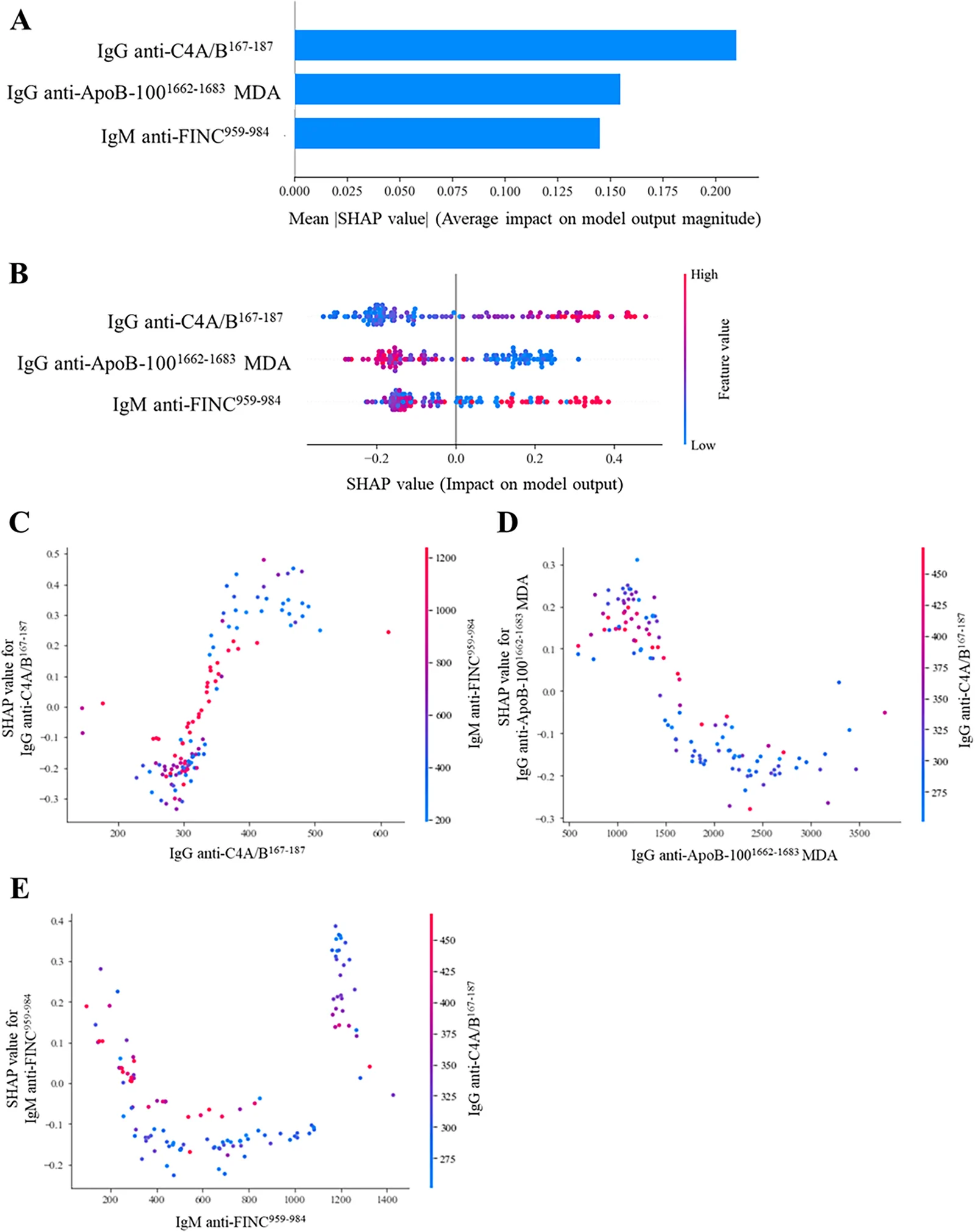
SHAP-based feature importance and nonlinear associations of immune biomarkers in T2DM and T2DM/CAD ≥ 70% stenosis. (A) SHAP bar plot showing global feature importance ranking. The *x*-axis shows mean |SHAP value|, reflecting each feature’s average contribution. Ranked from the highest to lowest, IgG anti-C4A/B^167–187^, IgG anti-ApoB-100–100^1662–1683^ MDA, and IgM anti-FINC^959–984^. (B) SHAP summary plot showing the distribution of SHAP values for each feature and direction. Each dot represents a sample. The color gradient runs from red (high concentration) to blue (low concentration) for the feature value, and the *x*-axis indicates the SHAP value, representing the model’s impact. Positive SHAP values indicate a higher risk of severe stenosis. SHAP dependence plots for (C) IgG anti-C4A/B^167–187^, (D) IgG anti-ApoB-100^1662–1683^ MDA, and (E) IgM anti-FINC^959–984^. These plots reveal threshold dynamics and synergistic effects; the color scales denote interacting feature values, used here to uncover underlying pairwise dependencies. SHAP; Shapley additive explanations; mean |SHAP value|, mean absolute SHAP value; ApoB-100, Apolipoprotein B-100; FINC, Fibronectin; C4A, Complement C4A; C4B, Complement C4B; MDA, Malondialdehyde.

Figure 3 illustrates a SHAP analysis of humoral markers influencing predictions of coronary narrowing. Two misclassified cases offered preliminary insight into the classifier’s limitations. For the false-positive case (sample ID 8), the model shifted from a baseline probability of 0.503 to a final risk of 0.35. The principal protective contributor was the reduced level of IgG anti-C4A/B^167–187^ (299.8; SHAP −0.25), which was partially offset by a risk-elevating effect from low IgG anti-APOB^1662–1683^ MDA (1171.1; SHAP + 0.09) and a marginal positive increment from elevated IgM anti-FINC^959–984^ (1285.2; SHAP + 0.01), as shown in Figure 3A,B. In the false-negative case (sample ID 68), the model shifted from a baseline probability of 0.503 to a final risk of 0.64; the principal elevating contributor was the increased level of IgM anti-FINC^959–984^ (230.4; SHAP + 0.23), which was partially offset by a protective, risk-lowering effect from IgG anti-C4A/B^167–187^ (295.5; SHAP −0.11), along with a marginal positive increment from IgG anti-APOB^1662–1683^ MDA (3293.0; SHAP + 0.02) in Figure 3C,D. The waterfall plot (Figure 3A,C) sequentially displays the additive structural weights, whereas the force plot (Figure 3B,D) illustrates the horizontal equilibrium formed between opposing immune-oxidative signals. Together, these visualizations highlight the complex interplay underlying severe CAD risk, in which autoantibody isotypes may either mitigate or exacerbate systemic inflammation. These case-level findings are consistent with recent SHAP-based explainable ML studies, underscoring biomarker relevance and the limitations of clinical prediction models.

**Figure 3:**
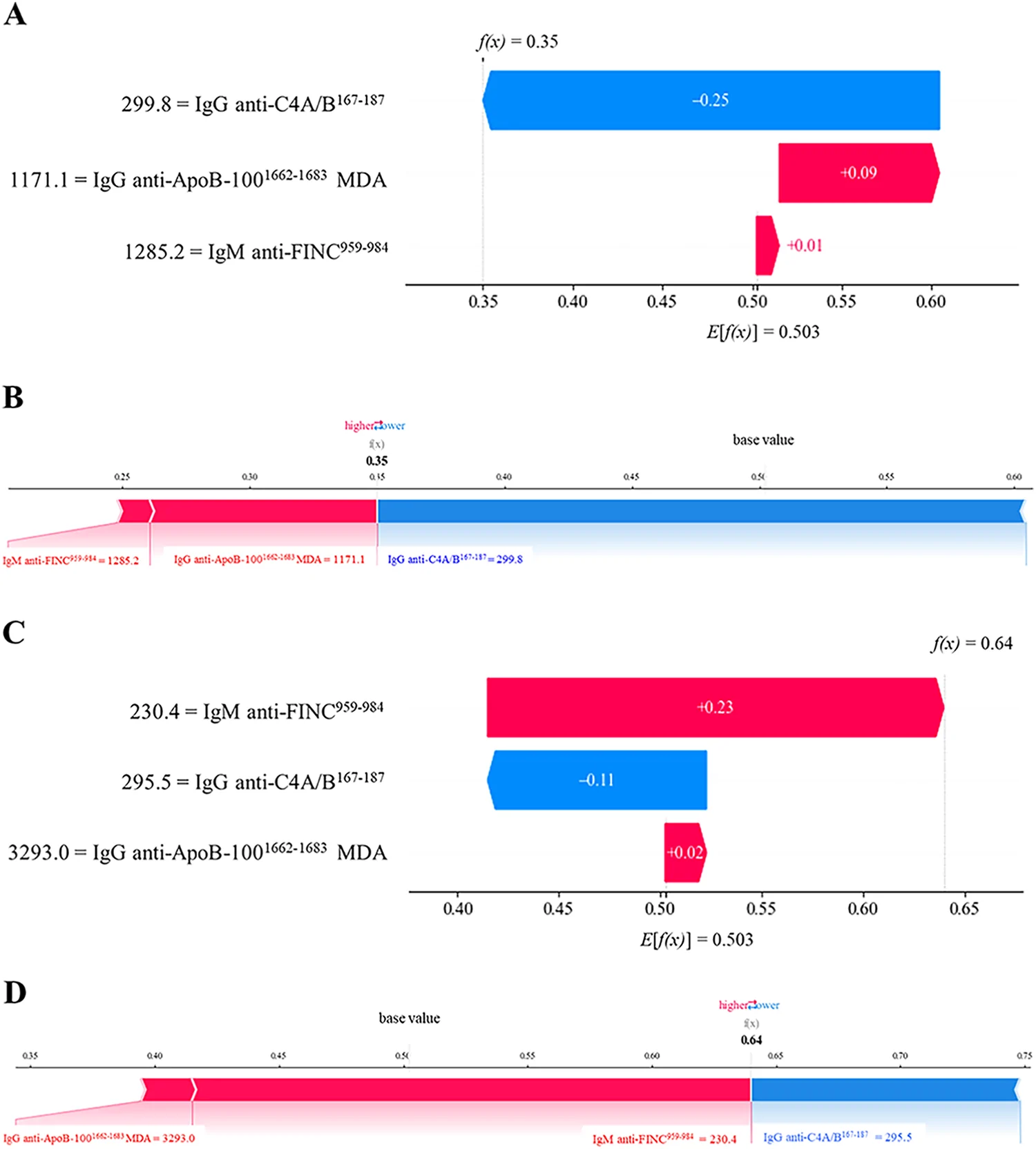
SHAP waterfall and force plots illustrating hard false-positive (FP) and false-negative (FN) cases in distinguishing T2DM from T2DM/CAD ≥ 70% stenosis. (A, B) Waterfall and force plots for sample ID 8, a FP case with a predicted T2DM/CAD ≥ 70% stenosis probability but clinically T2DM. (C, D) Waterfall and force plots for sample ID 68, an FN case with a predicted T2DM probability but clinically T2DM/CAD ≥ 70% stenosis. SHAP waterfall plots illustrate the incremental contribution of each feature to the final prediction *f*(*x*) for specific patient cases (A[Fig fig3], C). SHAP force plots (B[Fig fig3], D) show the push–pull dynamics between proatherogenic (red) and protective (blue) immune markers. *E*[*f*(*x*)] represents the base value (expected average model output), while *f*(*x*) represents the individualized risk score for the specific patient.

### 3.8 Model Fairness Assessment

Table S7 presents the comparative discriminative performance, calibration, and clinical utility of CatBoost and LR across sociodemographic strata. In the overall cohort, CatBoost achieved markedly superior discrimination (pooled AUC 0.975) compared with LR (pooled AUC, 0.922), with statistical significance (*p* = 0.0031). Stratified analyses indicated that CatBoost maintained a significant advantage in the male (*p* = 0.0061) and younger (<65 years, *p* = 0.021) cohorts, while differences narrowed and became nonsignificant in the female and older (≥65 years) strata, despite consistently higher absolute AUC values. CatBoost consistently demonstrated superior calibration compared with LR (Brier score, 0.046–0.091 vs 0.121–0.175; all *p* < 0.001). Further, CatBoost consistently exhibited superior decision curve analysis at the 20% threshold compared with LR (NB at 20%, 0.453–0.731 vs 0.404–0.699; all *p* < 0.001), except in the female subgroup, where the difference did not reach statistical significance. Fairness evaluation indicated no sex-based disparities, underscoring gender stability. In contrast, age-based disparity was evident. In the older cohort, both CatBoost and LR indicated significantly better calibration (CatBoost: 0.046 vs 0.091, *p* = 0.0189; LR: 0.121 vs 0.175, *p* = 0.003) and higher NB at 20% (CatBoost: 0.731 vs 0.453, *p* = 0.001; LR: 0.699 vs 0.404, *p* = 0.001) compared to the younger cohort. These performance differences between age groups were not observed in the younger cohorts.

## 4 Discussion

Using SHAP-based ML, we built an interpretable immunometabolic model to explore potential associations between oxidative stress and adaptive immunity and CAD severity in T2DM. SHAP-derived features reveal nonlinear immunometabolic shifts as T2DM progresses to severe disease, underscoring the synergistic impact of oxidative stress and immune dysregulation within the two-hit oxidative–immune hypothesis. In the first-hit stage (T2DM vs T2DM/CAD < 70% stenosis), hyperglycemia drives oxidative stress, leading to the formation of MDA (OR 3.148) and proinflammatory MDA-protein adducts (OR 2.090), as presented in Table 3. These adducts, together with IgM/IgG anti-ApoB-100^1662–1683^, establish the earliest oxidative–immune link and initiate endothelial sensitization. IgM anti-ApoB-100^1662–1683^ (OR 0.865) and IgG anti-ApoB-100^1662–1683^ MDA (OR 0.481) showed an inverse association with CAD risk, which is hypothesized to reflect a potential protective mechanism involving the immune clearance of OSEs (Tables 3 and 4).^8,34^ IgG anti-ApoB-100^1662–1683^ MDA consistently demonstrated an inverse association with CAD risk across SHAP analyses and multivariate models (Figure 2B,D; Tables 3 and S5), highlighting its hypothesized role in removing oxidized lipoproteins and its potential to mitigate atherogenesis.^11^ To assess epitope specificity, we included nonspecific baseline controls (IgM/IgG anti-BSA) and generic lipid-peroxidation controls (IgM/IgG anti-MDA-BSA), as presented in Table 1. IgG anti-MDA-BSA showed no significant cohort-specific variation, while the target autoantibodies displayed distinct dynamics, indicating a selective immune response. In multivariate models, this protective antibody was markedly reduced in advanced obstructive CAD,^35,36^ consistent with the phenomenon of antibody exhaustion reported in severe cardiovascular disease.^36^ In contrast, IgG anti-C4A/B^167–187^ (OR 1.325) acted as a risk marker and, in combination with IgG anti-ApoB-100^1662–1683^ MDA and IgM anti-ApoB-100^1662–1683^, showed strong discriminative performance (pooled AUC 0.942) in the LightGBM model (Tables 3 and 4). All three features had SHAP-Cov values <1.5 and CIs excluding zero, confirming their predictive validity (Table S5). In the second-hit stage (T2DM vs T2DM/CAD ≥ 70% stenosis), the features shifted to IgG anti-C4A/B^167–187^ and IgM anti-FINC^959–984^ (OR 1.016), reflecting epitope spreading in the setting od chronic hyperglycemia. The immune responses extended from lipoproteins to complement and extracellular matrix components, marking diversification of autoantibodies, as presented in Tables 3 and 4.^12^ IgG anti-C4A/B^167–187^ emerged as the dominant predictor in our model. Given the established roles of complement components in vascular injury, this statistical association indicates that the autoantibody may act as a marker of sustained complement activation and MAC (C5b-9) formation. Direct experimental validation, however, is needed to confirm causality. Biochemically, MDA-lysine residues on ApoB-100 provide preferred binding sites for C4A and C4B, establishing a clear mechanistic link between lipoxidation and initiation of the classical complement cascade.^37^ Clinical studies have shown that MDA-LDL immune complexes are associated with macrovascular complications in diabetic cohorts.^38^ While cellular models have demonstrated that high-glucose oxidative stress induces AP-1 activation, adhesion molecule upregulation, and macrophage lipid uptake.^39^ MDA epitopes also recruit C1q to initiate the pathway, which is regulated by C4b-binding protein.^8^ Clinical and immunoprecipitation data indicate that these immune complexes may deposit within vascular walls during complement exhaustion, with C4b/C3b-opsonized complexes associated with foam cell formation and VCAM-1/ICAM-1 upregulation, forming a molecular-cellular network underlying atherosclerosis.^40^ SHAP analysis showed a sharp increase in risk at higher concentrations, reflecting intense complement activation and plaque instability (Figure 2A[Fig fig2],C).^13,41^ In parallel, rising concentrations of IgM anti-FINC^959–984^ were associated with elevated risk (Figure 2E), presenting a statistical correlation that serves as a candidate pathway for further investigation into its functional involvement in vascular remodeling and angiogenesis.^42^ Although direct in vitro validation is lacking in this cohort, established cell and animal models demonstrate the biological relevance of matrix modifications.^43^ MDA-FINC suppresses macrophage homeostatic signaling, while immunization against MDA-FINC elicits a protective Th2 response that reduces macrophage infiltration and decreases the lesion area by about 70%.^44^ Taken together, these multimarker baseline comparisons and rigorously adjusted regressions support the biological plausibility and translational significance of these novel modified epitopes in diabetic macrovascular complications.

Figure 3 shows misclassified cases, illustrating the dual role of oxidative–immune biomarkers in CAD risk among T2DM. In this false-positive case, diminished IgG anti-APOB-100^1662–1683^ MDA levels combined with elevated IgM anti-FINC^959–984^ levels led to misclassification into the T2DM/CAD ≥ 70% stenosis cohort. Such localized errors typically reflect complex feature interactions that alter prediction paths within tree-based ensembles.^17,45^ From a feature-engineering perspective, the issue arises from high-order interactions rather than a single weight imbalance. The anomalous low IgG anti-C4A/B^167–187^ together with the extreme IgG anti-APOB-100^1662–1683^ MDA value perturbed the classifier’s hyperplane, shifting the decision boundary.^46^ In this state, IgM anti-FINC^959–984^ contributed a positive vector, but its effect was offset by negative boundary drag. Concurrent deviations created blind spots in SHAP attribution, highlighting the need for nonlinear weighting and interaction modeling. Reliance on extreme biomarkers inflated risk, underscoring the need to integrate SHAP interpretability with conventional markers.^18,47^ These cases show immune markers can raise or lower risk depending on context, consistent with epitope spreading.^12^ SHAP revealed push–pull dynamics between protective and harmful pathways, exposing black-box limits and highlighting the need to combine immune signatures with anatomical assessment.^17,18,47^ Misclassifications suggest that immune activity may precede or diverge from stenosis, supporting immune–anatomic mismatch and underscoring potential for earlier risk detection.^17^

Compared with prior CAD models in patients with diabetes, the RF model showed superior performance (pooled AUC, 0.958), as presented in Table 4. For example, a clinical nomogram achieved AUCs of 0.753 (training, *n* = 1152) and 0.738 (validation, *n* = 238), with a sensitivity of 73.1%, a specificity of 65.0%, and Brier scores ranging from 0.153 to 0.172, indicating moderate discrimination and calibration.^48^ A LightGBM model for cardiovascular risk in T2DM (*n* = 4837) achieved an AUC of 0.802, a sensitivity of 78.4%, and a specificity of 72.1%, with SHAP analysis identifying age, BMI, blood pressure, HbA1c, renal function, and smoking as key variables.^18^ Another LightGBM model for major adverse cardiac events in patients with diabetic CKD (n = 1116) achieved an AUC of 0.740, a sensitivity of 73.9%, and an F1-score of 0.820, with SHAP analysis emphasizing eGFR, age, and the TyG index as key features.^47^ In contrast, RF distinguished T2DM from T2DM/CAD ≥ 70% stenosis with a pooled AUC of 0.958, a sensitivity of 90.0%, a specificity of 88.4%, a Brier score of 0.089, and an NB at 20% of 0.433, as presented in Table 4. This reflects a 43.3% improvement over “treat all/none,” confirming robust discrimination, calibration, and clinical utility.^16^ Overall, these findings indicate that the RF model, when integrated with SHAP, may offer complementary value in stratifying CAD severity among T2DM patients, with possible relevance for identifying obstructive disease. Although a prefiltering threshold of VIF > 10 was applied within the inner CV loop, certain high-VIF features were retained in the T2DM vs T2DM/CAD < 70% stenosis classification (Table 4). A VIF > 10 inflates parameter variance and invalidates linear inferences,^49^ yet ensemble tree learners partition nodes based on localized information gain,^45^ allowing redundant variables to yield marginal signals. Under the EPV ≥ 15 rule and modest sample size (*n* = 45, Table 1), the SFS mechanism selected these high-VIF features. They passed the SHAP stability filter (Cov <1.50), indicating discriminative validity within a restricted feature space. Optimized hyperparameters for the outer folds were *C* = 1.0, l1_ratio = 0.5, and penalty = l2 (Table S6). Since the l1_ratio is inactive when penalty = “l2” in scikit-learn, its presence reflects grid search initialization.^50^ The effective model is pure L2 regularization (ridge logistic regression). The chosen *C* = 1.0 applies moderate shrinkage, reducing variance and mitigating multicollinearity in small clinical cohorts.^51^ With prior dual-layer filtration (Spearman’s *ρ* > 0.60 and VIF > 10.0), the penalty function primarily smooths coefficients, ensuring stability and interpretability.^52^

This robustness is important as cardiovascular risks vary by age and sex, as shown in Taiwanese studies.^53^ In small strata, limited cases or homogeneous outcomes can destabilize metrics, producing extreme values. The overall AUC gap between age groups reflects fluctuations within sparse strata, while the apparent perfect discrimination in older adults is a technical artifact rathe than systemic bias.^16^ Based on the calibration and net benefit profiles, the model appears more appropriate for characterizing patients with suspected obstructive disease than for use as a general screening tool. Compared to LR, CatBoost achieved superior discrimination (pooled AUC, 0.975 vs 0.922), calibration (Brier score, 0.069 vs 0.149), and clinical utility (NB at 20%, 0.586 vs 0.545),^30^ while inline Platt scaling corrected small-sample miscalibration.^54^ Fairness analysis indicated sex equity but significant age disparity. The improvements in calibration and net benefit were observed exclusively in older cohorts (Table S7). Stable Brier scores achieved with Platt scaling addressed calibration concerns, validating reliable risk estimates across diverse profiles.^55^

This study has several limitations. First, the small sample size, restricted to Taiwanese centers, may limit generalizability and increase the risk of overfitting, although nested CV and SHAP-Cov filtering mitigated this concern. Second, the cross-sectional design of this cohort provides only a single snapshot at a given time. While it highlights distinct immunometabolic patterns linked to CAD severity, it does not allow conclusions about future cardiovascular events or prognostic risk predictions. Longitudinal studies are required to determine whether autoantibody shifts precede plaque progression. Third, although novel MDA-peptide adducts and autoantibody responses were observed, their biological relevance remains to be explored. Statistical associations and SHAP importances cannot establish causality, underscoring the need for mechanistic validation through complement activation assays and macrophage experiments in future prospective studies. Larger, multicenter prospective studies with external validation are essential to confirm robustness before clinical translation.

## 5 Conclusions

In conclusion, plasma MDA, its protein adducts, and related autoantibodies are critical indicators of concurrent CAD severity in T2DM. Integrating these immunometabolic biomarkers into a SHAP-interpretable RF model revealed push–pull dynamic interplay between protective and proatherogenic immune signals. Recognition of MDA epitopes may link oxidative stress to diabetic atherogenesis, enabling the identification of high-risk patients before the onset of anatomical stenosis, especially in cases of immune–anatomical mismatch. While larger validation cohorts are needed, nested CV and stability filtering support reliability. Overall, this framework provides a dependable approach to CAD severity classification, excelling at detecting obstructive disease. The model ensures an equitable performance across sexes, but its effectiveness varies by age. Meaningful improvements in calibration and net benefit were observed only in older patients, reflecting a more consistent biomarker alignment in that cohort. This highlights the need for a cautious approach to managing younger populations.

## Supplementary Material



## Data Availability

The acquired MS/MS fragmentation data can be accessed through the PRIDE database at ProteomeXchange under accession number PXD046723. All data supporting the findings of this study are available within the article and its Supporting Information.

## Notes

MS/MS spectra of MDA-peptide adducts in patients with T2DM, T2DM/CAD ≥ 70% stenosis, and CAD ≥ 70% stenosis (Figure S1); MDA modifications of plasma proteins (Figure S2); calibration curves for five machine learning models (Figure S3); sample size estimation (Table S1); hyperparameter search spaces and optimal configurations (Table S2); Coverage of ApoB-100, FINC, C4A, and C4B (Table S3); feature selection and ranking criteria across four machine learning models (Table S4); SHAP-based stability evaluation of predictive features (Table S5); performance metrics and hyperparameters for individual outer-fold logs (Table S6); and predictive performance and fairness assessment across subgroups (Table S7) (PDF)

## Abbreviations

T2DM: Type 2 diabetes mellitus
CAD: Coronary artery disease
T2DM/CAD ≥ 70% stenosis: T2DM patients with obstructive CAD
T2DM/CAD < 70% stenosis: T2DM patients with nonobstructive CAD
MDA: Malondialdehyde
MDA-protein adduct: Malondialdehyde-modified protein adduct
ApoB-100: Apolipoprotein B-100
FINC: Fibronectin
C4A/C4B: Complement C4A/complement C4B
RF: Random Forest
XGBoost: eXtreme Gradient Boosting
LightGBM: Light Gradient Boosting Machine
CatBoost: Categorical Boosting
MICE: multivariate imputation by chained equations
EPV: Events per variable
Mean AUC: Mean area under the receiver operating characteristic curve (AUC) across the fivefold cross validation
Pooled AUC: Pooled area under the receiver operating characteristic curve from out-of-fold (OOF) predictions
CS: Calibration slope
NB at 20%: Net benefit at a 20% threshold probability
CI: Confidence interval
SHAP: Shapley additive explanations
HbA1c: Glycated hemoglobin
TC: Total cholesterol
HDL-c: High-density lipoprotein-cholesterol
LDL-c: Low-density lipoprotein-cholesterol
TG: Triglyceride
PCr: Plasma creatinine
TyG index: Triglyceride–glucose index, calculated as the natural logarithm (ln) of [fasting triglycerides (mg/dL) × fasting glucose (mg/dL)/2]
HbA1c/HDL-c ratio: Ratio of glycated hemoglobin (%) to high-density lipoprotein-cholesterol (mg/dL)
CRI I: Castelli risk index I, calculated by dividing total cholesterol by high-density lipoprotein-cholesterol (TC/HDL-c)
CRI II: Castelli risk index II, determined as the ratio of low-density lipoprotein-cholesterol to high-density lipoprotein-cholesterol (LDL-c/HDL-c)
CBB: Coomassie brilliant blue
ELISA: Enzyme-linked immunosorbent assay
Ig: Immunoglobulin
IP: Immunoprecipitation
nano-LC–MS/MS: Nanoliquid chromatography–tandem mass spectrometry
Per 1-SD OR: Odds ratios per one standard deviation
SDS-PAGE: Sodium dodecyl sulfate-polyacrylamide gel electrophoresis
WB: Western blotting
