# Supplementary material for: Development of a Shapley Additive Explanations (SHAP)-Interpretable Model for Cardiovascular Risk Stratification in Type 2 Diabetes Mellitus: Implications of MDA-Modified Protein Adducts and Autoantibodies
Source: ACS Omega. 2026 Aug 13;11(33):50168–84. doi: 10.1021/acsomega.6c05628 (PMC13520431; doi:10.1021/acsomega.6c05628)
Supplement: Supplementary file 1 [file ao6c05628_si_001.pdf]

Supporting Information

Development of a Shapley Additive Explanations (SHAP)-Interpretable Model for Cardiovascular Risk Stratification in Type 2 Diabetes Mellitus: Implications of MDA-Modified Protein Adducts and Autoantibodies

Chien-Yi Hsu [1,2†], Shou-Cheng Lu [3†], Liang-Wei Lin [4,5], Chu-Heng Yen [3,5], Hung-Tse Lin [6,7], Shao-Chun Lin [8], Ching-Yu Lin [5,9*] and Chao-Lien Liu [5,9*]

1. Division of Cardiology, Department of Internal Medicine, School of Medicine, College of Medicine, Taipei Heart Institute, Taipei Medical University, Taipei 11031, Taiwan; chienyihsu@tmu.edu.tw
2. Division of Cardiology and Cardiovascular Research Center, Department of Internal Medicine, Taipei Medical University Hospital, Taipei 11031, Taiwan
3. Department of Laboratory Medicine, Shuang Ho Hospital, Taipei Medical University, New Taipei City 23561, Taiwan; 08395@s.tmu.edu.tw; 21488@s.tmu.edu.tw
4. Department of Laboratory Medicine, Taiwan Adventist Hospital, Taipei 10556, Taiwan; 155569@tahsda.org.tw
5. School of Medical Laboratory Science and Biotechnology, College of Medical Science and Technology, Taipei Medical University, Taipei 11031, Taiwan
6. Department of Laboratory Medicine, LinKou Chang Gung Memorial Hospital, Taoyuan 33305, Taiwan; timbaseball@cgmh.org.tw
7. Department of Medical Biotechnology and Laboratory Science, College of

Medicine, Chang Gung University, Taoyuan 33302, Taiwan
8. Department of Pharmacy, Chia Nan University of Pharmacy & Science, Tainan City 717301, Taiwan; b1401084@gm.cnu.edu.tw
9. Ph.D. Program in Medical Biotechnology, College of Medical Science and Technology, Taipei Medical University, Taipei 11031, Taiwan; cylin@tmu.edu.tw

† These first authors contributed equally to this paper.

* Corresponding author: Ph.D. Program in Medical Biotechnology, College of Medical Science and Technology, Taipei Medical University, Taipei 11031, Taiwan

Tel.: +886-2-2736-1661 ext. 3332;

E-mail: cylin@tmu.edu.tw (C.-Y. Lin)

* Corresponding author: School of Medical Laboratory Science and Biotechnology, College of Medical Science and Technology, Taipei Medical University, Taipei 11031, Taiwan.

Tel.: +886-2-2736-1661 ext. 3332;

E-mail: chaolien@tmu.edu.tw.

Running title: SHAP-Interpretable Model for CAD Risk in T2DM

**Table of Contents**	**Page No.**
Figure S1. MS/MS spectrum of the malondialdehyde (MDA)-modified peptide (MDA-peptide) adducts in patients with type 2 diabetes mellitus (T2DM), T2DM patients with obstructive CAD (T2DM/CAD ≥70% stenosis), and patients with obstructive CAD (CAD ≥70% stenosis).	S5-S8
Figure S2. Malondialdehyde (MDA) modifications of plasma proteins, including apolipoprotein B-100 (ApoB-100) and fibronectin (FINC), and complement C4A/complement C4B (C4A/C4B) were confirmed using immunoprecipitation (IP) and Western blotting (WB).	S8-S9
Figure S3. Calibration curves for five machine learning models for T2DM vs. T2DM/CAD <70% stenosis (A) and T2DM vs. T2DM/CAD ≥70% stenosis (B).	S10-S11
Table S1. Sample size estimation.	S12
Table S2. Hyperparameter search spaces and optimal configurations selected via outer-loop cross-validation across machine learning algorithms.	S13
Table S3. Coverage of ApoB-100, FINC, C4A, and C4B.	S14
Table S4. Feature selection and ranking criteria across four machine learning models for identifying T2DM/CAD progression (<70% vs. ≥70% stenosis).	S14-S17
Table S5. SHAP-based stability evaluation of predictive features across five machine learning models for T2DM vs. T2DM/CAD <70% stenosis and T2DM vs. T2DM/CAD ≥70% stenosis.	S17-S18
Table S6. Performance metrics and hyperparameters for individual outer fold logs in repeated stratified nested cross-validation.	S18-S72
Table S7. Predictive performance and fairness assessment across subgroups using a calibrated CatBoost and LR.	S72-S73
Section 1.1 Using 10% (w/v) trichloroacetic acid (TCA) in an acetone solution to remove plasma albumin.	S73
Section 1.2 In-solution digestion.	S73-S74
Section 1.3 Identification of malondialdehyde (MDA)-modified peptide adducts through nano-liquid chromatography-tandem mass spectroscopy (LC-MS/MS) and PEAKS 7 software.	S74-S76
Section 1.4 Immunoprecipitation (IP) and Western blotting (WB).	S76-S77
Section 1.5 Enzyme-linked immunosorbent assay (ELISA)	S77
Section 1.6 Thiobarbituric acid (TBA)-reactive substance (TBARS) assay.	S78

Section 1.7 Measurement of MDA-modified protein adducts.	S78-S79
Section 1.8 Detection of autoantibody isotypes against BSA, MDA-BSA adduct, unmodified and MDA-modified peptides.	S79-S80
Section 1.9 Feature selection and ranking.	S80
Section 1.10 Model construction and interpretation.	S81-S82
Section 1.11 Hyperparameters for small-sample machine learning models: Random Forest (RF), eXtreme Gradient Boosting (XGBoost), Light Gradient Boosting Machine (LightGBM) Categorical Boosting (CatBoost), and Logistic Regression (LR).	S82-S83
Section 1.12 Fairness analysis using a calibrated Categorical Boosting (CatBoost) and Logistic Regression (LR)	S833-S84

Supplementary Figures and Tables

A

ApoB-100[1662-1683] (T2DM/CAD ≥70% stenosis and CAD ≥70% stenosis)

C(+54.01)SLLVLENELNAELGLSGASMK

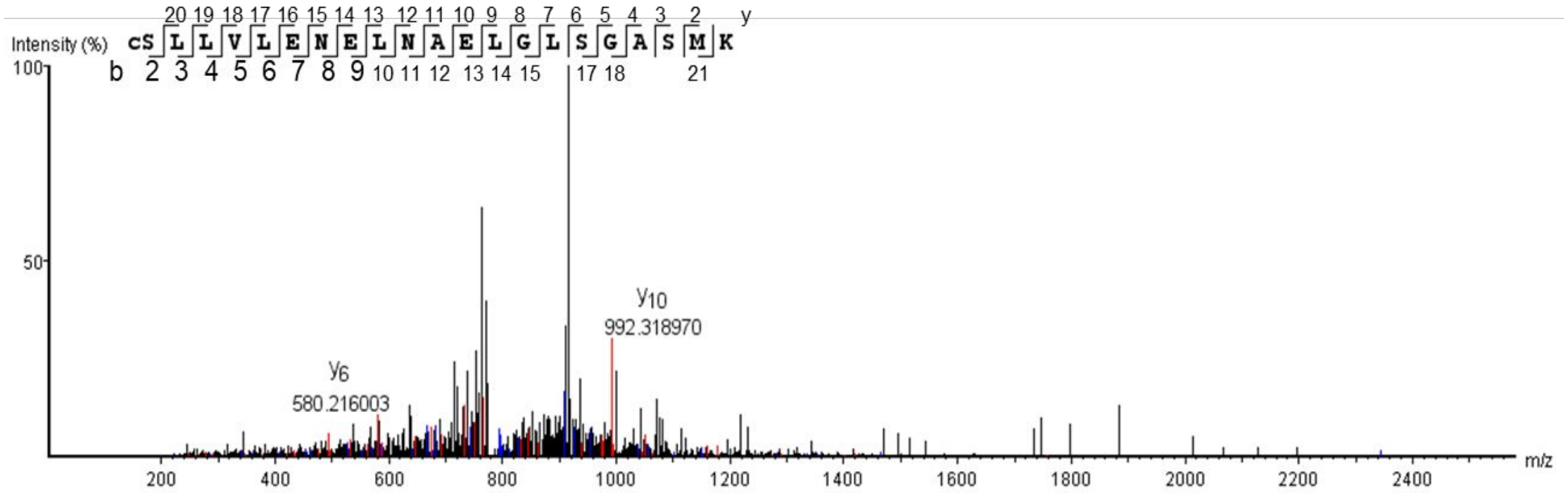

B

ApoB-100[2149-2164] (T2DM)

YRITEN(+54.01)DIQIALDDAK

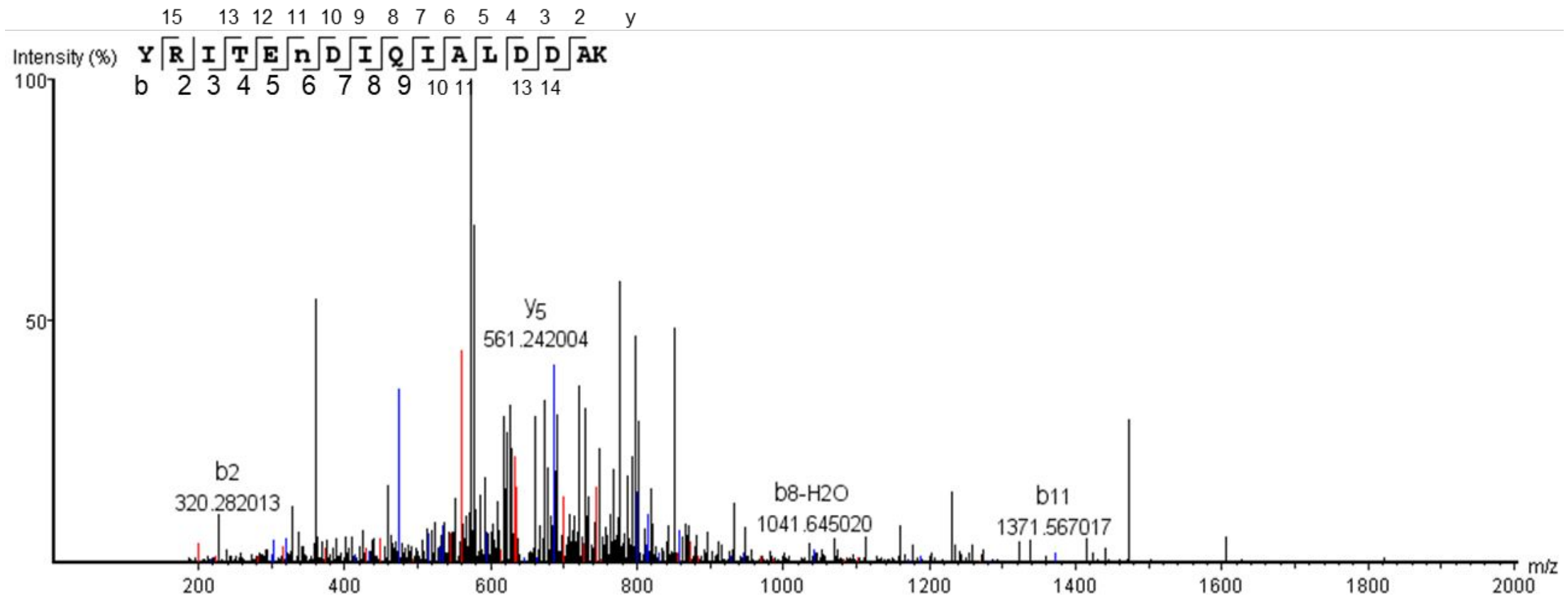

C

FINC[959-984] (T2DM/CAD ≥70% stenosis and CAD ≥70% stenosis)

N(+54.01)TFAEVTGLSPGVTYYFKVFAVSHGR

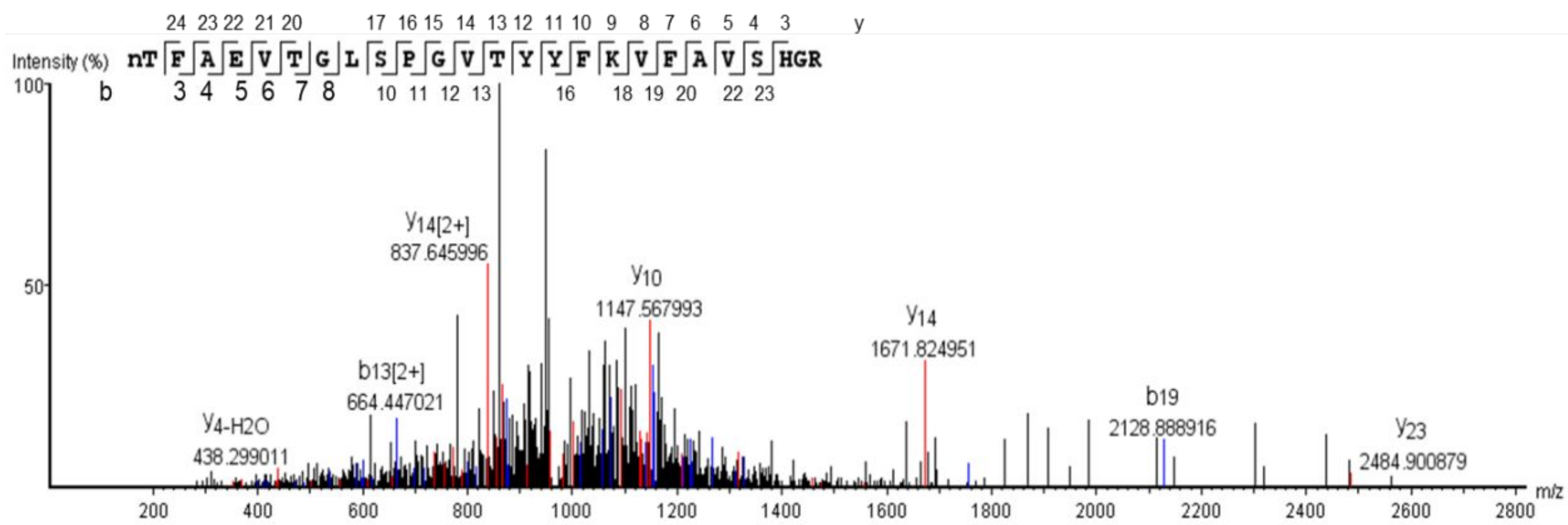

D

FINC$^{4-30}$ (T2DM)

GPGPGLLLLAVQCLGTAVPSTGASKSK(+54.01)

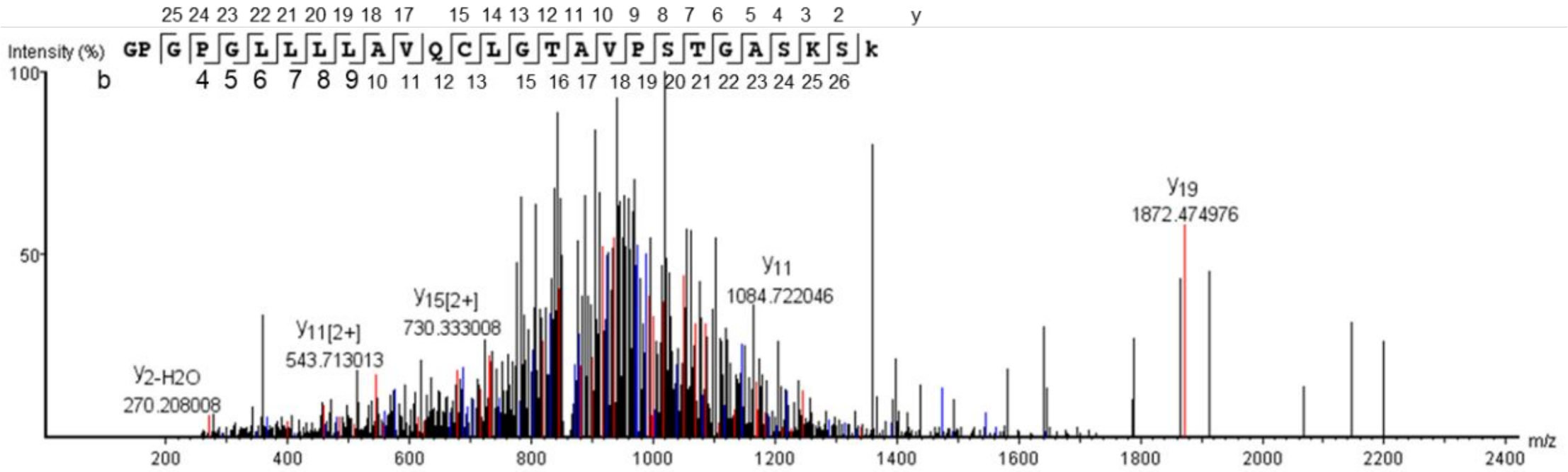

E

C4A$^{167-187}$ (T2DM/CAD ≥70% stenosis and CAD ≥70% stenosis)

M(+54.01)R(+54.01)PSTDTITVMVENSHGLRVR

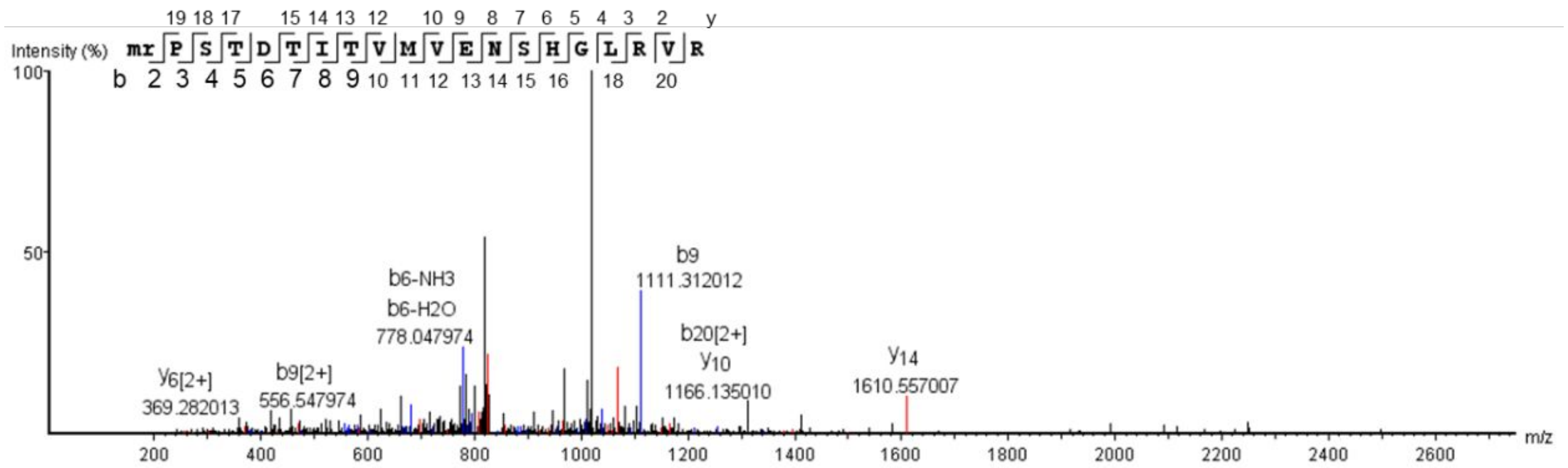

F

C4A$^{305-316}$ (T2DM)

LVNGQSH(+54.01)ISLSK

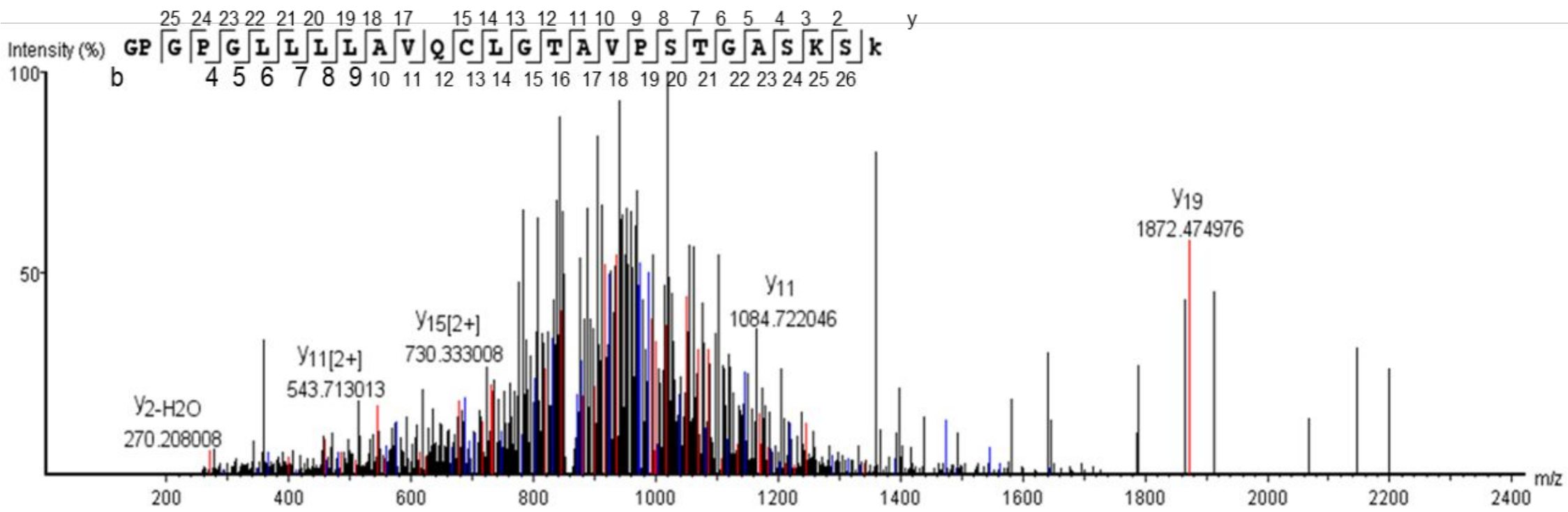

G

C4B$^{167-187}$ (T2DM/CAD ≥70% stenosis and CAD ≥70% stenosis)

M(+54.01)R(+54.01)PSTDTITVMVENSHGLRVR

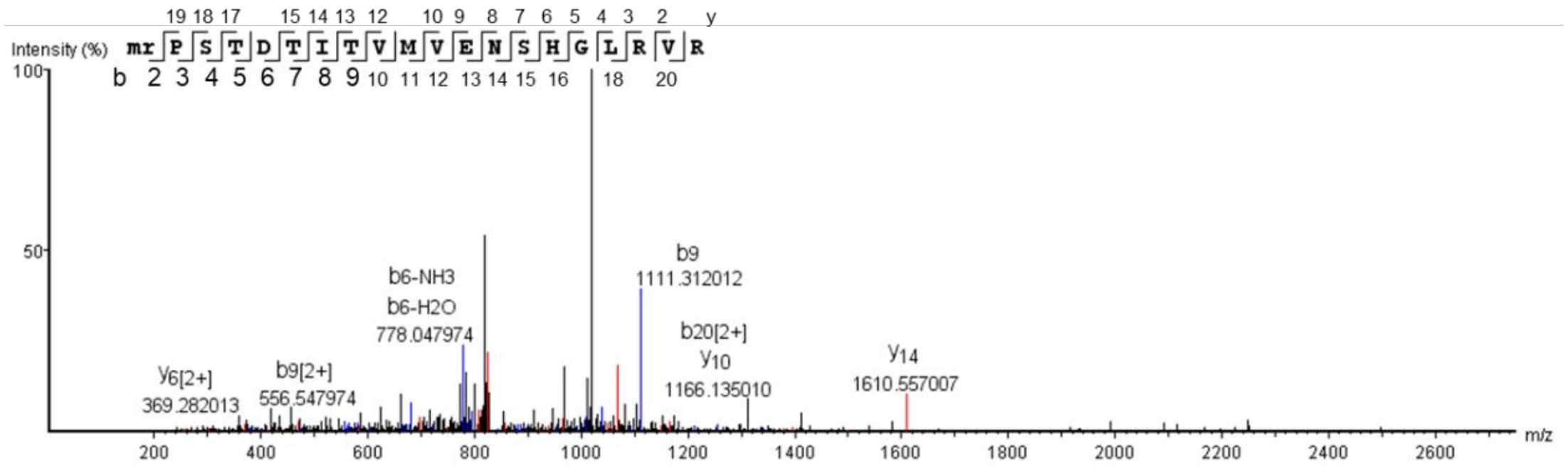

H

C4B[305-316] (T2DM)

LVNGQSH(+54.01)ISLSK

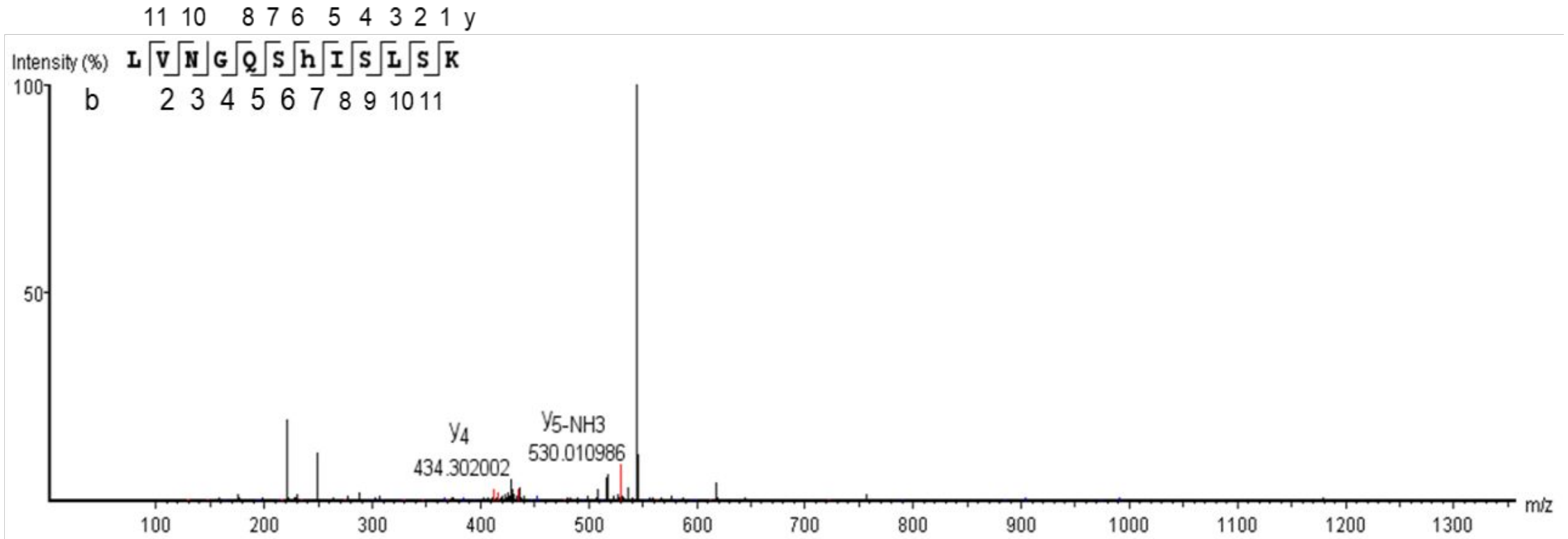

Figure S1. MS/MS spectrum of the malondialdehyde (MDA)-modified peptide (MDA-peptide) adducts in patients with type 2 diabetes mellitus (T2DM), T2DM patients with obstructive CAD (T2DM/CAD ≥70% stenosis), and patients with obstructive CAD (CAD ≥70% stenosis). (A) A representative MS/MS spectrum of the ^{1662}-CSLLVLENELNAELGLSGASMK-^{1683} peptide sequence in apolipoprotein B-100 (ApoB-100); the MDA-modified peptide had a 54.01-Da adduct shift at cysteine (C)1662 in patients with T2DM/CAD ≥70% stenosis and CAD ≥70% stenosis. (B) MS/MS spectrum of the ^{2149}-YRITENDIQIALDDAK-^{2164} peptide sequence in ApoB-100 and the MDA-peptide with the 54.01-Da adduct shift at asparagine (N) 2154 in patients with T2DM. (C) MS/MS spectrum of the ^{959}-NTFAEVTGLSPGVTYYFKVFAVSHGR-^{984} peptide sequence in fibronectin (FINC) and the MDA -peptide with the 54.01-Da adduct shift at N959 in patients with T2DM/CAD

≥70% stenosis and CAD ≥70% stenosis. (D) MS/MS spectrum of the ^{4}-GPGPGLLLLAVQCLGTAV PSTGASKSK-^{30} peptide sequence in FINC and the MDA -peptide with the 54.01-Da adduct shift at lysine (K) 30 in patients with T2DM. (E) MS/MS spectrum of the ^{167}-MRPSTDTITVMVENSHG LRVR-^{187} peptide sequence in complement C4A (C4A) and the MDA-peptide with the 54.01-Da adduct shift in the two residues at methionine (M) 167 and arginine (R) 168 in patients with T2DM/CAD ≥70% stenosis and CAD ≥70% stenosis. (F) MS/MS spectrum of the ^{305}-LVNGQSHISLSK-^{316} peptide sequence in C4A and the MDA-peptide with the 54.01-Da adduct shift at histidine (H) 311 in patients with T2DM. (G) MS/MS spectrum of the ^{167}-MRPSTDTITVMVENSHG LRVR-^{187} peptide sequence in complement C4B (C4B) and the MDA-peptide with the 54.01-Da adduct shift in the two residues M167 and R168 in patients with T2DM/CAD ≥70% stenosis and CAD ≥70% stenosis. (H) MS/MS spectrum of the ^{305}-LVNGQSHISLSK-^{316} peptide sequence in C4B and the MDA-peptide with the 54.01-Da adduct shift at H311 in patients with T2DM.

A

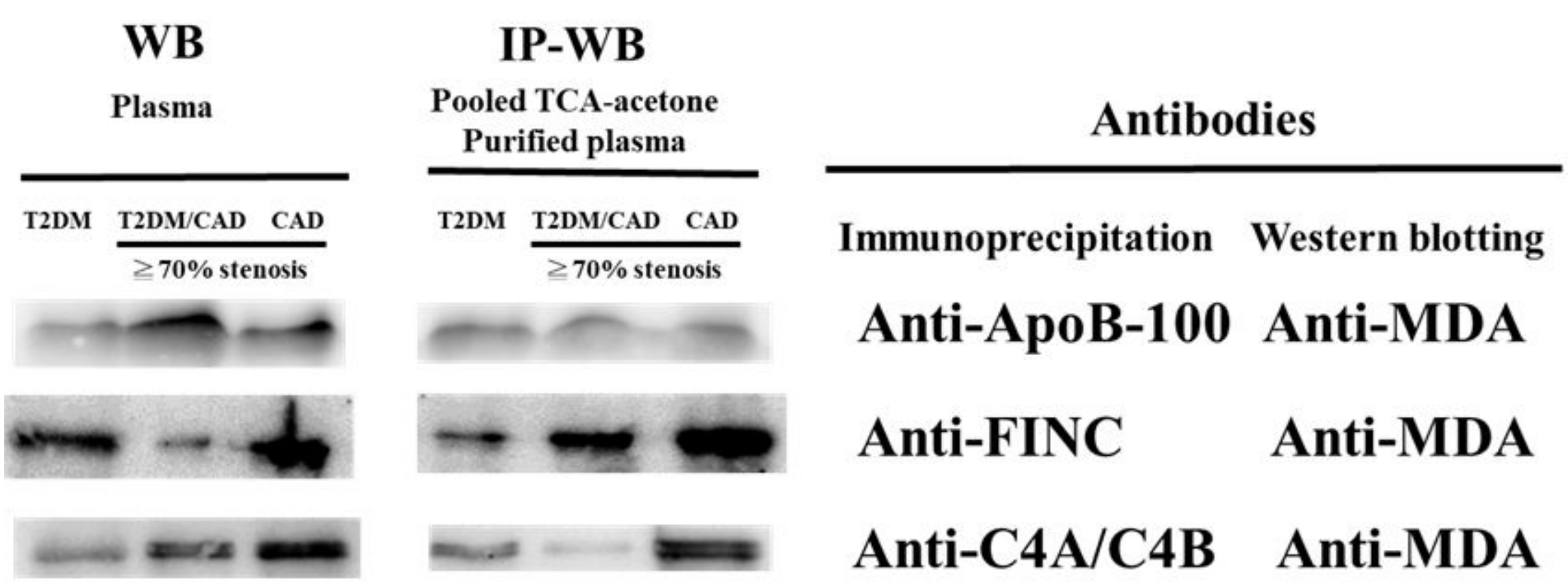

B

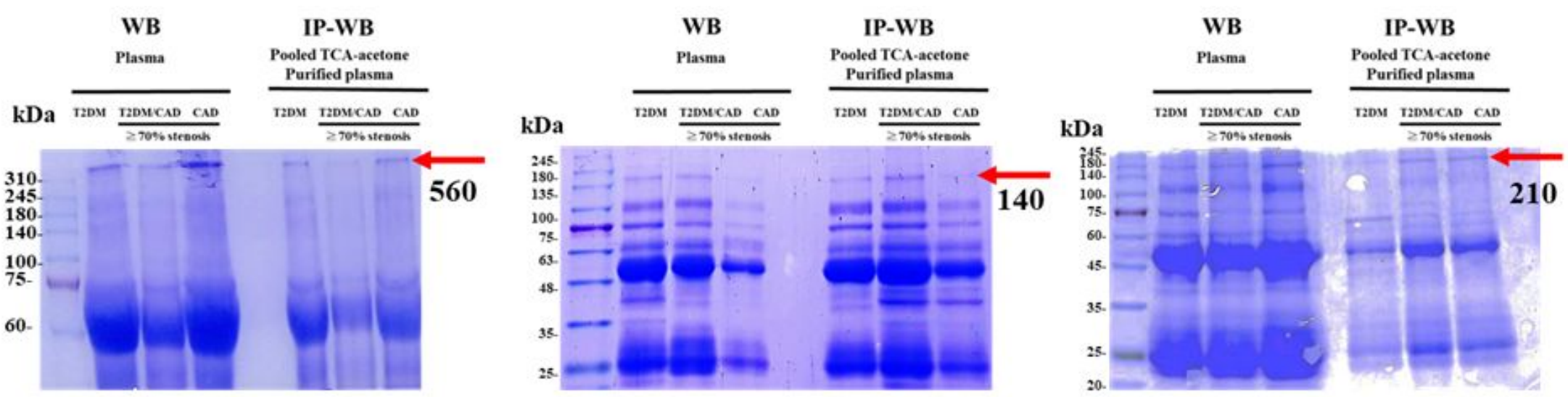

Figure S2. Malondialdehyde (MDA) modifications of plasma proteins, including apolipoprotein B-100 (ApoB-100) and fibronectin (FINC), and complement C4A/complement C4B (C4A/C4B) were confirmed using immunoprecipitation (IP) and Western blotting (WB). (A) ApoB-100, FINC, and C4A/C4B were respectively immunoprecipitated with an anti-ApoB-100, anti-FINC, and anti-C4A/C4B antibody mixed with 200 μg of pooled trichloroacetic acid (TCA)-acetone purified plasma, using 29 pairs of patients with type 2 diabetes mellitus (T2DM), and T2DM patients with obstructive CAD (T2DM/CAD ≥70% stenosis), and patients with obstructive CAD (CAD ≥70% stenosis). Then, 20 μg of immunoprecipitated proteins loaded into an 8% SDS-PAGE gel was subjected to WB using an anti-MDA polyclonal antibody. Next, 200 μg of IgG-depleted plasma samples of a pair of randomly selected individuals was used as controls and simultaneously used for IP-WB (upper panel). (B) A duplicate gel was stained with Coomassie brilliant blue (CBB) as a loading control (bottom panel). The red arrow indicates ApoB-100 (560 kDa), FINC (140 kDa), or C4A/C4B (210 kDa).

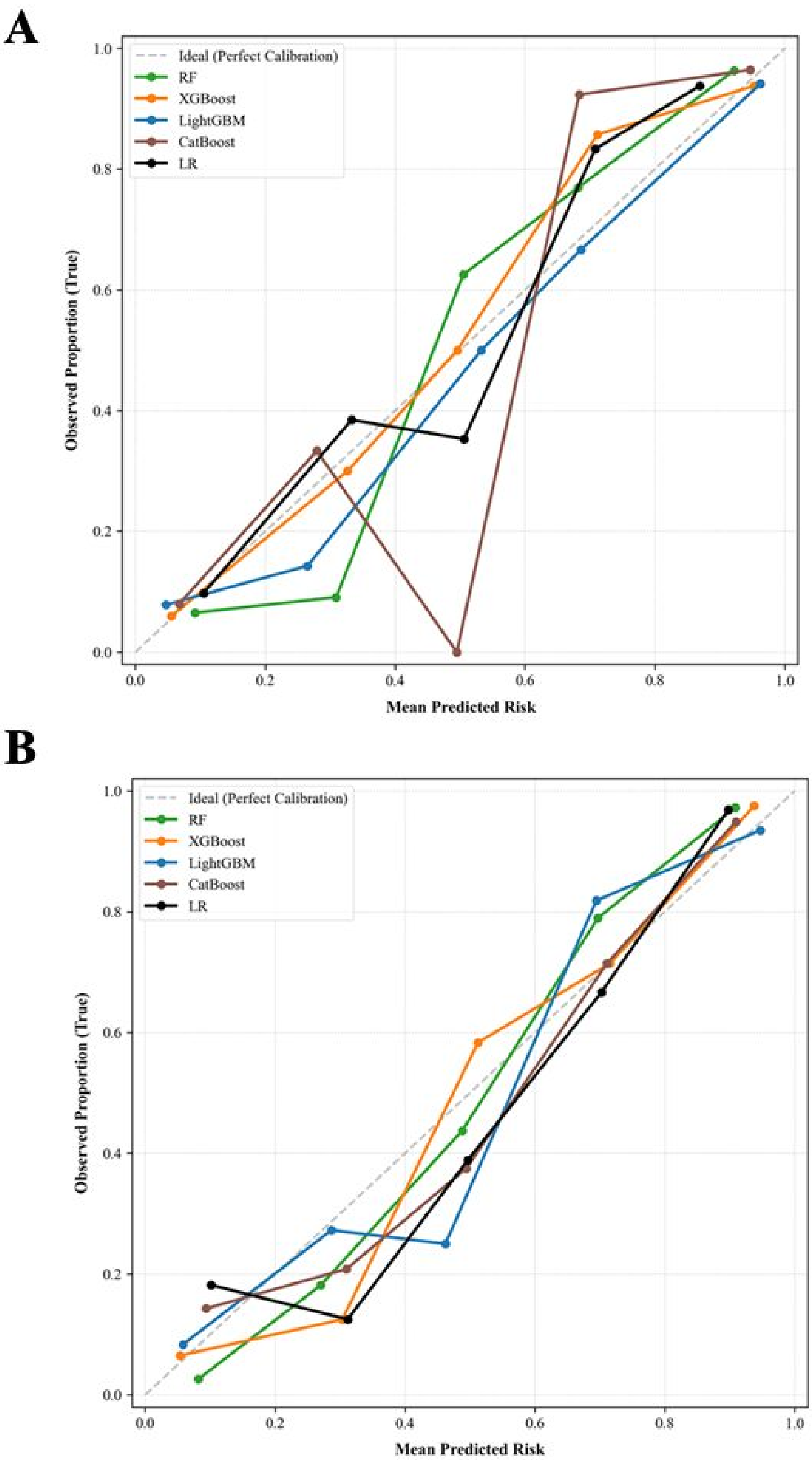

Figure S3. Calibration curves for five machine learning models for T2DM vs. T2DM/CAD <70% stenosis (A) and T2DM vs. T2DM/CAD ≥70% stenosis (B). RF,

Random Forest; XGBoost: eXtreme Gradient Boosting; LightGBM, Light Gradient Boosting Machine; CatBoost, Categorical Boosting; LR, Logistic Regression.

T2DM vs. T2DM/CAD <70% stenosis		
Models	Calibration slope (95% CI)	Calibration Intercept (95% CI)
RF	1.213 (1.026, 1.370)	-0.106 (-0.219, 0.022)
XGBoost	1.053 (0.831, 1.231)	-0.004 (-0.163, 0.165)
LightGBM	1.008 (0.789, 1.196)	-0.037 (-0.182, 0.141)
CatBoost	1.079 (0.850, 1.278)	-0.074 (-0.232, 0.117)
LR	1.123 (0.882, 1.353)	-0.046 (-0.193, 0.110)
T2DM vs. T2DM/CAD ≥70% stenosis		
RF	1.203 (1.028, 1.355)	-0.108 (-0.227, 0.030)
XGBoost	1.098 (0.923, 1.247)	-0.062 (-0.168, 0.070)
LightGBM	1.042 (0.849, 1.217)	-0.038 (-0.190, 0.143)
CatBoost	1.041 (0.837, 1.236)	-0.047 (-0.182, 0.096)
LR	1.061 (0.858, 1.264)	-0.066 (-0.193, 0.072)

Table S1. Sample size estimation

We collected 165 plasma samples, including 60 type 2 diabetes mellitus (T2DM), 45 T2DM patients with non-obstructive CAD (T2DM/CAD <70% stenosis), and 60 T2DM patients with obstructive CAD (T2DM/CAD ≥70% stenosis). We calculated the sample size using G*Power vers. 3.1, based on the mean and standard deviation (SD) of T2DM plus T2DM/CAD <70% stenosis and T2DM/CAD ≥70% stenosis.[1] Sample sizes are analyzed below. Because of the limited sample size, evaluating statistical power was crucial.

Characteristic [a]	MDA	MDA-protein adduct	IgM Anti-APOB-$100^{1662-1683}$	IgM Anti-APOB-$100^{1662-1683}$ MDA	IgG Anti-APOB-$100^{1662-1683}$	IgG Anti-APOB-$100^{1662-1683}$ MDA	IgM Anti-$FINC^{959-984}$	IgM Anti-$FINC^{959-984}$ MDA	IgG Anti-$FINC^{959-984}$	IgG Anti-$FINC^{959-984}$ MDA	IgM Anti-C4A/$C4B^{167-187}$	IgM Anti-C4A/$C4B^{167-187}$ MDA	IgG Anti-C4A/$C4B^{167-187}$	IgG Anti-C4A/$C4B^{167-187}$ MDA
T2DM + T2DM/CAD <70% stenosis, *n* = 105														
Mean	0.258	0.204	629.26	2499.66	388.45	1803.43	768.78	3365.67	299.96	2415.31	470.45	1779.99	334.89	1262.64
SD	0.141	0.050	637.39	2108.21	142.94	681.20	627.55	3035.68	65.05	1295.34	521.07	1746.97	68.27	627.07
T2DM/CAD ≥70% stenosis, n = 60														
Mean	0.384	0.260	569.480	1911.850	403.850	1388.010	738.510	2893.420	294.540	1874.470	467.220	1372.230	371.320	1022.060
SD	0.279	0.104	688.460	1677.450	93.230	585.470	446.750	5033.670	54.710	931.550	393.250	1551.860	80.420	415.710
Sample size estimation														
α = 0.05, β = 0.1														
T2DM + T2DM/CAD <70% stenosis, n	35	24	1356	116	676	27	3565	854	1354	49	224823	182	47	55
T2DM/CAD ≥70% stenosis sample	61	42	2374	204	1184	47	6239	1494	2370	85	393439	318	83	95
α = 0.05, β = 0.2														
T2DM + T2DM/CAD <70% stenosis sample	26	18	1014	87	505	20	2663	638	1012	36	167939	136	36	41
T2DM/CAD ≧70% stenosis, n	46	32	1774	153	885	36	4661	1116	1770	64	293893	238	62	71

a. T2DM, type 2 diabetes mellitus; T2DM/CAD <70% stenosis, T2DM patients with non-obstructive CAD; T2DM/CAD ≥70% stenosis, T2DM patients with obstructive CAD; MDA, Malondialdehyde; MDA-protein adduct, Malondialdehyde-modified protein adduct; ApoB-100, Apolipoprotein B-100; FINC, Fibronectin; C4A/C4B, Complement C4A/complement C4B.

Table S2. Hyperparameter search spaces and optimal configurations selected via outer-loop cross-validation across machine learning algorithms

Model [a]	Hyperparameter search spaces [b]	Outer fold logs (Fold hyperparameter)
**RF**	• 'max_depth': [2, 3]<br>• 'n_estimators': [30, 50]<br>• 'min_samples_leaf': [4]<br>• 'min_samples_split': [10]	• {'max_depth': 2, 'n_estimators': 50}<br>• {'max_depth': 3, 'n_estimators': 50}
**XGBoost**	• 'max_depth': [2, 3]<br>• 'n_estimators': [30, 50]<br>• 'learning_rate': [0.02, 0.05, 0.1]<br>• 'reg_lambda': [15.0, 20.0, 30.0, 60.0]<br>• 'min_child_weight': [3, 5]	• {'max_depth': 2, 'n_estimators': 30, 'learning_rate': 0.05, 'reg_lambda': 20.0, 'min_child_weight': 3}<br>• {'max_depth': 2, 'n_estimators': 30, 'learning_rate': 0.1, 'reg_lambda': 20.0, 'min_child_samples': 20}
**LightGBM**	• 'max_depth': [2, 3]<br>• 'n_estimators': [30, 50]<br>• 'learning_rate': [0.02, 0.05, 0.1]<br>• 'reg_lambda': [15.0, 20.0, 30.0, 60.0]<br>• 'min_child_samples': [4, 6, 20]	• {'max_depth': 2, 'n_estimators': 30, 'learning_rate': 0.1, 'reg_lambda': 20.0, 'min_child_samples': 20}
**CatBoost**	• 'depth': [2, 3]<br>• 'iterations': [150, 200]<br>• 'learning_rate': [0.02, 0.05, 0.1]<br>• 'l2_leaf_reg': [15.0, 20.0, 30.0, 50.0]	• {'iterations': 200, 'depth': 2, 'l2_leaf_reg': 20}
**LR**	• 'penalty': ['l2']<br>• 'C': [0.01, 0.1, 0.5, 1.0, 2.0]	• {'C': 1.0, 'l1_ratio': 0.5, 'penalty': 'l2'}

a. LightGBM, Light Gradient Boosting Machine; CatBoost, Categorical Boosting; XGBoost: eXtreme Gradient Boosting; RF, Random Forest; LR, Logistic Regression.

b. max_depth, maximum depth; n_estimators, number of estimators; 'min_samples_leaf', minimum samples leaf; min_samples_split, minimum samples split; learning_rate, learning rate; reg_lambda, regularization lambda; min_child_weight, minimum child weight; min_child_samples, minimum child samples; l2_leaf_reg, L2 leaf regularization. Hyperparameter tuning was conducted via a grid search within a nested cross-validation framework, protecting against data leakage and ensuring reliable generalization. Logs from the outer folds capture the parameters most consistently identified as optimal across validation folds.

Table S3. Coverage of ApoB-100, FINC, C4A, and C4B

Accession [a]	-10IgP	Coverage (%)
P04114|APOB_HUMAN	22.77	58.08
P04114|APOB_HUMAN	16.69	47.78
P02751|FINC_HUMAN	17.62	68.64
P02751|FINC_HUMAN	27.92	57.75
P0C0L5|CO4A_HUMAN	21.46	37.54
P0C0L5|CO4A_HUMAN	15.86	26.91
P0C0L5|CO4B_HUMAN	21.46	37.54
P0C0L5|CO4B_HUMAN	15.86	26.91

a. ApoB-100, apolipoprotein B100; FINC, fibronectin; C4A, complement C4A; C4B, complement C4B.

Table S4. Feature selection and ranking criteria across four machine learning models for identifying T2DM/CAD progression (<70% vs. ≥70% stenosis)

**Model [a]**	**Feature [b]**	**SHAP-Cov [c] (95%CI)**	**AUC [d] (95% CI)**	**Brier score (95% CI)**	**VIF [e]**	**Impact rank [f]/ Status**
LightGBM	IgG anti-ApoB-100$^{1662-1683}$ MDA	Q1:(0.594, 1.931) | Q4:(-2.179, -0.406)	0.803 (0.776, 0.833)	0.170 (0.156, 0.184)	1	1/ Pass
CatBoost	IgG anti-ApoB-100$^{1662-1683}$ MDA	Q1:(0.588, 1.881) | Q4:(-1.906, -0.461)	0.836 (0.804, 0.863)	0.154 (0.142, 0.168)	1	1/ Pass
XGBoost	IgG anti-ApoB-100$^{1662-1683}$ MDA	Q1:(0.341, 2.243) | Q4:(-2.778, -0.837)	0.875 (0.832, 0.916)	0.125 (0.096, 0.154)	1	1/ Pass
RF	IgG anti-ApoB-100$^{1662-1683}$ MDA	Q1:(0.130, 0.320) | Q4:(-0.305, -0.076)	0.876 (0.839, 0.913)	0.122 (0.099, 0.148)	1	1/ Pass
LightGBM	IgG anti-FINC$^{959-984}$ MDA	Q1:(-0.681, 0.721) | Q4:(-1.824, -0.419)	0.790 (0.758, 0.817)	0.174 (0.161, 0.186)	3.62	2/ Fail
CatBoost	IgG anti-FINC$^{959-984}$ MDA	Q1:(-0.556, 0.525) | Q4:(-1.573, -0.329)	0.824 (0.796, 0.853)	0.158 (0.150, 0.168)	3.62	2/ Fail
XGBoost	IgG anti-FINC$^{959-984}$ MDA	Q1:(-1.235, 0.481) | Q4:(-2.280, -0.639)	0.859 (0.813, 0.905)	0.131 (0.104, 0.160)	3.98	3/ Fail
RF	IgG anti-FINC$^{959-984}$ MDA	Q1:(-0.140, 0.120) | Q4:(-0.287, -0.064)	0.863 (0.819, 0.913)	0.130 (0.102, 0.167)	4.17	3/ Fail
LightGBM	MDA	Q1:(-3.843, -1.487) | Q4:(0.463, 2.210)	0.780 (0.732, 0.815)	0.184 (0.172, 0.199)	2.35	3/ Pass
CatBoost	MDA	Q1:(-2.899, -2.089) | Q4:(0.538, 1.794)	0.820 (0.787, 0.849)	0.167 (0.157, 0.179)	2.35	3/ Pass
XGBoost	MDA	Q1:(-3.579, -2.540) | Q4:(-0.075, 1.532)	0.859 (0.812, 0.898)	0.144 (0.119,	2.14	2/ Fail

				0.174)		
RF	MDA	Q1:(-0.322, -0.233) | Q4:(0.013, 0.267)	0.850 (0.812, 0.896)	0.150 (0.119, 0.176)	2.44	5/ Pass
LightGBM	IgM antFINC959-984	Q1:(0.212, 1.392) | Q4:(0.467, 1.734)	0.762 (0.715, 0.803)	0.188 (0.171, 0.204)	2.43	4/ Pass
CatBoost	IgM anti-FINC959-984	Q1:(0.086, 1.291) | Q4:(0.360, 1.481)	0.815 (0.775, 0.845)	0.167 (0.158, 0.180)	2.43	4/ Pass
XGBoost	IgM anti-FINC959-984	Q1:(-0.084, 1.416) | Q4:(0.350, 2.022)	0.854 (0.800, 0.907)	0.139 (0.109, 0.174)	2.43	4/ Fail
RF	IgM anti-FINC959-984	Q1:(0.012, 0.260) | Q4:(0.054, 0.325)	0.871 (0.822, 0.918)	0.129 (0.103, 0.162)	2.03	2/ Pass
LightGBM	IgG anti-C4A/B167-187 MDA	Q1:(-0.243, 1.159) | Q4:(-1.253, 0.158)	0.759 (0.715, 0.797)	0.188 (0.172, 0.204)	6.53	5/ Fail
CatBoost	IgG anti-C4A/B167-187 MDA	Q1:(0.106, 1.248) | Q4:(-1.382, -0.206)	0.808 (0.774, 0.841)	0.169 (0.158, 0.180)	6.53	5/ Pass
XGBoost	IgG anti-C4A/B167-187 MDA	Q1:(-0.479, 1.108) | Q4:(-1.616, -0.179)	0.849 (0.792, 0.906)	0.138 (0.105, 0.168)	6.53	5/ Fail
RF	IgG anti-C4A/B167-187 MDA	Q1:(0.011, 0.210) | Q4:(-0.224, 0.025)	0.856 (0.804, 0.919)	0.137 (0.103, 0.167)	6.52	4/ Fail
LightGBM	MDA-protein adduct	Q1:(-1.509, -0.320) | Q4:(0.639, 1.655)	0.757 (0.711, 0.793)	0.187 (0.172, 0.205)	5.28	6/ Pass
CatBoost	MDA-protein adduct	Q1:(-1.804, -0.480) | Q4:(0.654, 1.824)	0.799 (0.756, 0.828)	0.170 (0.161, 0.184)	5.28	6/ Pass
XGBoost	MDA-protein adduct	Q1:(-2.378, -0.824) | Q4:(0.373, 1.892)	0.840 (0.796, 0.886)	0.143 (0.118, 0.171)	5.28	6/ Pass
RF	MDA-protein adduct	Q1:(-0.273, -0.055) | Q4:(0.090, 0.307)	0.837 (0.792, 0.880)	0.148 (0.120, 0.177)	8.9	8/ Pass
LightGBM	IgG anti-C4A/B167-187	Q1:(-1.878, -0.393) | Q4:(0.272, 1.531)	0.734 (0.690, 0.770)	0.198 (0.183, 0.211)	13.4	8/ Pass
CatBoost	IgG anti-C4A/B167-187	Q1:(-1.903, -0.483) | Q4:(0.279, 1.351)	0.775 (0.740, 0.813)	0.184 (0.172, 0.196)	13.4	7/ Pass
XGBoost	IgG anti-C4A/B167-187	Q1:(-2.505, -0.829) | Q4:(-0.120, 1.198)	0.839 (0.789, 0.883)	0.150 (0.123, 0.182)	13.4	7/ Fail
RF	IgG anti-C4A/B167-187	Q1:(-0.269, -0.059) | Q4:(0.012, 0.242)	0.838 (0.795, 0.888)	0.150 (0.119, 0.182)	8.39	7/ Pass
LightGBM	IgM anti-C4A/B167-187 MDA	Q1:(0.290, 1.362) | Q4:(-1.184, 0.041)	0.723 (0.684, 0.779)	0.200 (0.183, 0.215)	2.66	9/ Fail
CatBoost	IgM anti-C4A/B167-187 MDA	Q1:(0.323, 1.342) | Q4:(-1.267, -0.016)	0.766 (0.732, 0.801)	0.187 (0.176, 0.198)	2.60	8/ Pass
XGBoost	IgM anti-C4A/B167-187 MDA	Q1:(-0.064, 1.652) | Q4:(-2.028, -0.332)	0.833 (0.768, 0.889)	0.148 (0.121, 0.181)	2.60	8/ Fail
RF	IgM anti-C4A/B167-187 MDA	Q1:(0.045, 0.296) | Q4:(-0.241, 0.021)	0.839 (0.787, 0.895)	0.145 (0.111,	2.47	6/ Fail

				0.177)		
LightGBM	IgM anti-FINC$^{959-984}$ MDA	Q1:(0.134, 1.555) | Q4:(-0.879, 0.448)	0.697 (0.640, 0.742)	0.207 (0.193, 0.226)	2.00	13/ Fail
CatBoost	IgM anti-FINC$^{959-984}$ MDA	Q1:(0.161, 1.307) | Q4:(-1.049, 0.181)	0.766 (0.731, 0.803)	0.187 (0.177, 0.200)	1.99	9 Fail
XGBoost	IgM anti-FINC$^{959-984}$ MDA	Q1:(-0.102, 1.506) | Q4:(-1.473, 0.159)	0.820 (0.770, 0.874)	0.158 (0.128, 0.189)	1.99	9 Fail
RF	IgM anti-FINC$^{959-984}$ MDA	Q1:(0.052, 0.302) | Q4:(-0.156, 0.038)	0.832 (0.780, 0.884)	0.151 (0.119, 0.185)	2.01	10 Fail
LightGBM	IgG anti-ApoB-100$^{1662-1683}$	Q1:(-1.423, -0.159) | Q4:(-0.159, 1.069)	0.691 (0.639, 0.745)	0.209 (0.193, 0.227)	12.1	16/ Fail
CatBoost	IgG anti-ApoB-100$^{1662-1683}$	Q1:(-1.538, -0.293) | Q4:(-0.463, 0.872)	0.746 (0.701, 0.796)	0.194 (0.182, 0.206)	11.9	13/ Fail
XGBoost	IgG anti-ApoB-100$^{1662-1683}$	Q1:(-2.248, -0.540) | Q4:(-0.512, 0.782)	0.817 (0.759, 0.874)	0.155 (0.127, 0.191)	11.8	12/ Fail
RF	IgG anti-ApoB-100$^{1662-1683}$	Q1:(-0.272, -0.024) | Q4:(-0.063, 0.148)	0.831 (0.772, 0.879)	0.149 (0.117, 0.190)	11.5	11/ Fail
LightGBM	IgM anti-ApoB-100$^{1662-1683}$	Q1:(0.042, 1.255) | Q4:(-1.043, 0.281)	0.685 (0.640, 0.725)	0.210 (0.198, 0.231)	2.42	18/ Fail
CatBoost	IgM anti-ApoB-100$^{1662-1683}$	Q1:(-0.071, 1.242) | Q4:(-1.055, 0.211)	0.739 (0.685, 0.782)	0.196 (0.185, 0.212)	2.05	14/ Fail
XGBoost	IgM anti-ApoB-100$^{1662-1683}$	Q1:(-0.212, 1.643) | Q4:(-1.985, -0.357)	0.805 (0.764, 0.853)	0.167 (0.142, 0.192)	2.16	14/ Fail
RF	IgM anti-ApoB-100$^{1662-1683}$	Q1:(0.005, 0.299) | Q4:(-0.183, 0.033)	0.813 (0.771, 0.863)	0.164 (0.128, 0.195)	2.13	13/ Fail
LightGBM	IgM anti-ApoB-100$^{1662-1683}$ MDA	Q1:(-0.050, 1.192) | Q4:(-1.313, -0.163)	0.691 (0.648, 0.737)	0.210 (0.198, 0.225)	6.48	15/ Fail
CatBoost	IgM anti-ApoB-100$^{1662-1683}$ MDA	Q1:(-0.023, 1.095) | Q4:(-1.689, -0.334)	0.739 (0.698, 0.781)	0.197 (0.187, 0.209)	6.47	15/ Fail
XGBoost	IgM anti-ApoB-100$^{1662-1683}$ MDA	Q1:(-0.323, 1.186) | Q4:(-2.460, -0.709)	0.810 (0.755, 0.862)	0.162 (0.135, 0.194)	6.73	13/ Fail
RF	IgM anti-ApoB-100$^{1662-1683}$ MDA	Q1:(-0.039, 0.202) | Q4:(-0.270, -0.038)	0.810 (0.752, 0.859)	0.164 (0.132, 0.199)	6.82	14/ Fail
LightGBM	CRI II	Q1:(0.262, 1.383) | Q4:(-1.437, -0.255)	0.744 (0.708, 0.770)	0.192 (0.183, 0.203)	5.18	7/ Pass
CatBoost	CRI II	Q1:(0.414, 1.462) | Q4:(-1.597, -0.459)	0.765 (0.729, 0.794)	0.185 (0.175, 0.197)	5.29	10/ Pass
XGBoost	CRI II	Q1:(-0.060, 1.499) | Q4:(-2.375, -0.765)	0.770 (0.724, 0.799)	0.187 (0.172, 0.209)	5.64	15/ Fail
RF	CRI II	Q1:(0.062, 0.331) | Q4:(-0.292, -0.070)	0.775 (0.734, 0.806)	0.186 (0.170, 0.209)	5.64	15/ Pass
LightGBM	PCr	Q1:(-0.846, 0.238) |	0.714	0.198	3.58	10/

		Q4:(0.163, 1.261)	(0.660, 0.742)	(0.187, 0.210)		Fail
CatBoost	PCr	Q1:(-1.099, -0.009) | Q4:(0.233, 1.211)	0.730 (0.681, 0.760)	0.186 (0.178, 0.198)	3.60	16/ Pass
XGBoost	PCr	Q1:(-1.062, 0.216) | Q4:(-0.265, 1.253)	0.727 (0.670, 0.748)	0.191 (0.182, 0.206)	3.63	17/ Fail
RF	PCr	Q1:(-0.186, 0.038) | Q4:(0.045, 0.210)	0.739 (0.684, 0.764)	0.187 (0.178, 0.202)	3.63	16/ Fail
LightGBM	CRI I	Q1:(0.015, 1.153) | Q4:(-1.434, 0.058)	0.708 (0.664, 0.734)	0.204 (0.197, 0.215)	23.1	12/ Fail
CatBoost	CRI I	Q1:(-0.080, 1.211) | Q4:(-1.318, -0.001)	0.729 (0.694, 0.760)	0.199 (0.188, 0.213)	26.5	17/ Fail
XGBoost	CRI I	Q1:(-0.676, 1.006) | Q4:(-2.092, 0.001)	0.729 (0.683, 0.762)	0.205 (0.192, 0.226)	27.0	16/ Fail
RF	CRI I	Q1:(-0.044, 0.198) | Q4:(-0.194, 0.027)	0.733 (0.693, 0.769)	0.207 (0.188, 0.228)	27.0	17/ Fail
LightGBM	Age	Q1:(-1.467, -0.220) | Q4:(-0.316, 0.829)	0.687 (0.635, 0.721)	0.210 (0.201, 0.223)	35.6	17/ Fail
CatBoost	Age	Q1:(-1.447, -0.313) | Q4:(-0.223, 0.828)	0.718 (0.675, 0.749)	0.202 (0.193, 0.214)	34.6	18/ Fail
XGBoost	Age	Q1:(-2.346, -0.531) | Q4:(-0.959, 0.625)	0.708 (0.672, 0.743)	0.215 (0.201, 0.230)	35.8	18/ Fail
RF	Age	Q1:(-0.276, -0.044) | Q4:(-0.075, 0.160)	0.724 (0.678, 0.755)	0.213 (0.196, 0.231)	35.8	18/ Fail
LightGBM	TyG Index	Q1:(-0.695, 0.267) | Q4:(-0.508, 0.552)	0.630 (0.589, 0.664)	0.224 (0.216, 0.236)	10.4	19/ Fail
CatBoost	TyG Index	Q1:(-0.888, 0.286) | Q4:(-0.671, 0.380)	0.663 (0.633, 0.693)	0.216 (0.208, 0.227)	9.9	19/ Fail
XGBoost	TyG Index	Q1:(-1.499, 0.262) | Q4:(-0.902, 0.424)	0.656 (0.623, 0.689)	0.226 (0.215, 0.239)	10.8	19/ Fail
RF	TyG Index	Q1:(-0.169, 0.040) | Q4:(-0.101, 0.116)	0.663 (0.628, 0.693)	0.227 (0.214, 0.245)	10.8	19/ Fail
LightGBM	HbA1c HDL Ratio	Q1:(-1.028, 0.113) | Q4:(-0.062, 0.519)	0.545 (0.444, 0.562)	0.230 (0.228, 0.235)	12.0	20/ Fail
CatBoost	HbA1c HDL Ratio	Q1:(-0.771, 0.140) | Q4:(-0.047, 0.383)	0.570 (0.484, 0.587)	0.227 (0.224, 0.234)	11.9	20/ Fail
XGBoost	HbA1c HDL Ratio	Q1:(-1.052, 0.037) | Q4:(-0.021, 0.398)	0.554 (0.447, 0.587)	0.230 (0.225, 0.236)	12.1	20/ Fail
RF	HbA1c HDL Ratio	Q1:(-0.187, 0.024) | Q4:(-0.006, 0.103)	0.570 (0.484, 0.587)	0.227 (0.224, 0.234)	12.1	20/ Fail
LightGBM	Sex	Q1:(-0.862, 0.228) | Q4:(-0.104, 0.402)	0.534 (0.451, 0.549)	0.231 (0.229, 0.238)	3.19	21/ Fail

<table>
<tr><th></th><th></th><th></th><th></th><th></th><th></th><th></th></tr>
<tr><td>CatBoost</td><td>Sex</td><td>Q1:(-0.758, 0.146) | Q4:(-0.066, 0.432)</td><td>0.534 (0.451, 0.549)</td><td>0.231 (0.229, 0.238)</td><td>3.13</td><td>21/ Fail</td></tr>
<tr><td>XGBoost</td><td>Sex</td><td>Q1:(-0.733, 0.121) | Q4:(-0.059, 0.324)</td><td>0.534 (0.451, 0.549)</td><td>0.231 (0.229, 0.238)</td><td>3.19</td><td>21/ Fail</td></tr>
<tr><td>RF</td><td>Sex</td><td>Q1:(-0.157, 0.033) | Q4:(-0.015, 0.092)</td><td>0.535 (0.451, 0.549)</td><td>0.231 (0.229, 0.238)</td><td>3.19</td><td>21/ Fail</td></tr>
</table>

a. LightGBM, Light Gradient Boosting Machine; CatBoost, Categorical Boosting; XGBoost: eXtreme Gradient Boosting; RF, Random Forest.

b. MDA, malondialdehyde; MDA-protein adduct, malondialdehyde-modified protein adduct; ApoB-100, apolipoprotein B-100; FINC, fibronectin; C4A/C4B, complement C4A/complement C4B; T2DM, type 2 diabetes mellitus; T2DM/CAD, type 2 diabetes mellitus and coronary artery disease.

c. SHAP-Cov, Shapley additive explanations with coefficient of variation.

d. AUC, the area under the receiver operating characteristic curve.

e. VIF, variance inflation factor.

f. Impact Rank, determined by the mean |SHAP value| across 100 bootstrap iterations.

Table S5. SHAP-based stability evaluation of predictive features across five machine learning models for T2DM vs. T2DM/CAD <70% stenosis and T2DM vs. T2DM/CAD ≥70% stenosis

<table>
<tr><th>Model [a]</th><th>Feature [b]</th><th>Direction</th><th>VIF [c]</th><th>mean |SHAP value| (95% CI)</th><th>SHAP-Cov [d] (95%CI)</th><th>SHAP-CoV</th><th>Threshold</th></tr>
<tr><td colspan="8">T2DM vs. T2DM/CAD <70% stenosis</td></tr>
<tr><td>RF</td><td>IgG anti-ApoB-100$^{1662-1683}$ MDA</td><td>Negative (-)</td><td>5.32</td><td>0.099 (0.081, 0.150)</td><td>Q1:(0.165, 0.472) | Q4:(-0.423, -0.206)</td><td>1.144</td><td>1.5</td></tr>
<tr><td>RF</td><td>MDA-protein adduct</td><td>Positive (+)</td><td>13.26</td><td>0.148 (0.110, 0.195)</td><td>Q1:(-0.437, -0.306) | Q4:(0.295, 0.532)</td><td>1.147</td><td>1.5</td></tr>
<tr><td>RF</td><td>IgG anti-C4A/B$^{167-187}$</td><td>Positive (+)</td><td>16.43</td><td>0.240 (0.179, 0.278)</td><td>Q1:(-0.429, -0.286) | Q4:(0.435, 0.579)</td><td>1.097</td><td>1.5</td></tr>
<tr><td>XGBoost</td><td>IgG anti-ApoB-100$^{1662-1683}$ MDA</td><td>Negative (-)</td><td>5.32</td><td>0.730 (0.175, 1.492)</td><td>Q1:(1.017, 3.230) | Q4:(-3.898, -1.695)</td><td>1.147</td><td>1.5</td></tr>
<tr><td>XGBoost</td><td>MDA-protein adduct</td><td>Positive (+)</td><td>13.26</td><td>1.948 (1.192, 2.684)</td><td>Q1:(-4.041, -2.606) | Q4:(1.382, 3.714)</td><td>1.105</td><td>1.5</td></tr>
<tr><td>XGBoost</td><td>IgG anti-C4A/B$^{167-187}$</td><td>Positive (+)</td><td>16.43</td><td>3.353 (2.512, 3.952)</td><td>Q1:(-4.531, -1.939) | Q4:(3.600, 4.618)</td><td>1.059</td><td>1.5</td></tr>
<tr><td>LightGBM</td><td>IgG anti-ApoB-100$^{1662-1683}$ MDA</td><td>Negative (-)</td><td>5.34</td><td>1.472 (1.209, 2.696)</td><td>Q1:(0.910, 2.540) | Q4:(-2.772, -0.963)</td><td>1.254</td><td>1.5</td></tr>
<tr><td>LightGBM</td><td>IgM anti-APOB-100$^{1662-1683}$</td><td>Negative (-)</td><td>1.94</td><td>1.192 (0.654, 1.992)</td><td>Q1:(0.191, 1.738) | Q4:(-0.890, 0.610)</td><td>1.105</td><td>1.5</td></tr>
<tr><td>LightGBM</td><td>IgG anti-C4A/B$^{167-187}$</td><td>Positive (+)</td><td>5.87</td><td>3.013 (2.485, 4.809)</td><td>Q1:(-3.933, -1.241) | Q4:(2.714, 5.003)</td><td>1.084</td><td>1.5</td></tr>
<tr><td>CatBoost</td><td>IgG anti-ApoB-100$^{1662-1683}$ MDA</td><td>Negative (-)</td><td>5.32</td><td>0.865 (0.589, 1.295)</td><td>Q1:(0.962, 2.885) | Q4:(-3.385, -1.020)</td><td>1.160</td><td>1.5</td></tr>
</table>

CatBoost	MDA-protein adduct	Positive (+)	13.26	1.205 (0.877, 1.694)	Q1:(-3.754, -1.899) | Q4:(1.521, 3.826)	1.138	1.5
CatBoost	IgG anti-C4A/B$^{167-187}$	Positive (+)	16.43	2.197 (1.794, 2.850)	Q1:(-4.094, -1.653) | Q4:(3.034, 5.181)	1.092	1.5
LR	IgG anti-ApoB-100$^{1662-1683}$ MDA	Negative (-)	6.55	1.522 (1.118, 2.439)	Q1:(0.072, 0.103) | Q4:(-0.042, -0.030)	1.212	1.5
LR	IgG anti-C4A/B$^{167-187}$	Positive (+)	6.20	0.727 (0.502, 1.145)	Q1:(-0.016, 0.003) | Q4:(-0.003, 0.015)	1.273	1.5
LR	CRI II	Negative (-)	5.11	0.009 (0.000, 0.016)	Q1:(0.109, 0.269) | Q4:(-0.386, -0.196)	1.468	1.5
T2DM vs. T2DM/CAD ≥70% Stenosis							
RF	IgG anti-ApoB-100$^{1662-1683}$ MDA	Negative (-)	4.70	0.155 (0.121, 0.206)	Q1:(0.269, 0.470) | Q4:(-0.464, -0.248)	1.069	1.5
RF	IgM anti-FINC$^{959-984}$	Positive (+)	3.37	0.145 (0.111, 0.182)	Q1:(0.051, 0.340) | Q4:(0.323, 0.499)	1.173	1.5
RF	IgG anti-C4A/B$^{167-187}$	Positive (+)	6.13	0.210 (0.158, 0.246)	Q1:(-0.431, -0.170) | Q4:(0.402, 0.507)	1.116	1.5
XGBoost	IgG anti-ApoB-100$^{1662-1683}$ MDA	Negative (-)	4.70	1.637 (0.842, 2.640)	Q1:(1.952, 4.173) | Q4:(-3.867, -1.449)	1.058	1.5
XGBoost	IgM anti-FINC$^{959-984}$	Positive (+)	3.37	1.858 (1.041, 2.190)	Q1:(0.271, 2.311) | Q4:(2.544, 4.294)	1.124	1.5
XGBoost	IgG anti-C4A/B$^{167-187}$	Positive (+)	6.13	2.551 (1.719, 3.507)	Q1:(-3.420, -1.317) | Q4:(3.247, 4.422)	1.115	1.5
LightGBM	IgG anti-ApoB-100$^{1662-1683}$ MDA	Negative (-)	4.70	1.998 (1.240, 3.071)	Q1:(1.299, 3.176) | Q4:(-3.022, -1.048)	1.066	1.5
LightGBM	IgM anti-FINC$^{959-984}$	Positive (+)	3.37	1.947 (1.347, 3.316)	Q1:(0.123, 1.801) | Q4:(1.300, 4.022)	1.170	1.5
LightGBM	IgG anti-C4A/B$^{167-187}$	Positive (+)	6.13	2.957 (2.164, 4.660)	Q1:(-3.105, -1.026) | Q4:(2.144, 4.187)	1.112	1.5
CatBoost	IgG anti-ApoB-100$^{1662-1683}$ MDA	Negative (-)	4.70	1.267 (0.855, 1.838)	Q1:(1.503, 3.275) | Q4:(-3.307, -1.255)	1.066	1.5
CatBoost	IgM anti-FINC$^{959-984}$	Positive (+)	3.37	1.178 (0.806, 1.779)	Q1:(0.334, 2.080) | Q4:(1.771, 3.616)	1.188	1.5
CatBoost	IgG anti-C4A/B$^{167-187}$	Positive (+)	6.13	1.853 (1.531, 2.650)	Q1:(-3.118, -1.184) | Q4:(2.429, 4.307)	1.111	1.5
LR	IgG anti-ApoB-100$^{1662-1683}$ MDA	Negative (-)	6.35	1.070 (0.768, 1.600)	Q1:(0.025, 0.049) | Q4:(-0.063, -0.032)	1.185	1.5
LR	Age	Positive (+)	6.88	0.457 (0.314, 0.672)	Q1:(-0.108, -0.005) | Q4:(0.011, 0.232)	1.196	1.5
LR	CRI II	Negative (-)	4.66	0.002 (0.001, 0.005)	Q1:(0.196, 0.487) | Q4:(-0.214, -0.091)	1.451	1.5

a. LightGBM, Light Gradient Boosting Machine; CatBoost, Categorical Boosting; XGBoost: eXtreme Gradient Boosting; RF, Random Forest; LR, Logistic Regression.

b. MDA, malondialdehyde; MDA-protein adduct, malondialdehyde-modified protein adduct; ApoB-100, apolipoprotein B-100; FINC, fibronectin; C4A/C4B, complement C4A/complement C4B; T2DM, type 2 diabetes mellitus; T2DM/CAD <70% stenosis, T2DM patients with non-obstructive CAD; T2DM/CAD ≥70% stenosis, T2DM patients with obstructive CAD.

c. VIF, variance inflation factor.

d. SHAP-Cov, Shapley additive explanations with coefficient of variation.

Table S6. Performance metrics and hyperparameters for individual outer fold logs in repeated stratified nested cross-validation

Model [a]	Fold	Subgroup N/Event	Feature [b]	mean |SHAP value| [c]	Fold AUC	Brier Score	Calibration Slop/Intercept	Hyperparameters
T2DM vs. T2DM/CAD <70% Stenosis								
RF	1	21/9	IgG anti-ApoB-100$^{1662-1683}$ MDA	0.097	0.972	0.055	3.273/-1.119	{'max_depth': 3, 'n_estimators': 50}
RF	1	21/9	IgG anti-C4A/B$^{167-187}$	0.251	0.972	0.055	3.273/-1.119	{'max_depth': 3, 'n_estimators': 50}
RF	2	21/9	IgG anti-ApoB-100$^{1662-1683}$ MDA	0.194	0.889	0.132	1.260/-0.021	{'max_depth': 3, 'n_estimators': 50}
RF	2	21/9	IgG anti-C4A/B$^{167-187}$	0.299	0.889	0.132	1.260/-0.021	{'max_depth': 3, 'n_estimators': 50}
RF	3	21/9	IgG anti-ApoB-100$^{1662-1683}$ MDA	0.109	1.000	0.058	39.138/8.542	{'max_depth': 3, 'n_estimators': 50}
RF	3	21/9	IgG anti-C4A/B$^{167-187}$	0.243	1.000	0.058	39.138/8.542	{'max_depth': 3, 'n_estimators': 50}
RF	4	21/9	IgG anti-ApoB-100$^{1662-1683}$ MDA	0.096	0.852	0.147	0.801/-0.229	{'max_depth': 3, 'n_estimators': 50}
RF	4	21/9	IgG anti-C4A/B$^{167-187}$	0.286	0.852	0.147	0.801/-0.229	{'max_depth': 3, 'n_estimators': 50}
RF	5	21/9	IgG anti-ApoB-100$^{1662-1683}$ MDA	0.146	0.778	0.106	0.736/0.502	{'max_depth': 3, 'n_estimators': 50}
RF	5	21/9	IgG anti-C4A/B$^{167-187}$	0.278	0.778	0.106	0.736/0.502	{'max_depth': 3, 'n_estimators': 50}
RF	6	21/9	IgG anti-ApoB-100$^{1662-1683}$ MDA	0.107	0.981	0.057	2.260/-1.575	{'max_depth': 3, 'n_estimators': 50}
RF	6	21/9	IgG anti-C4A/B$^{167-187}$	0.304	0.981	0.057	2.260/-1.575	{'max_depth': 3, 'n_estimators': 50}
RF	7	21/9	IgG anti-ApoB-100$^{1662-1683}$ MDA	0.161	0.972	0.070	2.775/0.704	{'max_depth': 3, 'n_estimators': 50}
RF	7	21/9	IgG anti-C4A/B$^{167-187}$	0.260	0.972	0.070	2.775/0.704	{'max_depth': 3, 'n_estimators': 50}
RF	8	21/9	IgG anti-ApoB-100$^{1662-1683}$ MDA	0.138	0.880	0.124	0.789/0.009	{'max_depth': 3, 'n_estimators': 50}

RF	8	21/9	IgG anti-C4A/B167-187	0.314	0.880	0.124	0.789/0.009	{'max_depth': 3, 'n_estimators': 50}
RF	9	21/9	IgG anti-ApoB-1001662-1683 MDA	0.152	0.926	0.094	1.306/0.322	{'max_depth': 3, 'n_estimators': 50}
RF	9	21/9	IgG anti-C4A/B167-187	0.246	0.926	0.094	1.306/0.322	{'max_depth': 3, 'n_estimators': 50}
RF	10	21/9	IgG anti-ApoB-1001662-1683 MDA	0.119	0.907	0.117	1.003/0.040	{'max_depth': 3, 'n_estimators': 50}
RF	10	21/9	IgG anti-C4A/B167-187	0.277	0.907	0.117	1.003/0.040	{'max_depth': 3, 'n_estimators': 50}
RF	11	21/9	IgG anti-ApoB-1001662-1683 MDA	0.151	0.981	0.060	2.307/-1.328	{'max_depth': 2, 'n_estimators': 50}
RF	11	21/9	IgG anti-C4A/B167-187	0.280	0.981	0.060	2.307/-1.328	{'max_depth': 2, 'n_estimators': 50}
RF	12	21/9	IgG anti-ApoB-1001662-1683 MDA	0.114	0.963	0.085	1.994/0.312	{'max_depth': 3, 'n_estimators': 50}
RF	12	21/9	IgG anti-C4A/B167-187	0.260	0.963	0.085	1.994/0.312	{'max_depth': 3, 'n_estimators': 50}
RF	13	21/9	IgG anti-ApoB-1001662-1683 MDA	0.145	0.954	0.098	1.575/0.340	{'max_depth': 3, 'n_estimators': 50}
RF	13	21/9	IgG anti-C4A/B167-187	0.242	0.954	0.098	1.575/0.340	{'max_depth': 3, 'n_estimators': 50}
RF	14	21/9	IgG anti-ApoB-1001662-1683 MDA	0.116	0.843	0.179	0.531/0.364	{'max_depth': 3, 'n_estimators': 50}
RF	14	21/9	IgG anti-C4A/B167-187	0.330	0.843	0.179	0.531/0.364	{'max_depth': 3, 'n_estimators': 50}
RF	15	21/9	IgG anti-ApoB-1001662-1683 MDA	0.139	0.991	0.057	8.251/-1.825	{'max_depth': 2, 'n_estimators': 50}
RF	15	21/9	IgG anti-C4A/B167-187	0.289	0.991	0.057	8.251/-1.825	{'max_depth': 2, 'n_estimators': 50}
RF	16	21/9	IgG anti-ApoB-1001662-1683 MDA	0.133	1.000	0.036	16.005/-0.844	{'max_depth': 3, 'n_estimators': 50}
RF	16	21/9	CRI I	0.249	1.000	0.036	16.005/-0.844	{'max_depth': 3, 'n_estimators': 50}
RF	17	21/9	IgG anti-ApoB-1001662-1683 MDA	0.177	0.889	0.126	0.347/-0.265	{'max_depth': 2, 'n_estimators': 50}
RF	17	21/9	IgG anti-C4A/B167-187	0.286	0.889	0.126	0.347/-0.265	{'max_depth': 2, 'n_estimators': 50}

RF	18	21/9	IgG anti-ApoB-100[1662-1683] MDA	0.170	0.889	0.164	1.427/1.073	{'max_depth': 3, 'n_estimators': 50}
RF	18	21/9	IgM anti-FINC[959-984]	0.209	0.889	0.164	1.427/1.073	{'max_depth': 3, 'n_estimators': 50}
RF	19	21/9	IgG anti-ApoB-100[1662-1683] MDA	0.114	0.981	0.062	1.706/-0.140	{'max_depth': 3, 'n_estimators': 50}
RF	19	21/9	IgG anti-C4A/B[167-187]	0.277	0.981	0.062	1.706/-0.140	{'max_depth': 3, 'n_estimators': 50}
RF	20	21/9	IgG anti-ApoB-100[1662-1683] MDA	0.142	0.856	0.156	0.311/-0.263	{'max_depth': 2, 'n_estimators': 50}
RF	20	21/9	IgG anti-C4A/B[167-187]	0.306	0.856	0.156	0.311/-0.263	{'max_depth': 2, 'n_estimators': 50}
RF	21	21/9	IgG anti-ApoB-100[1662-1683] MDA	0.126	0.889	0.101	1.005/0.910	{'max_depth': 3, 'n_estimators': 50}
RF	21	21/9	IgG anti-C4A/B[167-187]	0.279	0.889	0.101	1.005/0.910	{'max_depth': 3, 'n_estimators': 50}
RF	22	21/9	IgG anti-ApoB-100[1662-1683] MDA	0.139	0.935	0.117	1.387/-0.680	{'max_depth': 3, 'n_estimators': 50}
RF	22	21/9	IgG anti-C4A/B[167-187]	0.299	0.935	0.117	1.387/-0.680	{'max_depth': 3, 'n_estimators': 50}
RF	23	21/9	IgG anti-ApoB-100[1662-1683] MDA	0.105	0.963	0.086	1.467/-0.618	{'max_depth': 3, 'n_estimators': 50}
RF	23	21/9	IgG anti-C4A/B[167-187]	0.297	0.963	0.086	1.467/-0.618	{'max_depth': 3, 'n_estimators': 50}
RF	24	21/9	IgG anti-ApoB-100[1662-1683] MDA	0.155	0.898	0.108	0.896/0.259	{'max_depth': 3, 'n_estimators': 50}
RF	24	21/9	IgG anti-C4A/B[167-187]	0.290	0.898	0.108	0.896/0.259	{'max_depth': 3, 'n_estimators': 50}
RF	25	21/9	IgG anti-ApoB-100[1662-1683] MDA	0.170	0.981	0.070	2.948/1.174	{'max_depth': 3, 'n_estimators': 50}
RF	25	21/9	IgG anti-C4A/B[167-187]	0.229	0.981	0.070	2.948/1.174	{'max_depth': 3, 'n_estimators': 50}
XGBoost	1	21/9	IgG anti-ApoB-100[1662-1683] MDA	0.608	0.991	0.043	5.810/-0.152	{'max_depth': 2, 'n_estimators': 30, 'learning_rate': 0.05, 'reg_lambda': 20.0, 'min_child_weight': 3}

XGBoost	1	21/9	IgG anti-C4A/B$^{167-187}$	1.737	0.991	0.043	5.810/-0.152	{'max_depth': 2, 'n_estimators': 30, 'learning_rate': 0.05, 'reg_lambda': 20.0, 'min_child_weight': 3}
XGBoost	2	21/9	IgG anti-ApoB-100$^{1662-1683}$ MDA	1.424	0.935	0.118	1.076/-0.138	{'max_depth': 2, 'n_estimators': 30, 'learning_rate': 0.05, 'reg_lambda': 20.0, 'min_child_weight': 3}
XGBoost	2	21/9	IgG anti-C4A/B$^{167-187}$	2.253	0.935	0.118	1.076/-0.138	{'max_depth': 2, 'n_estimators': 30, 'learning_rate': 0.05, 'reg_lambda': 20.0, 'min_child_weight': 3}
XGBoost	3	21/9	IgG anti-ApoB-100$^{1662-1683}$ MDA	0.853	0.991	0.066	9.474/-4.145	{'max_depth': 2, 'n_estimators': 30, 'learning_rate': 0.05, 'reg_lambda': 20.0, 'min_child_weight': 3}
XGBoost	3	21/9	IgG anti-C4A/B$^{167-187}$	1.796	0.991	0.066	9.474/-4.145	{'max_depth': 2, 'n_estimators': 30, 'learning_rate': 0.05, 'reg_lambda': 20.0, 'min_child_weight': 3}
XGBoost	4	21/9	IgG anti-ApoB-100$^{1662-1683}$ MDA	1.123	0.870	0.157	0.501/-0.296	{'max_depth': 2, 'n_estimators': 30, 'learning_rate': 0.1, 'reg_lambda': 20.0, 'min_child_weight': 3}
XGBoost	4	21/9	IgG anti-C4A/B$^{167-187}$	2.850	0.870	0.157	0.501/-0.296	{'max_depth': 2, 'n_estimators': 30, 'learning_rate': 0.1, 'reg_lambda': 20.0, 'min_child_weight': 3}
XGBoost	5	21/9	IgG anti-ApoB-100$^{1662-1683}$ MDA	1.118	0.866	0.095	1.030/1.714	{'max_depth': 2, 'n_estimators': 30, 'learning_rate': 0.05,

								'reg_lambda': 20.0, 'min_child_weight': 3}
XGBoost	5	21/9	IgG anti-C4A/B$^{167-187}$	2.304	0.866	0.095	1.030/1.714	{'max_depth': 2, 'n_estimators': 30, 'learning_rate': 0.05, 'reg_lambda': 20.0, 'min_child_weight': 3}
XGBoost	6	21/9	IgG anti-ApoB-100$^{1662-1683}$ MDA	0.759	0.991	0.049	1.753/-1.459	{'max_depth': 2, 'n_estimators': 30, 'learning_rate': 0.05, 'reg_lambda': 20.0, 'min_child_weight': 3}
XGBoost	6	21/9	IgG anti-C4A/B$^{167-187}$	2.314	0.991	0.049	1.753/-1.459	{'max_depth': 2, 'n_estimators': 30, 'learning_rate': 0.05, 'reg_lambda': 20.0, 'min_child_weight': 3}
XGBoost	7	21/9	IgG anti-ApoB-100$^{1662-1683}$ MDA	1.024	0.954	0.094	1.827/2.058	{'max_depth': 2, 'n_estimators': 30, 'learning_rate': 0.05, 'reg_lambda': 20.0, 'min_child_weight': 3}
XGBoost	7	21/9	IgG anti-C4A/B$^{167-187}$	2.182	0.954	0.094	1.827/2.058	{'max_depth': 2, 'n_estimators': 30, 'learning_rate': 0.05, 'reg_lambda': 20.0, 'min_child_weight': 3}
XGBoost	8	21/9	IgG anti-ApoB-100$^{1662-1683}$ MDA	1.203	0.880	0.127	0.571/-0.112	{'max_depth': 2, 'n_estimators': 30, 'learning_rate': 0.1, 'reg_lambda': 20.0, 'min_child_weight': 3}
XGBoost	8	21/9	IgG anti-C4A/B$^{167-187}$	2.827	0.880	0.127	0.571/-0.112	{'max_depth': 2, 'n_estimators': 30, 'learning_rate': 0.1, 'reg_lambda': 20.0, 'min_child_weight': 3}

XGBoost	9	21/9	IgG anti-ApoB-100$^{1662-1683}$ MDA	1.255	0.963	0.082	0.937/0.425	{'max_depth': 2, 'n_estimators': 30, 'learning_rate': 0.1, 'reg_lambda': 20.0, 'min_child_weight': 3}
XGBoost	9	21/9	IgG anti-C4A/B$^{167-187}$	2.168	0.963	0.082	0.937/0.425	{'max_depth': 2, 'n_estimators': 30, 'learning_rate': 0.1, 'reg_lambda': 20.0, 'min_child_weight': 3}
XGBoost	10	21/9	IgG anti-ApoB-100$^{1662-1683}$ MDA	0.766	0.917	0.123	0.725/-0.339	{'max_depth': 2, 'n_estimators': 30, 'learning_rate': 0.05, 'reg_lambda': 20.0, 'min_child_weight': 3}
XGBoost	10	21/9	IgG anti-C4A/B$^{167-187}$	2.292	0.917	0.123	0.725/-0.339	{'max_depth': 2, 'n_estimators': 30, 'learning_rate': 0.05, 'reg_lambda': 20.0, 'min_child_weight': 3}
XGBoost	11	21/9	IgG anti-ApoB-100$^{1662-1683}$ MDA	0.912	0.991	0.055	1.613/-1.648	{'max_depth': 2, 'n_estimators': 30, 'learning_rate': 0.05, 'reg_lambda': 20.0, 'min_child_weight': 3}
XGBoost	11	21/9	IgG anti-C4A/B$^{167-187}$	2.072	0.991	0.055	1.613/-1.648	{'max_depth': 2, 'n_estimators': 30, 'learning_rate': 0.05, 'reg_lambda': 20.0, 'min_child_weight': 3}
XGBoost	12	21/9	IgG anti-ApoB-100$^{1662-1683}$ MDA	0.962	0.981	0.055	1.904/0.530	{'max_depth': 2, 'n_estimators': 30, 'learning_rate': 0.05, 'reg_lambda': 20.0, 'min_child_weight': 3}
XGBoost	12	21/9	IgG anti-C4A/B$^{167-187}$	1.989	0.981	0.055	1.904/0.530	{'max_depth': 2, 'n_estimators': 30, 'learning_rate': 0.05,

								'reg_lambda': 20.0, 'min_child_weight': 3}
XGBoost	13	21/9	IgG anti-ApoB-100$^{1662-1683}$ MDA	1.372	0.963	0.091	1.257/0.400	{'max_depth': 2, 'n_estimators': 30, 'learning_rate': 0.1, 'reg_lambda': 20.0, 'min_child_weight': 3}
XGBoost	13	21/9	IgG anti-C4A/B$^{167-187}$	2.086	0.963	0.091	1.257/0.400	{'max_depth': 2, 'n_estimators': 30, 'learning_rate': 0.1, 'reg_lambda': 20.0, 'min_child_weight': 3}
XGBoost	14	21/9	IgG anti-ApoB-100$^{1662-1683}$ MDA	1.136	0.870	0.187	0.430/0.484	{'max_depth': 2, 'n_estimators': 30, 'learning_rate': 0.1, 'reg_lambda': 20.0, 'min_child_weight': 3}
XGBoost	14	21/9	IgG anti-C4A/B$^{167-187}$	3.368	0.870	0.187	0.430/0.484	{'max_depth': 2, 'n_estimators': 30, 'learning_rate': 0.1, 'reg_lambda': 20.0, 'min_child_weight': 3}
XGBoost	15	21/9	IgG anti-ApoB-100$^{1662-1683}$ MDA	1.077	0.968	0.081	1.196/-0.378	{'max_depth': 2, 'n_estimators': 30, 'learning_rate': 0.05, 'reg_lambda': 20.0, 'min_child_weight': 3}
XGBoost	15	21/9	IgG anti-C4A/B$^{167-187}$	1.994	0.968	0.081	1.196/-0.378	{'max_depth': 2, 'n_estimators': 30, 'learning_rate': 0.05, 'reg_lambda': 20.0, 'min_child_weight': 3}
XGBoost	16	21/9	IgG anti-ApoB-100$^{1662-1683}$ MDA	0.989	1.000	0.023	14.831/-3.070	{'max_depth': 2, 'n_estimators': 30, 'learning_rate': 0.05, 'reg_lambda': 20.0, 'min_child_weight': 3}

XGBoost	16	21/9	IgG anti-C4A/B$^{167-187}$	2.022	1.000	0.023	14.831/-3.070	{'max_depth': 2, 'n_estimators': 30, 'learning_rate': 0.05, 'reg_lambda': 20.0, 'min_child_weight': 3}
XGBoost	17	21/9	IgG anti-ApoB-100$^{1662-1683}$ MDA	1.183	0.912	0.115	0.784/-0.404	{'max_depth': 2, 'n_estimators': 30, 'learning_rate': 0.05, 'reg_lambda': 20.0, 'min_child_weight': 3}
XGBoost	17	21/9	IgG anti-C4A/B$^{167-187}$	2.120	0.912	0.115	0.784/-0.404	{'max_depth': 2, 'n_estimators': 30, 'learning_rate': 0.05, 'reg_lambda': 20.0, 'min_child_weight': 3}
XGBoost	18	21/9	IgG anti-ApoB-100$^{1662-1683}$ MDA	0.888	1.000	0.099	58.775/79.961	{'max_depth': 2, 'n_estimators': 30, 'learning_rate': 0.05, 'reg_lambda': 20.0, 'min_child_weight': 3}
XGBoost	18	21/9	IgG anti-C4A/B$^{167-187}$	2.017	1.000	0.099	58.775/79.961	{'max_depth': 2, 'n_estimators': 30, 'learning_rate': 0.05, 'reg_lambda': 20.0, 'min_child_weight': 3}
XGBoost	19	21/9	IgG anti-ApoB-100$^{1662-1683}$ MDA	0.750	0.981	0.059	1.167/-0.068	{'max_depth': 2, 'n_estimators': 30, 'learning_rate': 0.05, 'reg_lambda': 20.0, 'min_child_weight': 3}
XGBoost	19	21/9	IgG anti-C4A/B$^{167-187}$	2.172	0.981	0.059	1.167/-0.068	{'max_depth': 2, 'n_estimators': 30, 'learning_rate': 0.05, 'reg_lambda': 20.0, 'min_child_weight': 3}
XGBoost	20	21/9	IgG anti-ApoB-100$^{1662-1683}$ MDA	1.294	0.856	0.137	0.595/-0.253	{'max_depth': 2, 'n_estimators': 30, 'learning_rate': 0.1,

								'reg_lambda': 20.0, 'min_child_weight': 3}
XGBoost	20	21/9	IgG anti-C4A/B$^{167-187}$	2.277	0.856	0.137	0.595/-0.253	{'max_depth': 2, 'n_estimators': 30, 'learning_rate': 0.1, 'reg_lambda': 20.0, 'min_child_weight': 3}
XGBoost	21	21/9	IgG anti-ApoB-100$^{1662-1683}$ MDA	1.260	0.843	0.113	0.829/1.401	{'max_depth': 2, 'n_estimators': 30, 'learning_rate': 0.1, 'reg_lambda': 20.0, 'min_child_weight': 3}
XGBoost	21	21/9	IgG anti-C4A/B$^{167-187}$	2.496	0.843	0.113	0.829/1.401	{'max_depth': 2, 'n_estimators': 30, 'learning_rate': 0.1, 'reg_lambda': 20.0, 'min_child_weight': 3}
XGBoost	22	21/9	IgG anti-ApoB-100$^{1662-1683}$ MDA	0.961	0.935	0.108	0.889/-0.305	{'max_depth': 2, 'n_estimators': 30, 'learning_rate': 0.05, 'reg_lambda': 20.0, 'min_child_weight': 3}
XGBoost	22	21/9	IgG anti-C4A/B$^{167-187}$	1.993	0.935	0.108	0.889/-0.305	{'max_depth': 2, 'n_estimators': 30, 'learning_rate': 0.05, 'reg_lambda': 20.0, 'min_child_weight': 3}
XGBoost	23	21/9	IgG anti-ApoB-100$^{1662-1683}$ MDA	0.702	0.963	0.085	1.050/-0.776	{'max_depth': 2, 'n_estimators': 30, 'learning_rate': 0.05, 'reg_lambda': 20.0, 'min_child_weight': 3}
XGBoost	23	21/9	IgG anti-C4A/B$^{167-187}$	2.353	0.963	0.085	1.050/-0.776	{'max_depth': 2, 'n_estimators': 30, 'learning_rate': 0.05, 'reg_lambda': 20.0, 'min_child_weight': 3}

XGBoost	24	21/9	IgG anti-ApoB-100$^{1662-1683}$ MDA	1.039	0.880	0.106	0.700/0.003	{'max_depth': 2, 'n_estimators': 30, 'learning_rate': 0.05, 'reg_lambda': 20.0, 'min_child_weight': 3}
XGBoost	24	21/9	IgG anti-C4A/B$^{167-187}$	2.323	0.880	0.106	0.700/0.003	{'max_depth': 2, 'n_estimators': 30, 'learning_rate': 0.05, 'reg_lambda': 20.0, 'min_child_weight': 3}
XGBoost	25	21/9	IgG anti-ApoB-100$^{1662-1683}$ MDA	1.038	0.986	0.065	10.412/10.172	{'max_depth': 2, 'n_estimators': 30, 'learning_rate': 0.05, 'reg_lambda': 20.0, 'min_child_weight': 3}
XGBoost	25	21/9	IgG anti-C4A/B$^{167-187}$	1.812	0.986	0.065	10.412/10.172	{'max_depth': 2, 'n_estimators': 30, 'learning_rate': 0.05, 'reg_lambda': 20.0, 'min_child_weight': 3}
LightGBM	1	21/9	IgG anti-ApoB-100$^{1662-1683}$ MDA	1.020	0.972	0.045	1.765/-0.950	{'max_depth': 2, 'n_estimators': 30, 'learning_rate': 0.1, 'reg_lambda': 20.0, 'min_child_samples': 20}
LightGBM	1	21/9	IgG anti-C4A/B$^{167-187}$	2.196	0.972	0.045	1.765/-0.950	{'max_depth': 2, 'n_estimators': 30, 'learning_rate': 0.1, 'reg_lambda': 20.0, 'min_child_samples': 20}
LightGBM	2	21/9	IgG anti-ApoB-100$^{1662-1683}$ MDA	1.613	0.926	0.114	0.983/-0.081	{'max_depth': 2, 'n_estimators': 30, 'learning_rate': 0.1, 'reg_lambda': 20.0, 'min_child_samples': 20}
LightGBM	2	21/9	IgG anti-C4A/B$^{167-187}$	2.516	0.926	0.114	0.983/-0.081	{'max_depth': 2, 'n_estimators': 30, 'learning_rate': 0.1,

								'reg_lambda': 20.0, 'min_child_samples': 20}
LightGBM	3	21/9	IgG anti-ApoB-100$^{1662-1683}$ MDA	1.267	1.000	0.033	8.838/-13.089	{'max_depth': 2, 'n_estimators': 30, 'learning_rate': 0.1, 'reg_lambda': 20.0, 'min_child_samples': 20}
LightGBM	3	21/9	IgG anti-C4A/B$^{167-187}$	2.317	1.000	0.033	8.838/-13.089	{'max_depth': 2, 'n_estimators': 30, 'learning_rate': 0.1, 'reg_lambda': 20.0, 'min_child_samples': 20}
LightGBM	4	21/9	IgG anti-ApoB-100$^{1662-1683}$ MDA	1.432	0.875	0.182	0.485/-0.287	{'max_depth': 2, 'n_estimators': 30, 'learning_rate': 0.1, 'reg_lambda': 20.0, 'min_child_samples': 20}
LightGBM	4	21/9	IgG anti-C4A/B$^{167-187}$	2.995	0.875	0.182	0.485/-0.287	{'max_depth': 2, 'n_estimators': 30, 'learning_rate': 0.1, 'reg_lambda': 20.0, 'min_child_samples': 20}
LightGBM	5	21/9	IgG anti-ApoB-100$^{1662-1683}$ MDA	1.560	0.884	0.094	0.693/0.882	{'max_depth': 2, 'n_estimators': 30, 'learning_rate': 0.1, 'reg_lambda': 20.0, 'min_child_samples': 20}
LightGBM	5	21/9	IgG anti-C4A/B$^{167-187}$	2.635	0.884	0.094	0.693/0.882	{'max_depth': 2, 'n_estimators': 30, 'learning_rate': 0.1, 'reg_lambda': 20.0, 'min_child_samples': 20}
LightGBM	6	21/9	IgG anti-ApoB-100$^{1662-1683}$ MDA	1.298	1.000	0.032	7.286/-9.066	{'max_depth': 2, 'n_estimators': 30, 'learning_rate': 0.1, 'reg_lambda': 20.0, 'min_child_samples': 20}

LightGBM	6	21/9	IgG anti-C4A/B$^{167-187}$	2.685	1.000	0.032	7.286/-9.066	{'max_depth': 2, 'n_estimators': 30, 'learning_rate': 0.1, 'reg_lambda': 20.0, 'min_child_samples': 20}
LightGBM	7	21/9	IgG anti-ApoB-100$^{1662-1683}$ MDA	1.400	0.977	0.070	8.987/6.938	{'max_depth': 2, 'n_estimators': 30, 'learning_rate': 0.1, 'reg_lambda': 20.0, 'min_child_samples': 20}
LightGBM	7	21/9	IgG anti-C4A/B$^{167-187}$	2.207	0.977	0.070	8.987/6.938	{'max_depth': 2, 'n_estimators': 30, 'learning_rate': 0.1, 'reg_lambda': 20.0, 'min_child_samples': 20}
LightGBM	8	21/9	IgG anti-ApoB-100$^{1662-1683}$ MDA	1.531	0.931	0.122	0.609/0.023	{'max_depth': 2, 'n_estimators': 30, 'learning_rate': 0.1, 'reg_lambda': 20.0, 'min_child_samples': 20}
LightGBM	8	21/9	IgG anti-C4A/B$^{167-187}$	2.959	0.931	0.122	0.609/0.023	{'max_depth': 2, 'n_estimators': 30, 'learning_rate': 0.1, 'reg_lambda': 20.0, 'min_child_samples': 20}
LightGBM	9	21/9	IgG anti-ApoB-100$^{1662-1683}$ MDA	1.221	0.981	0.077	0.904/0.137	{'max_depth': 2, 'n_estimators': 30, 'learning_rate': 0.1, 'reg_lambda': 20.0, 'min_child_samples': 20}
LightGBM	9	21/9	IgG anti-C4A/B$^{167-187}$	2.251	0.981	0.077	0.904/0.137	{'max_depth': 2, 'n_estimators': 30, 'learning_rate': 0.1, 'reg_lambda': 20.0, 'min_child_samples': 20}
LightGBM	10	21/9	IgG anti-ApoB-100$^{1662-1683}$ MDA	1.261	0.907	0.120	0.667/-0.435	{'max_depth': 2, 'n_estimators': 30, 'learning_rate': 0.1,

								'reg_lambda': 20.0, 'min_child_samples': 20}
LightGBM	10	21/9	IgG anti-C4A/B$^{167-187}$	2.769	0.907	0.120	0.667/-0.435	{'max_depth': 2, 'n_estimators': 30, 'learning_rate': 0.1, 'reg_lambda': 20.0, 'min_child_samples': 20}
LightGBM	11	21/9	IgG anti-ApoB-100$^{1662-1683}$ MDA	1.254	1.000	0.025	4.316/-5.962	{'max_depth': 2, 'n_estimators': 30, 'learning_rate': 0.1, 'reg_lambda': 20.0, 'min_child_samples': 20}
LightGBM	11	21/9	IgG anti-C4A/B$^{167-187}$	2.289	1.000	0.025	4.316/-5.962	{'max_depth': 2, 'n_estimators': 30, 'learning_rate': 0.1, 'reg_lambda': 20.0, 'min_child_samples': 20}
LightGBM	12	21/9	IgG anti-ApoB-100$^{1662-1683}$ MDA	1.363	0.972	0.071	1.115/0.033	{'max_depth': 2, 'n_estimators': 30, 'learning_rate': 0.1, 'reg_lambda': 20.0, 'min_child_samples': 20}
LightGBM	12	21/9	IgG anti-C4A/B$^{167-187}$	2.371	0.972	0.071	1.115/0.033	{'max_depth': 2, 'n_estimators': 30, 'learning_rate': 0.1, 'reg_lambda': 20.0, 'min_child_samples': 20}
LightGBM	13	21/9	IgG anti-ApoB-100$^{1662-1683}$ MDA	1.080	0.958	0.091	1.016/0.303	{'max_depth': 2, 'n_estimators': 30, 'learning_rate': 0.1, 'reg_lambda': 20.0, 'min_child_samples': 20}
LightGBM	13	21/9	IgG anti-C4A/B$^{167-187}$	2.188	0.958	0.091	1.016/0.303	{'max_depth': 2, 'n_estimators': 30, 'learning_rate': 0.1, 'reg_lambda': 20.0, 'min_child_samples': 20}

LightGBM	14	21/9	IgG anti-ApoB-100$^{1662-1683}$ MDA	1.411	0.875	0.156	0.434/0.455	{'max_depth': 2, 'n_estimators': 30, 'learning_rate': 0.1, 'reg_lambda': 20.0, 'min_child_samples': 20}
LightGBM	14	21/9	IgG anti-C4A/B$^{167-187}$	3.939	0.875	0.156	0.434/0.455	{'max_depth': 2, 'n_estimators': 30, 'learning_rate': 0.1, 'reg_lambda': 20.0, 'min_child_samples': 20}
LightGBM	15	21/9	IgG anti-ApoB-100$^{1662-1683}$ MDA	1.203	0.977	0.063	1.909/-0.591	{'max_depth': 2, 'n_estimators': 30, 'learning_rate': 0.1, 'reg_lambda': 20.0, 'min_child_samples': 20}
LightGBM	15	21/9	IgG anti-C4A/B$^{167-187}$	2.309	0.977	0.063	1.909/-0.591	{'max_depth': 2, 'n_estimators': 30, 'learning_rate': 0.1, 'reg_lambda': 20.0, 'min_child_samples': 20}
LightGBM	16	21/9	IgG anti-APOB-100$^{1662-1683}$ MDA	1.331	1.000	0.021	9.086/3.437	{'max_depth': 2, 'n_estimators': 30, 'learning_rate': 0.1, 'reg_lambda': 20.0, 'min_child_samples': 20}
LightGBM	16	21/9	IgG anti-C4A/B$^{167-187}$	2.232	1.000	0.021	9.086/3.437	{'max_depth': 2, 'n_estimators': 30, 'learning_rate': 0.1, 'reg_lambda': 20.0, 'min_child_samples': 20}
LightGBM	17	21/9	IgG anti-ApoB-100$^{1662-1683}$ MDA	1.620	0.935	0.077	0.892/-0.417	{'max_depth': 2, 'n_estimators': 30, 'learning_rate': 0.1, 'reg_lambda': 20.0, 'min_child_samples': 20}
LightGBM	17	21/9	IgG anti-C4A/B$^{167-187}$	2.366	0.935	0.077	0.892/-0.417	{'max_depth': 2, 'n_estimators': 30, 'learning_rate': 0.1,

								'reg_lambda': 20.0, 'min_child_samples': 20}
LightGBM	18	21/9	IgG anti-ApoB-100$^{1662-1683}$ MDA	1.190	0.972	0.102	2.023/2.746	{'max_depth': 2, 'n_estimators': 30, 'learning_rate': 0.1, 'reg_lambda': 20.0, 'min_child_samples': 20}
LightGBM	18	21/9	IgG anti-C4A/B$^{167-187}$	2.405	0.972	0.102	2.023/2.746	{'max_depth': 2, 'n_estimators': 30, 'learning_rate': 0.1, 'reg_lambda': 20.0, 'min_child_samples': 20}
LightGBM	19	21/9	IgG anti-ApoB-100$^{1662-1683}$ MDA	1.101	0.991	0.046	1.142/0.260	{'max_depth': 2, 'n_estimators': 30, 'learning_rate': 0.1, 'reg_lambda': 20.0, 'min_child_samples': 20}
LightGBM	19	21/9	IgG anti-C4A/B$^{167-187}$	2.597	0.991	0.046	1.142/0.260	{'max_depth': 2, 'n_estimators': 30, 'learning_rate': 0.1, 'reg_lambda': 20.0, 'min_child_samples': 20}
LightGBM	20	21/9	IgG anti-ApoB-100$^{1662-1683}$ MDA	1.139	0.866	0.131	0.595/-0.323	{'max_depth': 2, 'n_estimators': 30, 'learning_rate': 0.1, 'reg_lambda': 20.0, 'min_child_samples': 20}
LightGBM	20	21/9	IgG anti-C4A/B$^{167-187}$	2.604	0.866	0.131	0.595/-0.323	{'max_depth': 2, 'n_estimators': 30, 'learning_rate': 0.1, 'reg_lambda': 20.0, 'min_child_samples': 20}
LightGBM	21	21/9	IgG anti-ApoB-100$^{1662-1683}$ MDA	1.363	0.889	0.103	0.811/1.716	{'max_depth': 2, 'n_estimators': 30, 'learning_rate': 0.1, 'reg_lambda': 20.0, 'min_child_samples': 20}

LightGBM	21	21/9	IgG anti-C4A/B$^{167\text{-}187}$	2.652	0.889	0.103	0.811/1.716	{'max_depth': 2, 'n_estimators': 30, 'learning_rate': 0.1, 'reg_lambda': 20.0, 'min_child_samples': 20}
LightGBM	22	21/9	IgG anti-ApoB-100$^{1662\text{-}1683}$ MDA	1.380	0.968	0.122	0.978/-0.967	{'max_depth': 2, 'n_estimators': 30, 'learning_rate': 0.1, 'reg_lambda': 20.0, 'min_child_samples': 20}
LightGBM	22	21/9	IgG anti-C4A/B$^{167\text{-}187}$	2.555	0.968	0.122	0.978/-0.967	{'max_depth': 2, 'n_estimators': 30, 'learning_rate': 0.1, 'reg_lambda': 20.0, 'min_child_samples': 20}
LightGBM	23	21/9	IgG anti-ApoB-100$^{1662\text{-}1683}$ MDA	1.146	0.963	0.094	0.827/-0.980	{'max_depth': 2, 'n_estimators': 30, 'learning_rate': 0.1, 'reg_lambda': 20.0, 'min_child_samples': 20}
LightGBM	23	21/9	IgG anti-C4A/B$^{167\text{-}187}$	2.921	0.963	0.094	0.827/-0.980	{'max_depth': 2, 'n_estimators': 30, 'learning_rate': 0.1, 'reg_lambda': 20.0, 'min_child_samples': 20}
LightGBM	24	21/9	IgG anti-ApoB-100$^{1662\text{-}1683}$ MDA	1.436	0.861	0.111	0.558/0.034	{'max_depth': 2, 'n_estimators': 30, 'learning_rate': 0.1, 'reg_lambda': 20.0, 'min_child_samples': 20}
LightGBM	24	21/9	IgG anti-C4A/B$^{167\text{-}187}$	2.820	0.861	0.111	0.558/0.034	{'max_depth': 2, 'n_estimators': 30, 'learning_rate': 0.1, 'reg_lambda': 20.0, 'min_child_samples': 20}
LightGBM	25	21/9	IgG anti-ApoB-100$^{1662\text{-}1683}$ MDA	1.636	0.981	0.065	11.638/13.358	{'max_depth': 2, 'n_estimators': 30, 'learning_rate': 0.1,

								'reg_lambda': 20.0, 'min_child_samples': 20}
LightGBM	25	21/9	IgG anti-C4A/B$^{167-187}$	2.256	0.981	0.065	11.638/13.358	{'max_depth': 2, 'n_estimators': 30, 'learning_rate': 0.1, 'reg_lambda': 20.0, 'min_child_samples': 20}
CatBoost	1	21/9	IgG anti-ApoB-100$^{1662-1683}$ MDA	0.094	1.000	0.041	68.547/-26.083	{'iterations': 200, 'depth': 2, 'l2_leaf_reg': 20}
CatBoost	1	21/9	IgG anti-C4A/B$^{167-187}$	0.282	1.000	0.041	68.547/-26.083	{'iterations': 200, 'depth': 2, 'l2_leaf_reg': 20}
CatBoost	2	21/9	IgG anti-ApoB-100$^{1662-1683}$ MDA	0.185	0.926	0.111	0.981/-0.478	{'iterations': 200, 'depth': 2, 'l2_leaf_reg': 20}
CatBoost	2	21/9	IgG anti-C4A/B$^{167-187}$	0.238	0.926	0.111	0.981/-0.478	{'iterations': 200, 'depth': 2, 'l2_leaf_reg': 20}
CatBoost	3	21/9	IgG anti-ApoB-100$^{1662-1683}$ MDA	0.132	1.000	0.060	51.021/-18.292	{'iterations': 200, 'depth': 2, 'l2_leaf_reg': 20}
CatBoost	3	21/9	IgG anti-C4A/B$^{167-187}$	0.233	1.000	0.060	51.021/-18.292	{'iterations': 200, 'depth': 2, 'l2_leaf_reg': 20}
CatBoost	4	21/9	IgG anti-ApoB-100$^{1662-1683}$ MDA	0.081	0.769	0.168	0.203/-0.208	{'iterations': 200, 'depth': 2, 'l2_leaf_reg': 20}
CatBoost	4	21/9	IgG anti-C4A/B$^{167-187}$	0.408	0.769	0.168	0.203/-0.208	{'iterations': 200, 'depth': 2, 'l2_leaf_reg': 20}
CatBoost	5	21/9	IgG anti-ApoB-100$^{1662-1683}$ MDA	0.145	0.972	0.100	1.130/1.809	{'iterations': 200, 'depth': 2, 'l2_leaf_reg': 20}
CatBoost	5	21/9	IgG anti-C4A/B$^{167-187}$	0.237	0.972	0.100	1.130/1.809	{'iterations': 200, 'depth': 2, 'l2_leaf_reg': 20}
CatBoost	6	21/9	IgG anti-ApoB-100$^{1662-1683}$ MDA	0.110	1.000	0.064	33.362/-21.163	{'iterations': 200, 'depth': 2, 'l2_leaf_reg': 20}
CatBoost	6	21/9	IgG anti-C4A/B$^{167-187}$	0.291	1.000	0.064	33.362/-21.163	{'iterations': 200, 'depth': 2, 'l2_leaf_reg': 20}
CatBoost	7	21/9	IgG anti-ApoB-100$^{1662-1683}$ MDA	0.187	0.972	0.072	2.024/-0.246	{'iterations': 200, 'depth': 2, 'l2_leaf_reg': 20}
CatBoost	7	21/9	IgG anti-C4A/B$^{167-187}$	0.239	0.972	0.072	2.024/-0.246	{'iterations': 200, 'depth': 2, 'l2_leaf_reg': 20}
CatBoost	8	21/9	IgG anti-ApoB-100$^{1662-1683}$ MDA	0.095	0.815	0.160	0.228/-0.225	{'iterations': 200, 'depth': 2, 'l2_leaf_reg': 20}

CatBoost	8	21/9	IgG anti-C4A/B$^{167-187}$	0.397	0.815	0.160	0.228/-0.225	{'iterations': 200, 'depth': 2, 'l2_leaf_reg': 20}
CatBoost	9	21/9	IgG anti-ApoB-100$^{1662-1683}$ MDA	0.137	0.972	0.088	1.488/1.089	{'iterations': 200, 'depth': 2, 'l2_leaf_reg': 20}
CatBoost	9	21/9	IgG anti-C4A/B$^{167-187}$	0.239	0.972	0.088	1.488/1.089	{'iterations': 200, 'depth': 2, 'l2_leaf_reg': 20}
CatBoost	10	21/9	IgG anti-ApoB-100$^{1662-1683}$ MDA	0.142	0.944	0.062	1.536/0.647	{'iterations': 200, 'depth': 2, 'l2_leaf_reg': 20}
CatBoost	10	21/9	IgG anti-C4A/B$^{167-187}$	0.263	0.944	0.062	1.536/0.647	{'iterations': 200, 'depth': 2, 'l2_leaf_reg': 20}
CatBoost	11	21/9	IgG anti-ApoB-100$^{1662-1683}$ MDA	0.171	1.000	0.042	17.930/-11.453	{'iterations': 200, 'depth': 2, 'l2_leaf_reg': 20}
CatBoost	11	21/9	IgG anti-C4A/B$^{167-187}$	0.250	1.000	0.042	17.930/-11.453	{'iterations': 200, 'depth': 2, 'l2_leaf_reg': 20}
CatBoost	12	21/9	IgG anti-ApoB-100$^{1662-1683}$ MDA	0.114	0.907	0.084	0.663/0.013	{'iterations': 200, 'depth': 2, 'l2_leaf_reg': 20}
CatBoost	12	21/9	IgG anti-C4A/B$^{167-187}$	0.313	0.907	0.084	0.663/0.013	{'iterations': 200, 'depth': 2, 'l2_leaf_reg': 20}
CatBoost	13	21/9	IgG anti-ApoB-100$^{1662-1683}$ MDA	0.197	0.926	0.119	1.258/0.406	{'iterations': 200, 'depth': 2, 'l2_leaf_reg': 20}
CatBoost	13	21/9	IgG anti-C4A/B$^{167-187}$	0.189	0.926	0.119	1.258/0.406	{'iterations': 200, 'depth': 2, 'l2_leaf_reg': 20}
CatBoost	14	21/9	IgG anti-ApoB-100$^{1662-1683}$ MDA	0.079	0.852	0.183	0.304/-0.210	{'iterations': 200, 'depth': 2, 'l2_leaf_reg': 20}
CatBoost	14	21/9	IgG anti-C4A/B$^{167-187}$	0.372	0.852	0.183	0.304/-0.210	{'iterations': 200, 'depth': 2, 'l2_leaf_reg': 20}
CatBoost	15	21/9	IgG anti-ApoB-100$^{1662-1683}$ MDA	0.179	0.991	0.057	1.905/0.567	{'iterations': 200, 'depth': 2, 'l2_leaf_reg': 20}
CatBoost	15	21/9	IgG anti-C4A/B$^{167-187}$	0.242	0.991	0.057	1.905/0.567	{'iterations': 200, 'depth': 2, 'l2_leaf_reg': 20}
CatBoost	16	21/9	IgG anti-ApoB-100$^{1662-1683}$ MDA	0.135	0.824	0.158	0.592/0.252	{'iterations': 200, 'depth': 2, 'l2_leaf_reg': 20}
CatBoost	16	21/9	CRI I	0.191	0.824	0.158	0.592/0.252	{'iterations': 200, 'depth': 2, 'l2_leaf_reg': 20}
CatBoost	17	21/9	IgG anti-ApoB-100$^{1662-1683}$ MDA	0.224	0.944	0.121	1.033/-0.722	{'iterations': 200, 'depth': 2, 'l2_leaf_reg': 20}
CatBoost	17	21/9	IgG anti-C4A/B$^{167-187}$	0.202	0.944	0.121	1.033/-0.722	{'iterations': 200, 'depth': 2, 'l2_leaf_reg': 20}

CatBoost	18	21/9	IgG anti-ApoB-100$^{1662\text{-}1683}$ MDA	0.153	0.944	0.129	1.570/1.689	{'iterations': 200, 'depth': 2, 'l2_leaf_reg': 20}
CatBoost	18	21/9	IgG anti-C4A/B$^{167\text{-}187}$	0.239	0.944	0.129	1.570/1.689	{'iterations': 200, 'depth': 2, 'l2_leaf_reg': 20}
CatBoost	19	21/9	IgG anti-ApoB-100$^{1662\text{-}1683}$ MDA	0.083	0.889	0.072	0.648/-0.256	{'iterations': 200, 'depth': 2, 'l2_leaf_reg': 20}
CatBoost	19	21/9	IgG anti-C4A/B$^{167\text{-}187}$	0.364	0.889	0.072	0.648/-0.256	{'iterations': 200, 'depth': 2, 'l2_leaf_reg': 20}
CatBoost	20	21/9	IgG anti-ApoB-100$^{1662\text{-}1683}$ MDA	0.107	0.898	0.131	0.498/-0.401	{'iterations': 200, 'depth': 2, 'l2_leaf_reg': 20}
CatBoost	20	21/9	IgG anti-C4A/B$^{167\text{-}187}$	0.321	0.898	0.131	0.498/-0.401	{'iterations': 200, 'depth': 2, 'l2_leaf_reg': 20}
CatBoost	21	21/9	IgG anti-ApoB-100$^{1662\text{-}1683}$ MDA	0.104	0.944	0.098	0.968/0.717	{'iterations': 200, 'depth': 2, 'l2_leaf_reg': 20}
CatBoost	21	21/9	IgG anti-C4A/B$^{167\text{-}187}$	0.282	0.944	0.098	0.968/0.717	{'iterations': 200, 'depth': 2, 'l2_leaf_reg': 20}
CatBoost	22	21/9	IgG anti-ApoB-100$^{1662\text{-}1683}$ MDA	0.121	0.815	0.124	0.313/-0.285	{'iterations': 200, 'depth': 2, 'l2_leaf_reg': 20}
CatBoost	22	21/9	IgG anti-C4A/B$^{167\text{-}187}$	0.350	0.815	0.124	0.313/-0.285	{'iterations': 200, 'depth': 2, 'l2_leaf_reg': 20}
CatBoost	23	21/9	IgG anti-ApoB-100$^{1662\text{-}1683}$ MDA	0.129	1.000	0.036	12.902/-9.505	{'iterations': 200, 'depth': 2, 'l2_leaf_reg': 20}
CatBoost	23	21/9	IgG anti-C4A/B$^{167\text{-}187}$	0.285	1.000	0.036	12.902/-9.505	{'iterations': 200, 'depth': 2, 'l2_leaf_reg': 20}
CatBoost	24	21/9	IgG anti-ApoB-100$^{1662\text{-}1683}$ MDA	0.125	0.935	0.105	0.991/0.486	{'iterations': 200, 'depth': 2, 'l2_leaf_reg': 20}
CatBoost	24	21/9	IgG anti-C4A/B$^{167\text{-}187}$	0.274	0.935	0.105	0.991/0.486	{'iterations': 200, 'depth': 2, 'l2_leaf_reg': 20}
CatBoost	25	21/9	IgG anti-ApoB-100$^{1662\text{-}1683}$ MDA	0.172	0.981	0.065	2.015/0.253	{'iterations': 200, 'depth': 2, 'l2_leaf_reg': 20}
CatBoost	25	21/9	IgG anti-C4A/B$^{167\text{-}187}$	0.251	0.981	0.065	2.015/0.253	{'iterations': 200, 'depth': 2, 'l2_leaf_reg': 20}
LR	1	21/9	IgG anti-ApoB-100$^{1662\text{-}1683}$ MDA	0.617	0.898	0.142	3.207/-0.910	{'C': 1.0, 'l1_ratio': 0.5, 'penalty': 'l2'}
LR	1	21/9	CRI II	0.609	0.898	0.142	3.207/-0.910	{'C': 1.0, 'l1_ratio': 0.5, 'penalty': 'l2'}
LR	2	21/9	IgG anti-ApoB-100$^{1662\text{-}1683}$ MDA	1.693	0.796	0.199	0.626/-0.384	{'C': 1.0, 'l1_ratio': 0.5, 'penalty': 'l2'}

LR	2	21/9	CRI II	0.668	0.796	0.199	0.626/-0.384	{'C': 1.0, 'l1_ratio': 0.5, 'penalty': 'l2'}
LR	3	21/9	IgG anti-ApoB-100$^{1662-1683}$ MDA	0.561	0.852	0.160	1.606/0.338	{'C': 1.0, 'l1_ratio': 0.5, 'penalty': 'l2'}
LR	3	21/9	CRI II	0.624	0.852	0.160	1.606/0.338	{'C': 1.0, 'l1_ratio': 0.5, 'penalty': 'l2'}
LR	4	21/9	IgG anti-ApoB-100$^{1662-1683}$ MDA	0.786	0.870	0.143	1.105/-0.195	{'C': 1.0, 'l1_ratio': 0.5, 'penalty': 'l2'}
LR	4	21/9	CRI II	0.955	0.870	0.143	1.105/-0.195	{'C': 1.0, 'l1_ratio': 0.5, 'penalty': 'l2'}
LR	5	21/9	IgG anti-ApoB-100$^{1662-1683}$ MDA	1.521	0.972	0.100	1.130/1.809	{'C': 1.0, 'l1_ratio': 0.5, 'penalty': 'l2'}
LR	5	21/9	IgG anti-C4A/B$^{167-187}$	2.120	0.972	0.100	1.130/1.809	{'C': 1.0, 'l1_ratio': 0.5, 'penalty': 'l2'}
LR	6	21/9	IgG anti-ApoB-100$^{1662-1683}$ MDA	0.716	0.861	0.153	1.802/-0.024	{'C': 1.0, 'l1_ratio': 0.5, 'penalty': 'l2'}
LR	6	21/9	CRI II	0.485	0.861	0.153	1.802/-0.024	{'C': 1.0, 'l1_ratio': 0.5, 'penalty': 'l2'}
LR	7	21/9	IgG anti-ApoB-100$^{1662-1683}$ MDA	0.819	0.843	0.148	0.395/-0.434	{'C': 1.0, 'l1_ratio': 0.5, 'penalty': 'l2'}
LR	7	21/9	MDA-protein adduct	1.501	0.843	0.148	0.395/-0.434	{'C': 1.0, 'l1_ratio': 0.5, 'penalty': 'l2'}
LR	8	21/9	IgG anti-ApoB-100$^{1662-1683}$ MDA	1.138	0.787	0.186	0.548/-0.042	{'C': 1.0, 'l1_ratio': 0.5, 'penalty': 'l2'}
LR	8	21/9	CRI II	0.797	0.787	0.186	0.548/-0.042	{'C': 1.0, 'l1_ratio': 0.5, 'penalty': 'l2'}
LR	9	21/9	IgG anti-ApoB-100$^{1662-1683}$ MDA	0.783	0.713	0.200	0.402/0.200	{'C': 1.0, 'l1_ratio': 0.5, 'penalty': 'l2'}
LR	9	21/9	CRI I	1.391	0.713	0.200	0.402/0.200	{'C': 1.0, 'l1_ratio': 0.5, 'penalty': 'l2'}
LR	10	21/9	IgG anti-ApoB-100$^{1662-1683}$ MDA	0.773	0.870	0.140	1.603/0.133	{'C': 1.0, 'l1_ratio': 0.5, 'penalty': 'l2'}
LR	10	21/9	CRI II	0.653	0.870	0.140	1.603/0.133	{'C': 1.0, 'l1_ratio': 0.5, 'penalty': 'l2'}
LR	11	21/9	IgG anti-ApoB-100$^{1662-1683}$ MDA	0.890	0.907	0.132	1.964/-0.447	{'C': 1.0, 'l1_ratio': 0.5, 'penalty': 'l2'}
LR	11	21/9	CRI II	0.628	0.907	0.132	1.964/-0.447	{'C': 1.0, 'l1_ratio': 0.5, 'penalty': 'l2'}

LR	12	21/9	IgG anti-ApoB-100$^{1662-1683}$ MDA	1.112	0.907	0.084	0.663/0.013	{'C': 1.0, 'l1_ratio': 0.5, 'penalty': 'l2'}
LR	12	21/9	IgG anti-C4A/B$^{167-187}$	2.839	0.907	0.084	0.663/0.013	{'C': 1.0, 'l1_ratio': 0.5, 'penalty': 'l2'}
LR	13	21/9	IgG anti-ApoB-100$^{1662-1683}$ MDA	1.015	0.537	0.311	0.089/-0.209	{'C': 1.0, 'l1_ratio': 0.5, 'penalty': 'l2'}
LR	13	21/9	CRI II	1.268	0.537	0.311	0.089/-0.209	{'C': 1.0, 'l1_ratio': 0.5, 'penalty': 'l2'}
LR	14	21/9	IgG anti-ApoB-100$^{1662-1683}$ MDA	0.779	0.824	0.156	1.446/0.248	{'C': 1.0, 'l1_ratio': 0.5, 'penalty': 'l2'}
LR	14	21/9	CRI II	0.705	0.824	0.156	1.446/0.248	{'C': 1.0, 'l1_ratio': 0.5, 'penalty': 'l2'}
LR	15	21/9	IgG anti-ApoB-100$^{1662-1683}$ MDA	1.458	0.991	0.057	1.905/0.567	{'C': 1.0, 'l1_ratio': 0.5, 'penalty': 'l2'}
LR	15	21/9	IgG anti-C4A/B$^{167-187}$	1.795	0.991	0.057	1.905/0.567	{'C': 1.0, 'l1_ratio': 0.5, 'penalty': 'l2'}
LR	16	21/9	IgG anti-ApoB-100$^{1662-1683}$ MDA	0.789	0.824	0.158	0.592/0.252	{'C': 1.0, 'l1_ratio': 0.5, 'penalty': 'l2'}
LR	16	21/9	CRI I	1.354	0.824	0.158	0.592/0.252	{'C': 1.0, 'l1_ratio': 0.5, 'penalty': 'l2'}
LR	17	21/9	IgG anti-ApoB-100$^{1662-1683}$ MDA	1.007	0.731	0.228	0.516/-0.447	{'C': 1.0, 'l1_ratio': 0.5, 'penalty': 'l2'}
LR	17	21/9	CRI II	0.748	0.731	0.228	0.516/-0.447	{'C': 1.0, 'l1_ratio': 0.5, 'penalty': 'l2'}
LR	18	21/9	IgG anti-ApoB-100$^{1662-1683}$ MDA	0.966	0.639	0.224	0.414/0.039	{'C': 1.0, 'l1_ratio': 0.5, 'penalty': 'l2'}
LR	18	21/9	CRI II	0.711	0.639	0.224	0.414/0.039	{'C': 1.0, 'l1_ratio': 0.5, 'penalty': 'l2'}
LR	19	21/9	IgG anti-ApoB-100$^{1662-1683}$ MDA	1.103	0.889	0.072	0.648/-0.256	{'C': 1.0, 'l1_ratio': 0.5, 'penalty': 'l2'}
LR	19	21/9	IgG anti-C4A/B$^{167-187}$	3.317	0.889	0.072	0.648/-0.256	{'C': 1.0, 'l1_ratio': 0.5, 'penalty': 'l2'}
LR	20	21/9	IgG anti-ApoB-100$^{1662-1683}$ MDA	0.951	0.833	0.165	1.272/0.177	{'C': 1.0, 'l1_ratio': 0.5, 'penalty': 'l2'}
LR	20	21/9	CRI II	0.815	0.833	0.165	1.272/0.177	{'C': 1.0, 'l1_ratio': 0.5, 'penalty': 'l2'}
LR	21	21/9	IgG anti-ApoB-100$^{1662-1683}$ MDA	1.167	0.944	0.098	0.968/0.717	{'C': 1.0, 'l1_ratio': 0.5, 'penalty': 'l2'}

LR	21	21/9	IgG anti-C4A/B$^{167-187}$	2.077	0.944	0.098	0.968/0.717	{'C': 1.0, 'l1_ratio': 0.5, 'penalty': 'l2'}
LR	22	21/9	IgG anti-ApoB-100$^{1662-1683}$ MDA	0.888	0.796	0.179	0.740/-0.185	{'C': 1.0, 'l1_ratio': 0.5, 'penalty': 'l2'}
LR	22	21/9	CRI II	1.059	0.796	0.179	0.740/-0.185	{'C': 1.0, 'l1_ratio': 0.5, 'penalty': 'l2'}
LR	23	21/9	IgG anti-ApoB-100$^{1662-1683}$ MDA	0.832	0.870	0.146	1.049/0.209	{'C': 1.0, 'l1_ratio': 0.5, 'penalty': 'l2'}
LR	23	21/9	CRI II	0.832	0.870	0.146	1.049/0.209	{'C': 1.0, 'l1_ratio': 0.5, 'penalty': 'l2'}
LR	24	21/9	IgG anti-ApoB-100$^{1662-1683}$ MDA	0.961	0.796	0.178	1.043/0.286	{'C': 1.0, 'l1_ratio': 0.5, 'penalty': 'l2'}
LR	24	21/9	CRI II	0.406	0.796	0.178	1.043/0.286	{'C': 1.0, 'l1_ratio': 0.5, 'penalty': 'l2'}
LR	25	21/9	IgG anti-ApoB-100$^{1662-1683}$ MDA	1.468	0.981	0.065	2.015/0.253	{'C': 1.0, 'l1_ratio': 0.5, 'penalty': 'l2'}
LR	25	21/9	IgG anti-C4A/B$^{167-187}$	1.858	0.981	0.065	2.015/0.253	{'C': 1.0, 'l1_ratio': 0.5, 'penalty': 'l2'}
T2DM vs. T2DM/CAD ≥70% Stenosis								
RF	1	24/12	IgG anti-ApoB-100$^{1662-1683}$ MDA	0.140	0.986	0.047	3.262/-0.597	{'max_depth': 2, 'n_estimators': 50}
RF	1	24/12	IgM anti-FINC$^{959-984}$	0.120	0.986	0.047	3.262/-0.597	{'max_depth': 2, 'n_estimators': 50}
RF	1	24/12	IgG anti-C4A/B$^{167-187}$	0.217	0.986	0.047	3.262/-0.597	{'max_depth': 2, 'n_estimators': 50}
RF	2	24/12	IgG anti-ApoB-100$^{1662-1683}$ MDA	0.205	0.826	0.194	1.049/-0.985	{'max_depth': 3, 'n_estimators': 50}
RF	2	24/12	IgM anti-FINC$^{959-984}$	0.129	0.826	0.194	1.049/-0.985	{'max_depth': 3, 'n_estimators': 50}
RF	2	24/12	CRI II	0.114	0.826	0.194	1.049/-0.985	{'max_depth': 3, 'n_estimators': 50}
RF	3	24/12	IgG anti-ApoB-100$^{1662-1683}$ MDA	0.167	0.990	0.081	2.137/2.070	{'max_depth': 2, 'n_estimators': 50}
RF	3	24/12	IgM anti-FINC$^{959-984}$	0.123	0.990	0.081	2.137/2.070	{'max_depth': 2, 'n_estimators': 50}
RF	3	24/12	IgG anti-C4A/B$^{167-187}$	0.223	0.990	0.081	2.137/2.070	{'max_depth': 2, 'n_estimators': 50}

RF	4	24/12	IgG anti-ApoB-100$^{1662-1683}$ MDA	0.166	0.785	0.194	0.577/0.066	{'max_depth': 2, 'n_estimators': 50}
RF	4	24/12	PCr	0.113	0.785	0.194	0.577/0.066	{'max_depth': 2, 'n_estimators': 50}
RF	4	24/12	CRI II	0.158	0.785	0.194	0.577/0.066	{'max_depth': 2, 'n_estimators': 50}
RF	5	24/12	IgG anti-ApoB-100$^{1662-1683}$ MDA	0.145	1.000	0.023	15.507/1.629	{'max_depth': 2, 'n_estimators': 50}
RF	5	24/12	IgM anti-FINC$^{959-984}$	0.137	1.000	0.023	15.507/1.629	{'max_depth': 2, 'n_estimators': 50}
RF	5	24/12	IgG anti-C4A/B$^{167-187}$	0.173	1.000	0.023	15.507/1.629	{'max_depth': 2, 'n_estimators': 50}
RF	6	24/12	IgG anti-ApoB-100$^{1662-1683}$ MDA	0.136	0.937	0.116	1.114/-0.406	{'max_depth': 2, 'n_estimators': 50}
RF	6	24/12	IgM anti-FINC$^{959-984}$	0.155	0.937	0.116	1.114/-0.406	{'max_depth': 2, 'n_estimators': 50}
RF	6	24/12	IgG anti-C4A/B$^{167-187}$	0.184	0.937	0.116	1.114/-0.406	{'max_depth': 2, 'n_estimators': 50}
RF	7	24/12	IgG anti-ApoB-100$^{1662-1683}$ MDA	0.171	0.972	0.072	1.545/-0.285	{'max_depth': 2, 'n_estimators': 50}
RF	7	24/12	IgM anti-FINC$^{959-984}$	0.112	0.972	0.072	1.545/-0.285	{'max_depth': 2, 'n_estimators': 50}
RF	7	24/12	IgG anti-C4A/B$^{167-187}$	0.235	0.972	0.072	1.545/-0.285	{'max_depth': 2, 'n_estimators': 50}
RF	8	24/12	IgG anti-ApoB-100$^{1662-1683}$ MDA	0.144	0.965	0.065	3.055/-1.078	{'max_depth': 2, 'n_estimators': 50}
RF	8	24/12	IgM anti-FINC$^{959-984}$	0.111	0.965	0.065	3.055/-1.078	{'max_depth': 2, 'n_estimators': 50}
RF	8	24/12	IgG anti-C4A/B$^{167-187}$	0.213	0.965	0.065	3.055/-1.078	{'max_depth': 2, 'n_estimators': 50}
RF	9	24/12	IgG anti-APOB-100$^{1662-1683}$ MDA	0.192	0.875	0.131	0.600/0.421	{'max_depth': 2, 'n_estimators': 50}
RF	9	24/12	IgG anti-C4A/B$^{167-187}$	0.177	0.875	0.131	0.600/0.421	{'max_depth': 2, 'n_estimators': 50}
RF	9	24/12	CRI II	0.129	0.875	0.131	0.600/0.421	{'max_depth': 2, 'n_estimators': 50}
RF	10	24/12	IgG anti-ApoB-100$^{1662-1683}$ MDA	0.191	0.931	0.125	1.337/-0.177	{'max_depth': 3, 'n_estimators': 50}

RF	10	24/12	IgM anti-FINC$^{959-984}$	0.102	0.931	0.125	1.337/-0.177	{'max_depth': 3, 'n_estimators': 50}
RF	10	24/12	CRI II	0.109	0.931	0.125	1.337/-0.177	{'max_depth': 3, 'n_estimators': 50}
RF	11	24/12	IgG anti-ApoB-100$^{1662-1683}$ MDA	0.179	0.917	0.111	1.449/0.020	{'max_depth': 2, 'n_estimators': 50}
RF	11	24/12	IgM anti-FINC$^{959-984}$	0.128	0.917	0.111	1.449/0.020	{'max_depth': 2, 'n_estimators': 50}
RF	11	24/12	CRI II	0.140	0.917	0.111	1.449/0.020	{'max_depth': 2, 'n_estimators': 50}
RF	12	24/12	IgG anti-ApoB-100$^{1662-1683}$ MDA	0.138	0.937	0.121	1.423/-1.231	{'max_depth': 2, 'n_estimators': 50}
RF	12	24/12	IgG anti-C4A/B$^{167-187}$	0.191	0.937	0.121	1.423/-1.231	{'max_depth': 2, 'n_estimators': 50}
RF	12	24/12	CRI II	0.144	0.937	0.121	1.423/-1.231	{'max_depth': 2, 'n_estimators': 50}
RF	13	24/12	IgG anti-ApoB-100$^{1662-1683}$ MDA	0.1715	0.979	0.083	1.960/0.905	{'max_depth': 3, 'n_estimators': 50}
RF	13	24/12	IgG anti-C4A/B$^{167-187}$	0.1864	0.979	0.083	1.960/0.905	{'max_depth': 3, 'n_estimators': 50}
RF	13	24/12	CRI II	0.0885	0.979	0.083	1.960/0.905	{'max_depth': 3, 'n_estimators': 50}
RF	14	24/12	IgG anti-ApoB-100$^{1662-1683}$ MDA	0.167	0.924	0.112	1.096/0.135	{'max_depth': 2, 'n_estimators': 50}
RF	14	24/12	IgM anti-FINC$^{959-984}$	0.104	0.924	0.112	1.096/0.135	{'max_depth': 2, 'n_estimators': 50}
RF	14	24/12	IgG anti-C4A/B$^{167-187}$	0.171	0.924	0.112	1.096/0.135	{'max_depth': 2, 'n_estimators': 50}
RF	15	24/12	IgG anti-ApoB-100$^{1662-1683}$ MDA	0.139	0.972	0.078	2.405/-1.055	{'max_depth': 2, 'n_estimators': 50}
RF	15	24/12	IgM anti-FINC$^{959-984}$	0.138	0.972	0.078	2.405/-1.055	{'max_depth': 2, 'n_estimators': 50}
RF	15	24/12	IgG anti-C4A/B$^{167-187}$	0.236	0.972	0.078	2.405/-1.055	{'max_depth': 2, 'n_estimators': 50}
RF	16	24/12	IgG anti-ApoB-100$^{1662-1683}$ MDA	0.222	0.847	0.152	0.739/0.725	{'max_depth': 2, 'n_estimators': 50}
RF	16	24/12	IgG anti-C4A/B$^{167-187}$	0.181	0.847	0.152	0.739/0.725	{'max_depth': 2, 'n_estimators': 50}

RF	16	24/12	CRI II	0.094	0.847	0.152	0.739/0.725	{'max_depth': 2, 'n_estimators': 50}
RF	17	24/12	IgG anti-ApoB-100$^{1662-1683}$ MDA	0.147	0.986	0.077	2.533/-1.288	{'max_depth': 2, 'n_estimators': 50}
RF	17	24/12	IgM anti-FINC$^{959-984}$	0.129	0.986	0.077	2.533/1.288	{'max_depth': 2, 'n_estimators': 50}
RF	17	24/12	IgG anti-C4A/B$^{167-187}$	0.209	0.986	0.077	2.533/-1.288	{'max_depth': 2, 'n_estimators': 50}
RF	18	24/12	IgG anti-ApoB-100$^{1662-1683}$ MDA	0.152	0.944	0.091	1.266/-0.233	{'max_depth': 2, 'n_estimators': 50}
RF	18	24/12	IgG anti-C4A/B$^{167-187}$	0.185	0.944	0.091	1.266/-0.233	{'max_depth': 2, 'n_estimators': 50}
RF	18	24/12	CRI II	0.108	0.944	0.091	1.266/-0.233	{'max_depth': 2, 'n_estimators': 50}
RF	19	24/12	IgG anti-ApoB-100$^{1662-1683}$ MDA	0.128	1.000	0.036	20.166/7.544	{'max_depth': 2, 'n_estimators': 50}
RF	19	24/12	IgM anti-FINC$^{959-984}$	0.136	1.000	0.036	20.166/7.544	{'max_depth': 2, 'n_estimators': 50}
RF	19	24/12	IgG anti-C4A/B$^{167-187}$	0.215	1.000	0.036	20.166/7.544	{'max_depth': 2, 'n_estimators': 50}
RF	20	24/12	IgG anti-ApoB-100$^{1662-1683}$ MDA	0.144	0.854	0.148	0.566/0.054	{'max_depth': 2, 'n_estimators': 50}
RF	20	24/12	IgM anti-FINC$^{959-984}$	0.166	0.854	0.148	0.566/0.054	{'max_depth': 2, 'n_estimators': 50}
RF	20	24/12	IgG anti-C4A/B$^{167-187}$	0.223	0.854	0.148	0.566/0.054	{'max_depth': 2, 'n_estimators': 50}
RF	21	24/12	IgG anti-ApoB-100$^{1662-1683}$ MDA	0.149	1.000	0.035	24.241/-6.317	{'max_depth': 2, 'n_estimators': 50}
RF	21	24/12	IgM anti-FINC$^{959-984}$	0.141	1.000	0.035	24.241/-6.317	{'max_depth': 2, 'n_estimators': 50}
RF	21	24/12	IgG anti-C4A/B$^{167-187}$	0.198	1.000	0.035	24.241/-6.317	{'max_depth': 2, 'n_estimators': 50}
RF	22	24/12	IgG anti-ApoB-100$^{1662-1683}$ MDA	0.167	0.965	0.085	2.124/1.227	{'max_depth': 2, 'n_estimators': 50}
RF	22	24/12	IgM anti-FINC$^{959-984}$	0.133	0.965	0.085	2.124/1.227	{'max_depth': 2, 'n_estimators': 50}
RF	22	24/12	IgG anti-C4A/B$^{167-187}$	0.217	0.965	0.085	2.124/1.227	{'max_depth': 2, 'n_estimators': 50}

RF	23	24/12	IgG anti-ApoB-100$^{1662-1683}$ MDA	0.178	0.951	0.102	1.557/0.375	{'max_depth': 3, 'n_estimators': 50}
RF	23	24/12	IgM anti-FINC$^{959-984}$	0.108	0.951	0.102	1.557/0.375	{'max_depth': 3, 'n_estimators': 50}
RF	23	24/12	CRI II	0.124	0.951	0.102	1.557/0.375	{'max_depth': 3, 'n_estimators': 50}
RF	24	24/12	IgG anti-ApoB-100$^{1662-1683}$ MDA	0.187	0.861	0.158	0.852/0.118	{'max_depth': 2, 'n_estimators': 50}
RF	24	24/12	MDA-potein adduct	0.109	0.861	0.158	0.852/0.118	{'max_depth': 2, 'n_estimators': 50}
RF	24	24/12	CRI II	0.163	0.861	0.158	0.852/0.118	{'max_depth': 2, 'n_estimators': 50}
RF	25	24/12	IgG anti-ApoB-100$^{1662-1683}$ MDA	0.138	0.931	0.122	1.327/-1.048	{'max_depth': 3, 'n_estimators': 50}
RF	25	24/12	IgM anti-FINC$^{959-984}$	0.108	0.931	0.122	1.327/-1.048	{'max_depth': 3, 'n_estimators': 50}
RF	25	24/12	IgG anti-C4A/B$^{167-187}$	0.214	0.931	0.122	1.327/-1.048	{'max_depth': 3, 'n_estimators': 50}
XGBoost	1	24/12	IgG anti-ApoB-100$^{1662-1683}$ MDA	1.623	0.986	0.038	1.319/-0.026	{'max_depth': 2, 'n_estimators': 30, 'learning_rate': 0.1, 'reg_lambda': 20.0, 'min_child_weight': 3}
XGBoost	1	24/12	IgM anti-FINC$^{959-984}$	1.409	0.986	0.038	1.319/-0.026	{'max_depth': 2, 'n_estimators': 30, 'learning_rate': 0.1, 'reg_lambda': 20.0, 'min_child_weight': 3}
XGBoost	1	24/12	IgG anti-C4A/B$^{167-187}$	1.922	0.986	0.038	1.319/-0.026	{'max_depth': 2, 'n_estimators': 30, 'learning_rate': 0.1, 'reg_lambda': 20.0, 'min_child_weight': 3}
XGBoost	2	24/12	IgG anti-ApoB-100$^{1662-1683}$ MDA	1.478	0.812	0.208	0.665/-0.734	{'max_depth': 2, 'n_estimators': 30, 'learning_rate': 0.05, 'reg_lambda': 20.0, 'min_child_weight': 3}

XGBoost	2	24/12	IgM anti-FINC$^{959-984}$	1.240	0.812	0.208	0.665/-0.734	{'max_depth': 2, 'n_estimators': 30, 'learning_rate': 0.05, 'reg_lambda': 20.0, 'min_child_weight': 3}
XGBoost	2	24/12	CRI II	0.871	0.812	0.208	0.665/-0.734	{'max_depth': 2, 'n_estimators': 30, 'learning_rate': 0.05, 'reg_lambda': 20.0, 'min_child_weight': 3}
XGBoost	3	24/12	IgG anti-ApoB-100$^{1662-1683}$ MDA	1.095	0.972	0.063	1.739/2.900	{'max_depth': 2, 'n_estimators': 30, 'learning_rate': 0.05, 'reg_lambda': 20.0, 'min_child_weight': 3}
XGBoost	3	24/12	IgM anti-FINC$^{959-984}$	0.881	0.972	0.063	1.739/2.900	{'max_depth': 2, 'n_estimators': 30, 'learning_rate': 0.05, 'reg_lambda': 20.0, 'min_child_weight': 3}
XGBoost	3	24/12	IgG anti-C4A/B$^{167-187}$	1.927	0.972	0.063	1.739/2.900	{'max_depth': 2, 'n_estimators': 30, 'learning_rate': 0.05, 'reg_lambda': 20.0, 'min_child_weight': 3}
XGBoost	4	24/12	IgG anti-ApoB-100$^{1662-1683}$ MDA	1.552	0.840	0.199	0.514/0.058	{'max_depth': 2, 'n_estimators': 30, 'learning_rate': 0.1, 'reg_lambda': 20.0, 'min_child_weight': 3}
XGBoost	4	24/12	IgM anti-FINC$^{959-984}$	1.219	0.840	0.199	0.514/0.058	{'max_depth': 2, 'n_estimators': 30, 'learning_rate': 0.1, 'reg_lambda': 20.0, 'min_child_weight': 3}
XGBoost	4	24/12	CRI II	1.361	0.840	0.199	0.514/0.058	{'max_depth': 2, 'n_estimators': 30, 'learning_rate': 0.1,

								'reg_lambda': 20.0,<br>'min_child_weight': 3}
XGBoost	5	24/12	IgG anti-ApoB-100$^{1662-1683}$<br>MDA	0.667	0.979	0.045	1.511/1.976	{'max_depth': 2,<br>'n_estimators': 30,<br>'learning_rate': 0.1,<br>'reg_lambda': 20.0,<br>'min_child_weight': 3}
XGBoost	5	24/12	MDA-protein adduct	0.980	0.979	0.045	1.511/1.976	{'max_depth': 2,<br>'n_estimators': 30,<br>'learning_rate': 0.1,<br>'reg_lambda': 20.0,<br>'min_child_weight': 3}
XGBoost	5	24/12	IgG anti-C4A/B$^{167-187}$	2.062	0.979	0.045	1.511/1.976	{'max_depth': 2,<br>'n_estimators': 30,<br>'learning_rate': 0.1,<br>'reg_lambda': 20.0,<br>'min_child_weight': 3}
XGBoost	6	24/12	IgG anti-ApoB-100$^{1662-1683}$<br>MDA	0.946	0.865	0.187	0.422/0.026	{'max_depth': 2,<br>'n_estimators': 30,<br>'learning_rate': 0.1,<br>'reg_lambda': 20.0,<br>'min_child_weight': 3}
XGBoost	6	24/12	IgM anti-FINC$^{959-984}$	1.432	0.865	0.187	0.422/0.026	{'max_depth': 2,<br>'n_estimators': 30,<br>'learning_rate': 0.1,<br>'reg_lambda': 20.0,<br>'min_child_weight': 3}
XGBoost	6	24/12	IgG anti-C4A/B$^{167-187}$	1.932	0.865	0.187	0.422/0.026	{'max_depth': 2,<br>'n_estimators': 30,<br>'learning_rate': 0.1,<br>'reg_lambda': 20.0,<br>'min_child_weight': 3}
XGBoost	7	24/12	IgG anti-ApoB-100$^{1662-1683}$<br>MDA	1.136	0.948	0.104	0.783/-0.229	{'max_depth': 2,<br>'n_estimators': 30,<br>'learning_rate': 0.05,<br>'reg_lambda': 20.0,<br>'min_child_weight': 3}

XGBoost	7	24/12	IgM anti-FINC959-984	0.841	0.948	0.104	0.783/-0.229	{'max_depth': 2, 'n_estimators': 30, 'learning_rate': 0.05, 'reg_lambda': 20.0, 'min_child_weight': 3}
XGBoost	7	24/12	IgG anti-C4A/B167-187	2.049	0.948	0.104	0.783/-0.229	{'max_depth': 2, 'n_estimators': 30, 'learning_rate': 0.05, 'reg_lambda': 20.0, 'min_child_weight': 3}
XGBoost	8	24/12	IgG anti-ApoB-1001662-1683 MDA	1.400	0.896	0.152	0.724/0.263	{'max_depth': 2, 'n_estimators': 30, 'learning_rate': 0.1, 'reg_lambda': 20.0, 'min_child_weight': 3}
XGBoost	8	24/12	MDA-protein adduct	1.103	0.896	0.152	0.724/0.263	{'max_depth': 2, 'n_estimators': 30, 'learning_rate': 0.1, 'reg_lambda': 20.0, 'min_child_weight': 3}
XGBoost	8	24/12	CRI II	1.146	0.896	0.152	0.724/0.263	{'max_depth': 2, 'n_estimators': 30, 'learning_rate': 0.1, 'reg_lambda': 20.0, 'min_child_weight': 3}
XGBoost	9	24/12	IgG anti-ApoB-1001662-1683 MDA	2.114	0.865	0.138	0.419/0.535	{'max_depth': 2, 'n_estimators': 30, 'learning_rate': 0.1, 'reg_lambda': 20.0, 'min_child_weight': 3}
XGBoost	9	24/12	IgG anti-C4A/B167-187	1.904	0.865	0.138	0.419/0.535	{'max_depth': 2, 'n_estimators': 30, 'learning_rate': 0.1, 'reg_lambda': 20.0, 'min_child_weight': 3}
XGBoost	9	24/12	CRI II	1.438	0.865	0.138	0.419/0.535	{'max_depth': 2, 'n_estimators': 30, 'learning_rate': 0.1,

								'reg_lambda': 20.0, 'min_child_weight': 3}
XGBoost	10	24/12	IgG anti-ApoB-100$^{1662-1683}$ MDA	1.687	0.951	0.097	0.833/-0.247	{'max_depth': 2, 'n_estimators': 30, 'learning_rate': 0.1, 'reg_lambda': 20.0, 'min_child_weight': 3}
XGBoost	10	24/12	IgG anti-C4A/B$^{167-187}$	1.703	0.951	0.097	0.833/-0.247	{'max_depth': 2, 'n_estimators': 30, 'learning_rate': 0.1, 'reg_lambda': 20.0, 'min_child_weight': 3}
XGBoost	10	24/12	CRI II	1.146	0.951	0.097	0.833/-0.247	{'max_depth': 2, 'n_estimators': 30, 'learning_rate': 0.1, 'reg_lambda': 20.0, 'min_child_weight': 3}
XGBoost	11	24/12	IgG anti-ApoB-100$^{1662-1683}$ MDA	1.855	0.924	0.094	0.760/0.138	{'max_depth': 2, 'n_estimators': 30, 'learning_rate': 0.1, 'reg_lambda': 20.0, 'min_child_weight': 3}
XGBoost	11	24/12	MDA-protein adduct	0.844	0.924	0.094	0.760/0.138	{'max_depth': 2, 'n_estimators': 30, 'learning_rate': 0.1, 'reg_lambda': 20.0, 'min_child_weight': 3}
XGBoost	11	24/12	CRI II	1.276	0.924	0.094	0.760/0.138	{'max_depth': 2, 'n_estimators': 30, 'learning_rate': 0.1, 'reg_lambda': 20.0, 'min_child_weight': 3}
XGBoost	12	24/12	IgG anti-ApoB-100$^{1662-1683}$ MDA	1.144	0.972	0.113	1.813/-2.418	{'max_depth': 2, 'n_estimators': 30, 'learning_rate': 0.1, 'reg_lambda': 20.0, 'min_child_weight': 3}

XGBoost	12	24/12	IgM anti-FINC959-984	1.557	0.972	0.113	1.813/-2.418	{'max_depth': 2, 'n_estimators': 30, 'learning_rate': 0.1, 'reg_lambda': 20.0, 'min_child_weight': 3}
XGBoost	12	24/12	CRI II	1.330	0.972	0.113	1.813/-2.418	{'max_depth': 2, 'n_estimators': 30, 'learning_rate': 0.1, 'reg_lambda': 20.0, 'min_child_weight': 3}
XGBoost	13	24/12	IgG anti-ApoB-1001662-1683 MDA	1.264	1.000	0.029	14.688/6.306	{'max_depth': 2, 'n_estimators': 30, 'learning_rate': 0.1, 'reg_lambda': 20.0, 'min_child_weight': 3}
XGBoost	13	24/12	IgM anti-FINC959-984	1.386	1.000	0.029	14.688/6.306	{'max_depth': 2, 'n_estimators': 30, 'learning_rate': 0.1, 'reg_lambda': 20.0, 'min_child_weight': 3}
XGBoost	13	24/12	IgG anti-C4A/B167-187	2.101	1.000	0.029	14.688/6.306	{'max_depth': 2, 'n_estimators': 30, 'learning_rate': 0.1, 'reg_lambda': 20.0, 'min_child_weight': 3}
XGBoost	14	24/12	IgG anti-ApoB-1001662-1683 MDA	1.719	0.920	0.148	0.511/0.420	{'max_depth': 2, 'n_estimators': 30, 'learning_rate': 0.1, 'reg_lambda': 20.0, 'min_child_weight': 3}
XGBoost	14	24/12	IgM anti-FINC959-984	1.174	0.920	0.148	0.511/0.420	{'max_depth': 2, 'n_estimators': 30, 'learning_rate': 0.1, 'reg_lambda': 20.0, 'min_child_weight': 3}
XGBoost	14	24/12	IgG anti-C4A/B167-187	1.765	0.920	0.148	0.511/0.420	{'max_depth': 2, 'n_estimators': 30, 'learning_rate': 0.1,

								'reg_lambda': 20.0, 'min_child_weight': 3}
XGBoost	15	24/12	IgG anti-ApoB-100[1662-1683] MDA	1.001	0.948	0.086	1.184/-0.277	{'max_depth': 2, 'n_estimators': 30, 'learning_rate': 0.05, 'reg_lambda': 20.0, 'min_child_weight': 3}
XGBoost	15	24/12	IgM anti-FINC[959-984]	0.870	0.948	0.086	1.184/-0.277	{'max_depth': 2, 'n_estimators': 30, 'learning_rate': 0.05, 'reg_lambda': 20.0, 'min_child_weight': 3}
XGBoost	15	24/12	IgG anti-C4A/B[167-187]	2.027	0.948	0.086	1.184/-0.277	{'max_depth': 2, 'n_estimators': 30, 'learning_rate': 0.05, 'reg_lambda': 20.0, 'min_child_weight': 3}
XGBoost	16	24/12	IgG anti-ApoB-100[1662-1683] MDA	2.116	0.875	0.144	0.519/0.439	{'max_depth': 2, 'n_estimators': 30, 'learning_rate': 0.1, 'reg_lambda': 20.0, 'min_child_weight': 3}
XGBoost	16	24/12	IgM anti-FINC[959-984]	0.869	0.875	0.144	0.519/0.439	{'max_depth': 2, 'n_estimators': 30, 'learning_rate': 0.1, 'reg_lambda': 20.0, 'min_child_weight': 3}
XGBoost	16	24/12	IgG anti-C4A/B[167-187]	1.861	0.875	0.144	0.519/0.439	{'max_depth': 2, 'n_estimators': 30, 'learning_rate': 0.1, 'reg_lambda': 20.0, 'min_child_weight': 3}
XGBoost	17	24/12	IgG anti-ApoB-100[1662-1683] MDA	0.936	0.979	0.066	1.149/-0.232	{'max_depth': 2, 'n_estimators': 30, 'learning_rate': 0.05, 'reg_lambda': 20.0, 'min_child_weight': 3}

XGBoost	17	24/12	IgM anti-FINC$^{959-984}$	1.007	0.979	0.066	1.149/-0.232	{'max_depth': 2, 'n_estimators': 30, 'learning_rate': 0.05, 'reg_lambda': 20.0, 'min_child_weight': 3}
XGBoost	17	24/12	IgG anti-C4A/B$^{167-187}$	1.975	0.979	0.066	1.149/-0.232	{'max_depth': 2, 'n_estimators': 30, 'learning_rate': 0.05, 'reg_lambda': 20.0, 'min_child_weight': 3}
XGBoost	18	24/12	IgG anti-ApoB-100$^{1662-1683}$ MDA	1.196	0.861	0.126	0.545/0.018	{'max_depth': 2, 'n_estimators': 30, 'learning_rate': 0.1, 'reg_lambda': 20.0, 'min_child_weight': 3}
XGBoost	18	24/12	PCr	0.786	0.861	0.126	0.545/0.018	{'max_depth': 2, 'n_estimators': 30, 'learning_rate': 0.1, 'reg_lambda': 20.0, 'min_child_weight': 3}
XGBoost	18	24/12	IgG anti-C4A/B$^{167-187}$	2.110	0.861	0.126	0.545/0.018	{'max_depth': 2, 'n_estimators': 30, 'learning_rate': 0.1, 'reg_lambda': 20.0, 'min_child_weight': 3}
XGBoost	19	24/12	IgG anti-ApoB-100$^{1662-1683}$ MDA	0.848	1.000	0.022	10.196/9.508	{'max_depth': 2, 'n_estimators': 30, 'learning_rate': 0.05, 'reg_lambda': 20.0, 'min_child_weight': 3}
XGBoost	19	24/12	IgM anti-FINC$^{959-984}$	1.028	1.000	0.022	10.196/9.508	{'max_depth': 2, 'n_estimators': 30, 'learning_rate': 0.05, 'reg_lambda': 20.0, 'min_child_weight': 3}
XGBoost	19	24/12	IgG anti-C4A/B$^{167-187}$	1.734	1.000	0.022	10.196/9.508	{'max_depth': 2, 'n_estimators': 30, 'learning_rate': 0.05,

								'reg_lambda': 20.0, 'min_child_weight': 3}
XGBoost	20	24/12	IgG anti-ApoB-100$^{1662-1683}$ MDA	1.225	0.757	0.253	0.290/0.146	{'max_depth': 2, 'n_estimators': 30, 'learning_rate': 0.1, 'reg_lambda': 20.0, 'min_child_weight': 3}
XGBoost	20	24/12	MDA-protein adduct	1.484	0.757	0.253	0.290/0.146	{'max_depth': 2, 'n_estimators': 30, 'learning_rate': 0.1, 'reg_lambda': 20.0, 'min_child_weight': 3}
XGBoost	20	24/12	IgM anti-FINC$^{959-984}$	1.993	0.757	0.253	0.290/0.146	{'max_depth': 2, 'n_estimators': 30, 'learning_rate': 0.1, 'reg_lambda': 20.0, 'min_child_weight': 3}
XGBoost	21	24/12	IgG anti-ApoB-100$^{1662-1683}$ MDA	1.195	1.000	0.014	12.060/-0.990	{'max_depth': 2, 'n_estimators': 30, 'learning_rate': 0.1, 'reg_lambda': 20.0, 'min_child_weight': 3}
XGBoost	21	24/12	IgM anti-FINC$^{959-984}$	1.722	1.000	0.014	12.060/-0.990	{'max_depth': 2, 'n_estimators': 30, 'learning_rate': 0.1, 'reg_lambda': 20.0, 'min_child_weight': 3}
XGBoost	21	24/12	IgG anti-C4A/B$^{167-187}$	1.940	1.000	0.014	12.060/-0.990	{'max_depth': 2, 'n_estimators': 30, 'learning_rate': 0.1, 'reg_lambda': 20.0, 'min_child_weight': 3}
XGBoost	22	24/12	IgG anti-ApoB-100$^{1662-1683}$ MDA	1.376	0.938	0.137	0.843/0.982	{'max_depth': 2, 'n_estimators': 30, 'learning_rate': 0.1, 'reg_lambda': 20.0, 'min_child_weight': 3}

XGBoost	22	24/12	IgM anti-FINC959-984	1.093	0.938	0.137	0.843/0.982	{'max_depth': 2, 'n_estimators': 30, 'learning_rate': 0.1, 'reg_lambda': 20.0, 'min_child_weight': 3}
XGBoost	22	24/12	IgG anti-C4A/B167-187	2.044	0.938	0.137	0.843/0.982	{'max_depth': 2, 'n_estimators': 30, 'learning_rate': 0.1, 'reg_lambda': 20.0, 'min_child_weight': 3}
XGBoost	23	24/12	IgG anti-ApoB-1001662-1683 MDA	1.782	0.920	0.072	0.731/0.478	{'max_depth': 2, 'n_estimators': 30, 'learning_rate': 0.1, 'reg_lambda': 20.0, 'min_child_weight': 3}
XGBoost	23	24/12	IgG anti-C4A/B167-187	1.984	0.920	0.072	0.731/0.478	{'max_depth': 2, 'n_estimators': 30, 'learning_rate': 0.1, 'reg_lambda': 20.0, 'min_child_weight': 3}
XGBoost	23	24/12	CRI II	1.094	0.920	0.072	0.731/0.478	{'max_depth': 2, 'n_estimators': 30, 'learning_rate': 0.1, 'reg_lambda': 20.0, 'min_child_weight': 3}
XGBoost	24	24/12	IgG anti-ApoB-1001662-1683 MDA	1.535	0.854	0.169	0.667/0.341	{'max_depth': 2, 'n_estimators': 30, 'learning_rate': 0.1, 'reg_lambda': 20.0, 'min_child_weight': 3}
XGBoost	24	24/12	MDA-protein adduct	0.792	0.854	0.169	0.667/0.341	{'max_depth': 2, 'n_estimators': 30, 'learning_rate': 0.1, 'reg_lambda': 20.0, 'min_child_weight': 3}
XGBoost	24	24/12	CRI II	1.091	0.854	0.169	0.667/0.341	{'max_depth': 2, 'n_estimators': 30, 'learning_rate': 0.1,

								'reg_lambda': 20.0,<br>'min_child_weight': 3}
XGBoost	25	24/12	IgG anti-ApoB-100$^{1662-1683}$ MDA	1.104	0.931	0.149	0.790/-0.899	{'max_depth': 2,<br>'n_estimators': 30,<br>'learning_rate': 0.05,<br>'reg_lambda': 20.0,<br>'min_child_weight': 3}
XGBoost	25	24/12	IgM anti-FINC$^{959-984}$	1.063	0.931	0.149	0.790/-0.899	{'max_depth': 2,<br>'n_estimators': 30,<br>'learning_rate': 0.05,<br>'reg_lambda': 20.0,<br>'min_child_weight': 3}
XGBoost	25	24/12	IgG anti-C4A/B$^{167-187}$	1.835	0.931	0.149	0.790/-0.899	{'max_depth': 2,<br>'n_estimators': 30,<br>'learning_rate': 0.05,<br>'reg_lambda': 20.0,<br>'min_child_weight': 3}
LightGBM	1	24/12	IgG anti-ApoB-100$^{1662-1683}$ MDA	1.603	0.972	0.064	1.024/-0.202	{'max_depth': 2,<br>'n_estimators': 30,<br>'learning_rate': 0.1,<br>'reg_lambda': 20.0,<br>'min_child_samples': 20}
LightGBM	1	24/12	IgM anti-FINC$^{959-984}$	1.390	0.972	0.064	1.024/-0.202	{'max_depth': 2,<br>'n_estimators': 30,<br>'learning_rate': 0.1,<br>'reg_lambda': 20.0,<br>'min_child_samples': 20}
LightGBM	1	24/12	IgG anti-C4A/B$^{167-187}$	2.067	0.972	0.064	1.024/-0.202	{'max_depth': 2,<br>'n_estimators': 30,<br>'learning_rate': 0.1,<br>'reg_lambda': 20.0,<br>'min_child_samples': 20}
LightGBM	2	24/12	IgG anti-ApoB-100$^{1662-1683}$ MDA	1.491	0.816	0.258	0.242/-0.286	{'max_depth': 2,<br>'n_estimators': 30,<br>'learning_rate': 0.1,<br>'reg_lambda': 20.0,<br>'min_child_samples': 20}

LightGBM	2	24/12	IgM anti-FINC$^{959-984}$	3.363	0.816	0.258	0.242/-0.286	{'max_depth': 2, 'n_estimators': 30, 'learning_rate': 0.1, 'reg_lambda': 20.0, 'min_child_samples': 20}
LightGBM	2	24/12	IgG anti-C4A/B$^{167-187}$	3.055	0.816	0.258	0.242/-0.286	{'max_depth': 2, 'n_estimators': 30, 'learning_rate': 0.1, 'reg_lambda': 20.0, 'min_child_samples': 20}
LightGBM	3	24/12	IgG anti-ApoB-100$^{1662-1683}$ MDA	2.200	0.944	0.108	0.862/2.409	{'max_depth': 2, 'n_estimators': 30, 'learning_rate': 0.1, 'reg_lambda': 20.0, 'min_child_samples': 20}
LightGBM	3	24/12	IgM anti-FINC$^{959-984}$	1.296	0.944	0.108	0.862/2.409	{'max_depth': 2, 'n_estimators': 30, 'learning_rate': 0.1, 'reg_lambda': 20.0, 'min_child_samples': 20}
LightGBM	3	24/12	IgG anti-C4A/B$^{167-187}$	3.017	0.944	0.108	0.862/2.409	{'max_depth': 2, 'n_estimators': 30, 'learning_rate': 0.1, 'reg_lambda': 20.0, 'min_child_samples': 20}
LightGBM	4	24/12	IgG anti-ApoB-100$^{1662-1683}$ MDA	1.121	0.903	0.134	0.531/-0.303	{'max_depth': 2, 'n_estimators': 30, 'learning_rate': 0.1, 'reg_lambda': 20.0, 'min_child_samples': 20}
LightGBM	4	24/12	IgM anti-FINC$^{959-984}$	1.567	0.903	0.134	0.531/-0.303	{'max_depth': 2, 'n_estimators': 30, 'learning_rate': 0.1, 'reg_lambda': 20.0, 'min_child_samples': 20}
LightGBM	4	24/12	IgG anti-C4A/B$^{167-187}$	2.488	0.903	0.134	0.531/-0.303	{'max_depth': 2, 'n_estimators': 30, 'learning_rate': 0.1,

								'reg_lambda': 20.0,<br>'min_child_samples': 20}
LightGBM	5	24/12	IgG anti-ApoB-100$^{1662-1683}$<br>MDA	1.476	0.951	0.055	1.108/-0.772	{'max_depth': 2,<br>'n_estimators': 30,<br>'learning_rate': 0.1,<br>'reg_lambda': 20.0,<br>'min_child_samples': 20}
LightGBM	5	24/12	IgG anti-ApoB-100$^{1662-1683}$	0.310	0.951	0.055	1.108/-0.772	{'max_depth': 2,<br>'n_estimators': 30,<br>'learning_rate': 0.1,<br>'reg_lambda': 20.0,<br>'min_child_samples': 20}
LightGBM	5	24/12	IgG anti-C4A/B$^{167-187}$	1.672	0.951	0.055	1.108/-0.772	{'max_depth': 2,<br>'n_estimators': 30,<br>'learning_rate': 0.1,<br>'reg_lambda': 20.0,<br>'min_child_samples': 20}
LightGBM	6	24/12	IgG anti-ApoB-100$^{1662-1683}$<br>MDA	1.172	0.882	0.175	0.468/-0.284	{'max_depth': 2,<br>'n_estimators': 30,<br>'learning_rate': 0.1,<br>'reg_lambda': 20.0,<br>'min_child_samples': 20}
LightGBM	6	24/12	IgM anti-FINC$^{959-984}$	1.735	0.882	0.175	0.468/-0.284	{'max_depth': 2,<br>'n_estimators': 30,<br>'learning_rate': 0.1,<br>'reg_lambda': 20.0,<br>'min_child_samples': 20}
LightGBM	6	24/12	IgG anti-C4A/B$^{167-187}$	2.027	0.882	0.175	0.468/-0.284	{'max_depth': 2,<br>'n_estimators': 30,<br>'learning_rate': 0.1,<br>'reg_lambda': 20.0,<br>'min_child_samples': 20}
LightGBM	7	24/12	IgG anti-ApoB-100$^{1662-1683}$<br>MDA	1.711	0.937	0.119	0.683/0.267	{'max_depth': 2,<br>'n_estimators': 30,<br>'learning_rate': 0.1,<br>'reg_lambda': 20.0,<br>'min_child_samples': 20}

LightGBM	7	24/12	IgM anti-FINC$^{959-984}$	0.901	0.937	0.119	0.683/0.267	{'max_depth': 2, 'n_estimators': 30, 'learning_rate': 0.1, 'reg_lambda': 20.0, 'min_child_samples': 20}
LightGBM	7	24/12	IgG anti-C4A/B$^{167-187}$	2.334	0.937	0.119	0.683/0.267	{'max_depth': 2, 'n_estimators': 30, 'learning_rate': 0.1, 'reg_lambda': 20.0, 'min_child_samples': 20}
LightGBM	8	24/12	IgG anti-ApoB-100$^{1662-1683}$ MDA	1.574	0.840	0.161	0.634/0.091	{'max_depth': 2, 'n_estimators': 30, 'learning_rate': 0.1, 'reg_lambda': 20.0, 'min_child_samples': 20}
LightGBM	8	24/12	IgG anti-ApoB-100$^{1662-1683}$	0.711	0.840	0.161	0.634/0.091	{'max_depth': 2, 'n_estimators': 30, 'learning_rate': 0.1, 'reg_lambda': 20.0, 'min_child_samples': 20}
LightGBM	8	24/12	CRI II	1.456	0.840	0.161	0.634/0.091	{'max_depth': 2, 'n_estimators': 30, 'learning_rate': 0.1, 'reg_lambda': 20.0, 'min_child_samples': 20}
LightGBM	9	24/12	IgG anti-ApoB-100$^{1662-1683}$ MDA	1.909	0.958	0.082	0.663/0.903	{'max_depth': 2, 'n_estimators': 30, 'learning_rate': 0.1, 'reg_lambda': 20.0, 'min_child_samples': 20}
LightGBM	9	24/12	IgM anti-FINC$^{959-984}$	1.505	0.958	0.082	0.663/0.903	{'max_depth': 2, 'n_estimators': 30, 'learning_rate': 0.1, 'reg_lambda': 20.0, 'min_child_samples': 20}
LightGBM	9	24/12	IgG anti-C4A/B$^{167-187}$	2.409	0.958	0.082	0.663/0.903	{'max_depth': 2, 'n_estimators': 30, 'learning_rate': 0.1,

								'reg_lambda': 20.0, 'min_child_samples': 20}
LightGBM	10	24/12	IgG anti-ApoB-100$^{1662-1683}$ MDA	1.606	0.986	0.050	1.473/0.086	{'max_depth': 2, 'n_estimators': 30, 'learning_rate': 0.1, 'reg_lambda': 20.0, 'min_child_samples': 20}
LightGBM	10	24/12	IgM anti-FINC$^{959-984}$	1.642	0.986	0.050	1.473/0.086	{'max_depth': 2, 'n_estimators': 30, 'learning_rate': 0.1, 'reg_lambda': 20.0, 'min_child_samples': 20}
LightGBM	10	24/12	IgG anti-C4A/B$^{167-187}$	2.131	0.986	0.050	1.473/0.086	{'max_depth': 2, 'n_estimators': 30, 'learning_rate': 0.1, 'reg_lambda': 20.0, 'min_child_samples': 20}
LightGBM	11	24/12	IgG anti-ApoB-100$^{1662-1683}$ MDA	1.653	0.819	0.178	0.699/-0.024	{'max_depth': 2, 'n_estimators': 30, 'learning_rate': 0.1, 'reg_lambda': 20.0, 'min_child_samples': 20}
LightGBM	11	24/12	IgM anti-ApoB-100$^{1662-1683}$ MDA	0.804	0.819	0.178	0.699/-0.024	{'max_depth': 2, 'n_estimators': 30, 'learning_rate': 0.1, 'reg_lambda': 20.0, 'min_child_samples': 20}
LightGBM	11	24/12	CRI II	1.191	0.819	0.178	0.699/-0.024	{'max_depth': 2, 'n_estimators': 30, 'learning_rate': 0.1, 'reg_lambda': 20.0, 'min_child_samples': 20}
LightGBM	12	24/12	IgG anti-ApoB-100$^{1662-1683}$ MDA	1.071	0.938	0.085	0.744/-1.297	{'max_depth': 2, 'n_estimators': 30, 'learning_rate': 0.1, 'reg_lambda': 20.0, 'min_child_samples': 20}

LightGBM	12	24/12	IgM anti-FINC959-984	1.666	0.938	0.085	0.744/-1.297	{'max_depth': 2, 'n_estimators': 30, 'learning_rate': 0.1, 'reg_lambda': 20.0, 'min_child_samples': 20}
LightGBM	12	24/12	IgG anti-C4A/B167-187	2.400	0.938	0.085	0.744/-1.297	{'max_depth': 2, 'n_estimators': 30, 'learning_rate': 0.1, 'reg_lambda': 20.0, 'min_child_samples': 20}
LightGBM	13	24/12	IgG anti-ApoB-1001662-1683 MDA	1.373	0.986	0.080	1.391/1.843	{'max_depth': 2, 'n_estimators': 30, 'learning_rate': 0.1, 'reg_lambda': 20.0, 'min_child_samples': 20}
LightGBM	13	24/12	IgM anti-FINC959-984	1.524	0.986	0.080	1.391/1.843	{'max_depth': 2, 'n_estimators': 30, 'learning_rate': 0.1, 'reg_lambda': 20.0, 'min_child_samples': 20}
LightGBM	13	24/12	IgG anti-C4A/B167-187	2.529	0.986	0.080	1.391/1.843	{'max_depth': 2, 'n_estimators': 30, 'learning_rate': 0.1, 'reg_lambda': 20.0, 'min_child_samples': 20}
LightGBM	14	24/12	IgG anti-ApoB-1001662-1683 MDA	2.320	0.896	0.158	0.384/0.304	{'max_depth': 2, 'n_estimators': 30, 'learning_rate': 0.1, 'reg_lambda': 20.0, 'min_child_samples': 20}
LightGBM	14	24/12	IgM anti-FINC959-984	1.429	0.896	0.158	0.384/0.304	{'max_depth': 2, 'n_estimators': 30, 'learning_rate': 0.1, 'reg_lambda': 20.0, 'min_child_samples': 20}
LightGBM	14	24/12	IgG anti-C4A/B167-187	2.249	0.896	0.158	0.384/0.304	{'max_depth': 2, 'n_estimators': 30, 'learning_rate': 0.1,

								'reg_lambda': 20.0, 'min_child_samples': 20}
LightGBM	15	24/12	IgG anti-ApoB-100$^{1662-1683}$ MDA	1.710	0.858	0.144	0.587/-0.102	{'max_depth': 2, 'n_estimators': 30, 'learning_rate': 0.1, 'reg_lambda': 20.0, 'min_child_samples': 20}
LightGBM	15	24/12	IgG anti-C4A/B$^{167-187}$	2.219	0.858	0.144	0.587/-0.102	{'max_depth': 2, 'n_estimators': 30, 'learning_rate': 0.1, 'reg_lambda': 20.0, 'min_child_samples': 20}
LightGBM	15	24/12	Age	0.764	0.858	0.144	0.587/-0.102	{'max_depth': 2, 'n_estimators': 30, 'learning_rate': 0.1, 'reg_lambda': 20.0, 'min_child_samples': 20}
LightGBM	16	24/12	IgG anti-ApoB-100$^{1662-1683}$ MDA	2.771	0.861	0.158	0.396/0.482	{'max_depth': 2, 'n_estimators': 30, 'learning_rate': 0.1, 'reg_lambda': 20.0, 'min_child_samples': 20}
LightGBM	16	24/12	IgM anti-FINC$^{959-984}$	1.100	0.861	0.158	0.396/0.482	{'max_depth': 2, 'n_estimators': 30, 'learning_rate': 0.1, 'reg_lambda': 20.0, 'min_child_samples': 20}
LightGBM	16	24/12	IgG anti-C4A/B$^{167-187}$	2.473	0.861	0.158	0.396/0.482	{'max_depth': 2, 'n_estimators': 30, 'learning_rate': 0.1, 'reg_lambda': 20.0, 'min_child_samples': 20}
LightGBM	17	24/12	IgG anti-ApoB-100$^{1662-1683}$ MDA	1.676	0.958	0.063	1.764/-2.490	{'max_depth': 2, 'n_estimators': 30, 'learning_rate': 0.1, 'reg_lambda': 20.0, 'min_child_samples': 20}

LightGBM	17	24/12	IgG anti-C4A/B167-187	1.912	0.958	0.063	1.764/-2.490	{'max_depth': 2, 'n_estimators': 30, 'learning_rate': 0.1, 'reg_lambda': 20.0, 'min_child_samples': 20}
LightGBM	17	24/12	Age	0.607	0.958	0.063	1.764/-2.490	{'max_depth': 2, 'n_estimators': 30, 'learning_rate': 0.1, 'reg_lambda': 20.0, 'min_child_samples': 20}
LightGBM	18	24/12	IgG anti-ApoB-1001662-1683 MDA	1.630	0.927	0.047	0.685/0.240	{'max_depth': 2, 'n_estimators': 30, 'learning_rate': 0.1, 'reg_lambda': 20.0, 'min_child_samples': 20}
LightGBM	18	24/12	IgG anti-FINC959-984 MDA	1.108	0.927	0.047	0.685/0.240	{'max_depth': 2, 'n_estimators': 30, 'learning_rate': 0.1, 'reg_lambda': 20.0, 'min_child_samples': 20}
LightGBM	18	24/12	IgG anti-C4A/B167-187	2.315	0.927	0.047	0.685/0.240	{'max_depth': 2, 'n_estimators': 30, 'learning_rate': 0.1, 'reg_lambda': 20.0, 'min_child_samples': 20}
LightGBM	19	24/12	IgG anti-ApoB-1001662-1683 MDA	1.273	0.993	0.040	4.949/2.719	{'max_depth': 2, 'n_estimators': 30, 'learning_rate': 0.1, 'reg_lambda': 20.0, 'min_child_samples': 20}
LightGBM	19	24/12	IgM anti-FINC959-984	1.287	0.993	0.040	4.949/2.719	{'max_depth': 2, 'n_estimators': 30, 'learning_rate': 0.1, 'reg_lambda': 20.0, 'min_child_samples': 20}
LightGBM	19	24/12	IgG anti-C4A/B167-187	2.255	0.993	0.040	4.949/2.719	{'max_depth': 2, 'n_estimators': 30, 'learning_rate': 0.1,

								'reg_lambda': 20.0, 'min_child_samples': 20}
LightGBM	20	24/12	IgG anti-ApoB-100$^{1662-1683}$ MDA	1.822	0.868	0.158	0.390/0.231	{'max_depth': 2, 'n_estimators': 30, 'learning_rate': 0.1, 'reg_lambda': 20.0, 'min_child_samples': 20}
LightGBM	20	24/12	IgM anti-FINC$^{959-984}$	2.438	0.868	0.158	0.390/0.231	{'max_depth': 2, 'n_estimators': 30, 'learning_rate': 0.1, 'reg_lambda': 20.0, 'min_child_samples': 20}
LightGBM	20	24/12	IgG anti-C4A/B$^{167-187}$	2.695	0.868	0.158	0.390/0.231	{'max_depth': 2, 'n_estimators': 30, 'learning_rate': 0.1, 'reg_lambda': 20.0, 'min_child_samples': 20}
LightGBM	21	24/12	IgG anti-ApoB-100$^{1662-1683}$ MDA	1.658	0.976	0.069	3.498/1.946	{'max_depth': 2, 'n_estimators': 30, 'learning_rate': 0.1, 'reg_lambda': 20.0, 'min_child_samples': 20}
LightGBM	21	24/12	IgG anti-ApoB-100$^{1662-1683}$	0.437	0.976	0.069	3.498/1.946	{'max_depth': 2, 'n_estimators': 30, 'learning_rate': 0.1, 'reg_lambda': 20.0, 'min_child_samples': 20}
LightGBM	21	24/12	IgG anti-C4A/B$^{167-187}$	1.906	0.976	0.069	3.498/1.946	{'max_depth': 2, 'n_estimators': 30, 'learning_rate': 0.1, 'reg_lambda': 20.0, 'min_child_samples': 20}
LightGBM	22	24/12	IgG anti-ApoB-100$^{1662-1683}$ MDA	1.724	0.944	0.123	0.939/1.733	{'max_depth': 2, 'n_estimators': 30, 'learning_rate': 0.1, 'reg_lambda': 20.0, 'min_child_samples': 20}

LightGBM	22	24/12	IgM anti-FINC$^{959-984}$	1.174	0.944	0.123	0.939/1.733	{'max_depth': 2, 'n_estimators': 30, 'learning_rate': 0.1, 'reg_lambda': 20.0, 'min_child_samples': 20}
LightGBM	22	24/12	IgG anti-C4A/B$^{167-187}$	2.208	0.944	0.123	0.939/1.733	{'max_depth': 2, 'n_estimators': 30, 'learning_rate': 0.1, 'reg_lambda': 20.0, 'min_child_samples': 20}
LightGBM	23	24/12	IgG anti-ApoB-100$^{1662-1683}$ MDA	2.096	0.983	0.079	0.705/0.906	{'max_depth': 2, 'n_estimators': 30, 'learning_rate': 0.1, 'reg_lambda': 20.0, 'min_child_samples': 20}
LightGBM	23	24/12	IgM anti-FINC$^{959-984}$	1.436	0.983	0.079	0.705/0.906	{'max_depth': 2, 'n_estimators': 30, 'learning_rate': 0.1, 'reg_lambda': 20.0, 'min_child_samples': 20}
LightGBM	23	24/12	IgG anti-C4A/B$^{167-187}$	2.411	0.983	0.079	0.705/0.906	{'max_depth': 2, 'n_estimators': 30, 'learning_rate': 0.1, 'reg_lambda': 20.0, 'min_child_samples': 20}
LightGBM	24	24/12	IgG anti-ApoB-100$^{1662-1683}$ MDA	1.879	0.913	0.137	0.615/-0.574	{'max_depth': 2, 'n_estimators': 30, 'learning_rate': 0.1, 'reg_lambda': 20.0, 'min_child_samples': 20}
LightGBM	24	24/12	IgM anti-ApoB-100$^{1662-1683}$	1.001	0.913	0.137	0.615/-0.574	{'max_depth': 2, 'n_estimators': 30, 'learning_rate': 0.1, 'reg_lambda': 20.0, 'min_child_samples': 20}
LightGBM	24	24/12	IgG anti-C4A/B$^{167-187}$	2.193	0.913	0.137	0.615/-0.574	{'max_depth': 2, 'n_estimators': 30, 'learning_rate': 0.1,

								'reg_lambda': 20.0, 'min_child_samples': 20}
LightGBM	25	24/12	IgG anti-ApoB-100$^{1662-1683}$ MDA	1.857	0.882	0.183	0.413/-0.762	{'max_depth': 2, 'n_estimators': 30, 'learning_rate': 0.1, 'reg_lambda': 20.0, 'min_child_samples': 20}
LightGBM	25	24/12	IgM anti-FINC$^{959-984}$	1.796	0.882	0.183	0.413/-0.762	{'max_depth': 2, 'n_estimators': 30, 'learning_rate': 0.1, 'reg_lambda': 20.0, 'min_child_samples': 20}
LightGBM	25	24/12	IgG anti-C4A/B$^{167-187}$	2.704	0.882	0.183	0.413/-0.762	{'max_depth': 2, 'n_estimators': 30, 'learning_rate': 0.1, 'reg_lambda': 20.0, 'min_child_samples': 20}
CatBoost	1	24/12	IgG anti-ApoB-100$^{1662-1683}$ MDA	0.106	1.000	0.063	25.613/-10.997	{'iterations': 200, 'depth': 2, 'l2_leaf_reg': 20}
CatBoost	1	24/12	IgM anti-FINC$^{959-984}$	0.032	1.000	0.063	25.613/-10.997	{'iterations': 200, 'depth': 2, 'l2_leaf_reg': 20}
CatBoost	1	24/12	IgG anti-C4A/B$^{167-187}$	0.226	1.000	0.063	25.613/-10.997	{'iterations': 200, 'depth': 2, 'l2_leaf_reg': 20}
CatBoost	2	24/12	IgG anti-ApoB-100$^{1662-1683}$ MDA	0.169	0.743	0.239	0.350/0.093	{'iterations': 200, 'depth': 2, 'l2_leaf_reg': 20}
CatBoost	2	24/12	IgM anti-FINC$^{959-984}$	0.134	0.743	0.239	0.350/0.093	{'iterations': 200, 'depth': 2, 'l2_leaf_reg': 20}
CatBoost	2	24/12	IgG anti-C4A/B$^{167-187}$	0.162	0.743	0.239	0.350/0.093	{'iterations': 200, 'depth': 2, 'l2_leaf_reg': 20}
CatBoost	3	24/12	IgG anti-ApoB-100$^{1662-1683}$ MDA	0.131	0.819	0.179	0.742/0.275	{'iterations': 200, 'depth': 2, 'l2_leaf_reg': 20}
CatBoost	3	24/12	IgM anti-FINC$^{959-984}$	0.058	0.819	0.179	0.742/0.275	{'iterations': 200, 'depth': 2, 'l2_leaf_reg': 20}
CatBoost	3	24/12	IgG anti-C4A/B$^{167-187}$	0.176	0.819	0.179	0.742/0.275	{'iterations': 200, 'depth': 2, 'l2_leaf_reg': 20}
CatBoost	4	24/12	IgG anti-ApoB-100$^{1662-1683}$ MDA	0.109	0.861	0.138	0.592/-0.269	{'iterations': 200, 'depth': 2, 'l2_leaf_reg': 20}

CatBoost	4	24/12	IgM anti-FINC$^{959-984}$	0.037	0.861	0.138	0.592/-0.269	{'iterations': 200, 'depth': 2, 'l2_leaf_reg': 20}
CatBoost	4	24/12	IgG anti-C4A/B$^{167-187}$	0.260	0.861	0.138	0.592/-0.269	{'iterations': 200, 'depth': 2, 'l2_leaf_reg': 20}
CatBoost	5	24/12	IgG anti-ApoB-100$^{1662-1683}$ MDA	0.131	0.972	0.070	1.841/0.480	{'iterations': 200, 'depth': 2, 'l2_leaf_reg': 20}
CatBoost	5	24/12	PCr	0.050	0.972	0.070	1.841/0.480	{'iterations': 200, 'depth': 2, 'l2_leaf_reg': 20}
CatBoost	5	24/12	IgG anti-C4A/B$^{167-187}$	0.180	0.972	0.070	1.841/0.480	{'iterations': 200, 'depth': 2, 'l2_leaf_reg': 20}
CatBoost	6	24/12	IgG anti-ApoB-100$^{1662-1683}$ MDA	0.136	0.854	0.154	0.736/0.608	{'iterations': 200, 'depth': 2, 'l2_leaf_reg': 20}
CatBoost	6	24/12	IgM anti-FINC$^{959-984}$	0.078	0.854	0.154	0.736/0.608	{'iterations': 200, 'depth': 2, 'l2_leaf_reg': 20}
CatBoost	6	24/12	IgG anti-C4A/B$^{167-187}$	0.183	0.854	0.154	0.736/0.608	{'iterations': 200, 'depth': 2, 'l2_leaf_reg': 20}
CatBoost	7	24/12	IgG anti-ApoB-100$^{1662-1683}$ MDA	0.108	0.875	0.137	1.150/-0.720	{'iterations': 200, 'depth': 2, 'l2_leaf_reg': 20}
CatBoost	7	24/12	IgM anti-FINC$^{959-984}$	0.068	0.875	0.137	1.150/-0.720	{'iterations': 200, 'depth': 2, 'l2_leaf_reg': 20}
CatBoost	7	24/12	IgG anti-C4A/B$^{167-187}$	0.217	0.875	0.137	1.150/-0.720	{'iterations': 200, 'depth': 2, 'l2_leaf_reg': 20}
CatBoost	8	24/12	IgG anti-ApoB-100$^{1662-1683}$ MDA	0.134	0.944	0.107	1.175/-0.214	{'iterations': 200, 'depth': 2, 'l2_leaf_reg': 20}
CatBoost	8	24/12	IgM anti-FINC$^{959-984}$	0.058	0.944	0.107	1.175/-0.214	{'iterations': 200, 'depth': 2, 'l2_leaf_reg': 20}
CatBoost	8	24/12	IgG anti-C4A/B$^{167-187}$	0.225	0.944	0.107	1.175/-0.214	{'iterations': 200, 'depth': 2, 'l2_leaf_reg': 20}
CatBoost	9	24/12	IgG anti-ApoB-100$^{1662-1683}$ MDA	0.164	0.833	0.115	0.692/0.331	{'iterations': 200, 'depth': 2, 'l2_leaf_reg': 20}
CatBoost	9	24/12	IgM anti-FINC$^{959-984}$	0.048	0.833	0.115	0.692/0.331	{'iterations': 200, 'depth': 2, 'l2_leaf_reg': 20}
CatBoost	9	24/12	IgG anti-C4A/B$^{167-187}$	0.176	0.833	0.115	0.692/0.331	{'iterations': 200, 'depth': 2, 'l2_leaf_reg': 20}
CatBoost	10	24/12	IgG anti-ApoB-100$^{1662-1683}$ MDA	0.131	0.931	0.122	1.910/-0.600	{'iterations': 200, 'depth': 2, 'l2_leaf_reg': 20}
CatBoost	10	24/12	IgM anti-FINC$^{959-984}$	0.055	0.931	0.122	1.910/-0600	{'iterations': 200, 'depth': 2, 'l2_leaf_reg': 20}

CatBoost	10	24/12	IgG anti-C4A/B$^{167-187}$	0.216	0.931	0.122	1.910/-0.600	{'iterations': 200, 'depth': 2, 'l2_leaf_reg': 20}
CatBoost	11	24/12	IgG anti-ApoB-100$^{1662-1683}$ MDA	0.154	0.972	0.071	4.591/2.439	{'iterations': 200, 'depth': 2, 'l2_leaf_reg': 20}
CatBoost	11	24/12	IgM anti-FINC$^{959-984}$	0.053	0.972	0.071	4.591/2.439	{'iterations': 200, 'depth': 2, 'l2_leaf_reg': 20}
CatBoost	11	24/12	IgG anti-C4A/B$^{167-187}$	0.154	0.972	0.071	4.591/2.439	{'iterations': 200, 'depth': 2, 'l2_leaf_reg': 20}
CatBoost	12	24/12	IgG anti-ApoB-100$^{1662-1683}$ MDA	0.168	0.889	0.134	0.999/-0.106	{'iterations': 200, 'depth': 2, 'l2_leaf_reg': 20}
CatBoost	12	24/12	IgM anti-FINC$^{959-984}$	0.088	0.889	0.134	0.999/-0.106	{'iterations': 200, 'depth': 2, 'l2_leaf_reg': 20}
CatBoost	12	24/12	IgG anti-C4A/B$^{167-187}$	0.178	0.889	0.134	0.999/-0.106	{'iterations': 200, 'depth': 2, 'l2_leaf_reg': 20}
CatBoost	13	24/12	IgG anti-ApoB-100$^{1662-1683}$ MDA	0.114	0.917	0.113	1.628/-0.534	{'iterations': 200, 'depth': 2, 'l2_leaf_reg': 20}
CatBoost	13	24/12	IgM anti-C4A/B$^{167-187}$	0.016	0.917	0.113	1.628/-0.534	{'iterations': 200, 'depth': 2, 'l2_leaf_reg': 20}
CatBoost	13	24/12	IgG anti-C4A/B$^{167-187}$	0.226	0.917	0.113	1.628/-0.534	{'iterations': 200, 'depth': 2, 'l2_leaf_reg': 20}
CatBoost	14	24/12	IgG anti-APOB-100$^{1662-1683}$ MDA	0.149	0.785	0.188	0.395/-0.099	{'iterations': 200, 'depth': 2, 'l2_leaf_reg': 20}
CatBoost	14	24/12	IgM anti-FINC$^{959-984}$	0.082	0.785	0.188	0.395/-0.099	{'iterations': 200, 'depth': 2, 'l2_leaf_reg': 20}
CatBoost	14	24/12	IgG anti-C4A/B$^{167-187}$	0.221	0.785	0.188	0.395/-0.099	{'iterations': 200, 'depth': 2, 'l2_leaf_reg': 20}
CatBoost	15	24/12	IgG anti-ApoB-100$^{1662-1683}$ MDA	0.096	0.799	0.172	0.404/0.088	{'iterations': 200, 'depth': 2, 'l2_leaf_reg': 20}
CatBoost	15	24/12	IgM anti-FINC$^{959-984}$	0.034	0.799	0.172	0.404/0.088	{'iterations': 200, 'depth': 2, 'l2_leaf_reg': 20}
CatBoost	15	24/12	IgG anti-C4A/B$^{167-187}$	0.288	0.799	0.172	0.404/0.088	{'iterations': 200, 'depth': 2, 'l2_leaf_reg': 20}
CatBoost	16	24/12	IgG anti-ApoB-100$^{1662-1683}$ MDA	0.189	0.785	0.184	0.470/0.473	{'iterations': 200, 'depth': 2, 'l2_leaf_reg': 20}
CatBoost	16	24/12	IgM anti-FINC$^{959-984}$	0.072	0.785	0.184	0.470/0.473	{'iterations': 200, 'depth': 2, 'l2_leaf_reg': 20}
CatBoost	16	24/12	IgG anti-C4A/B$^{167-187}$	0.174	0.785	0.184	0.470/0.473	{'iterations': 200, 'depth': 2, 'l2_leaf_reg': 20}

CatBoost	17	24/12	IgG anti-ApoB-100$^{1662-1683}$ MDA	0.146	0.889	0.145	1.022/-0.537	{'iterations': 200, 'depth': 2, 'l2_leaf_reg': 20}
CatBoost	17	24/12	IgM anti-FINC$^{959-984}$	0.050	0.889	0.145	1.022/-0.537	{'iterations': 200, 'depth': 2, 'l2_leaf_reg': 20}
CatBoost	17	24/12	IgG anti-C4A/B$^{167-187}$	0.186	0.889	0.145	1.022/-0.537	{'iterations': 200, 'depth': 2, 'l2_leaf_reg': 20}
CatBoost	18	24/12	IgG anti-ApoB-100$^{1662-1683}$ MDA	0.117	0.965	0.107	2.116/-0.983	{'iterations': 200, 'depth': 2, 'l2_leaf_reg': 20}
CatBoost	18	24/12	IgM anti-FINC$^{959-984}$	0.061	0.965	0.107	2.116/-0.983	{'iterations': 200, 'depth': 2, 'l2_leaf_reg': 20}
CatBoost	18	24/12	IgG anti-C4A/B$^{167-187}$	0.154	0.965	0.107	2.116/-0.983	{'iterations': 200, 'depth': 2, 'l2_leaf_reg': 20}
CatBoost	19	24/12	IgG anti-ApoB-100$^{1662-1683}$ MDA	0.088	0.951	0.114	4.079/2.890	{'iterations': 200, 'depth': 2, 'l2_leaf_reg': 20}
CatBoost	19	24/12	IgM anti-FINC$^{959-984}$	0.064	0.951	0.114	4.079/2.890	{'iterations': 200, 'depth': 2, 'l2_leaf_reg': 20}
CatBoost	19	24/12	IgG anti-C4A/B$^{167-187}$	0.205	0.951	0.114	4.079/2.890	{'iterations': 200, 'depth': 2, 'l2_leaf_reg': 20}
CatBoost	20	24/12	IgG anti-ApoB-100$^{1662-1683}$ MDA	0.128	0.917	0.103	0.936/-0.355	{'iterations': 200, 'depth': 2, 'l2_leaf_reg': 20}
CatBoost	20	24/12	IgM anti-FINC$^{959-984}$	0.046	0.917	0.103	0.936/-0.355	{'iterations': 200, 'depth': 2, 'l2_leaf_reg': 20}
CatBoost	20	24/12	IgG anti-C4A/B$^{167-187}$	0.277	0.917	0.103	0.936/-0.355	{'iterations': 200, 'depth': 2, 'l2_leaf_reg': 20}
CatBoost	21	24/12	IgG anti-ApoB-100$^{1662-1683}$ MDA	0.130	0.986	0.090	3.176/0.416	{'iterations': 200, 'depth': 2, 'l2_leaf_reg': 20}
CatBoost	21	24/12	IgM anti-FINC$^{959-984}$	0.048	0.986	0.090	3.1760.416	{'iterations': 200, 'depth': 2, 'l2_leaf_reg': 20}
CatBoost	21	24/12	IgG anti-C4A/B$^{167-187}$	0.185	0.986	0.090	3.176/0.416	{'iterations': 200, 'depth': 2, 'l2_leaf_reg': 20}
CatBoost	22	24/12	IgG anti-ApoB-100$^{1662-1683}$ MDA	0.122	0.910	0.143	1.193/1.005	{'iterations': 200, 'depth': 2, 'l2_leaf_reg': 20}
CatBoost	22	24/12	IgM anti-FINC$^{959-984}$	0.071	0.910	0.143	1.193/1.005	{'iterations': 200, 'depth': 2, 'l2_leaf_reg': 20}
CatBoost	22	24/12	IgG anti-C4A/B$^{167-187}$	0.169	0.910	0.143	1.193/1.005	{'iterations': 200, 'depth': 2, 'l2_leaf_reg': 20}
CatBoost	23	24/12	IgG anti-ApoB-100$^{1662-1683}$ MDA	0.182	0.896	0.107	0.878/0.168	{'iterations': 200, 'depth': 2, 'l2_leaf_reg': 20}

CatBoost	23	24/12	IgM anti-FINC$^{959-984}$	0.049	0.896	0.107	0.878/0.168	{'iterations': 200, 'depth': 2, 'l2_leaf_reg': 20}
CatBoost	23	24/12	IgG anti-C4A/B$^{167-187}$	0.196	0.896	0.107	0.878/0.168	{'iterations': 200, 'depth': 2, 'l2_leaf_reg': 20}
CatBoost	24	24/12	IgG anti-ApoB-100$^{1662-1683}$ MDA	0.171	0.819	0.207	0.582/-0.336	{'iterations': 200, 'depth': 2, 'l2_leaf_reg': 20}
CatBoost	24	24/12	IgM anti-FINC$^{959-984}$	0.085	0.819	0.207	0.582/-0.336	{'iterations': 200, 'depth': 2, 'l2_leaf_reg': 20}
CatBoost	24	24/12	IgG anti-C4A/B$^{167-187}$	0.143	0.819	0.207	0.582/-0.336	{'iterations': 200, 'depth': 2, 'l2_leaf_reg': 20}
CatBoost	25	24/12	IgG anti-ApoB-100$^{1662-1683}$ MDA	0.060	0.875	0.130	0.504/-0.435	{'iterations': 200, 'depth': 2, 'l2_leaf_reg': 20}
CatBoost	25	24/12	IgM anti-FINC$^{959-984}$	0.058	0.875	0.130	0.504/-0.435	{'iterations': 200, 'depth': 2, 'l2_leaf_reg': 20}
CatBoost	25	24/12	IgG anti-C4A/B$^{167-187}$	0.326	0.875	0.130	0.504/-0.435	{'iterations': 200, 'depth': 2, 'l2_leaf_reg': 20}
LR	1	24/12	IgG anti-ApoB-100$^{1662-1683}$ MDA	0.796	0.896	0.117	1.740/-0.144	{'C': 1.0, 'l1_ratio': 0.5, 'penalty': 'l2'}
LR	1	24/12	Age	0.528	0.896	0.117	1.740/-0.144	{'C': 1.0, 'l1_ratio': 0.5, 'penalty': 'l2'}
LR	1	24/12	CRI II	0.574	0.896	0.117	1.740/-0.144	{'C': 1.0, 'l1_ratio': 0.5, 'penalty': 'l2'}
LR	2	24/12	IgG anti-ApoB-100$^{1662-1683}$ MDA	1.678	0.743	0.218	0.232/0.062	{'C': 1.0, 'l1_ratio': 0.5, 'penalty': 'l2'}
LR	2	24/12	MDA-protein adduct	1.263	0.743	0.218	0.232/0.062	{'C': 1.0, 'l1_ratio': 0.5, 'penalty': 'l2'}
LR	2	24/12	CRI II	0.301	0.743	0.218	0.232/0.062	{'C': 1.0, 'l1_ratio': 0.5, 'penalty': 'l2'}
LR	3	24/12	IgG anti-ApoB-100$^{1662-1683}$ MDA	0.824	0.785	0.184	0.737/0.320	{'C': 1.0, 'l1_ratio': 0.5, 'penalty': 'l2'}
LR	3	24/12	Age	0.702	0.785	0.184	0.737/0.320	{'C': 1.0, 'l1_ratio': 0.5, 'penalty': 'l2'}
LR	3	24/12	CRI II	0.588	0.785	0.184	0.737/0.320	{'C': 1.0, 'l1_ratio': 0.5, 'penalty': 'l2'}
LR	4	24/12	IgG anti-ApoB-100$^{1662-1683}$ MDA	0.927	0.840	0.171	0.899/-0.428	{'C': 1.0, 'l1_ratio': 0.5, 'penalty': 'l2'}
LR	4	24/12	Age	0.876	0.840	0.171	0.899/-0.428	{'C': 1.0, 'l1_ratio': 0.5, 'penalty': 'l2'}

LR	4	24/12	CRI II	0.614	0.840	0.171	0.899/-0.428	{'C': 1.0, 'l1_ratio': 0.5, 'penalty': 'l2'}
LR	5	24/12	IgG anti-ApoB-100$^{1662-1683}$ MDA	0.781	0.868	0.140	0.910/0.070	{'C': 1.0, 'l1_ratio': 0.5, 'penalty': 'l2'}
LR	5	24/12	Age	0.566	0.868	0.140	0.910/0.070	{'C': 1.0, 'l1_ratio': 0.5, 'penalty': 'l2'}
LR	5	24/12	CRI II	0.845	0.868	0.140	0.910/0.070	{'C': 1.0, 'l1_ratio': 0.5, 'penalty': 'l2'}
LR	6	24/12	IgG anti-ApoB-100$^{1662-1683}$ MDA	1.100	0.826	0.168	0.706/0.136	{'C': 1.0, 'l1_ratio': 0.5, 'penalty': 'l2'}
LR	6	24/12	Age	0.898	0.826	0.168	0.706/0.136	{'C': 1.0, 'l1_ratio': 0.5, 'penalty': 'l2'}
LR	6	24/12	CRI II	0.789	0.826	0.168	0.706/0.136	{'C': 1.0, 'l1_ratio': 0.5, 'penalty': 'l2'}
LR	7	24/12	IgG anti-ApoB-100$^{1662-1683}$ MDA	0.783	0.910	0.125	1.678/0.490	{'C': 1.0, 'l1_ratio': 0.5, 'penalty': 'l2'}
LR	7	24/12	Age	0.692	0.910	0.125	1.678/0.490	{'C': 1.0, 'l1_ratio': 0.5, 'penalty': 'l2'}
LR	7	24/12	CRI II	0.651	0.910	0.125	1.678/0.490	{'C': 1.0, 'l1_ratio': 0.5, 'penalty': 'l2'}
LR	8	24/12	IgG anti-ApoB-100$^{1662-1683}$ MDA	0.909	0.847	0.156	1.376/-0.132	{'C': 1.0, 'l1_ratio': 0.5, 'penalty': 'l2'}
LR	8	24/12	Age	0.527	0.847	0.156	1.376/-0.132	{'C': 1.0, 'l1_ratio': 0.5, 'penalty': 'l2'}
LR	8	24/12	CRI II	0.587	0.847	0.156	1.376/-0.132	{'C': 1.0, 'l1_ratio': 0.5, 'penalty': 'l2'}
LR	9	24/12	IgG anti-ApoB-100$^{1662-1683}$ MDA	1.084	0.833	0.142	0.898/0.100	{'C': 1.0, 'l1_ratio': 0.5, 'penalty': 'l2'}
LR	9	24/12	Age	0.722	0.833	0.142	0.898/0.100	{'C': 1.0, 'l1_ratio': 0.5, 'penalty': 'l2'}
LR	9	24/12	CRI II	0.480	0.833	0.142	0.898/0.100	{'C': 1.0, 'l1_ratio': 0.5, 'penalty': 'l2'}
LR	10	24/12	IgG anti-ApoB-100$^{1662-1683}$ MDA	0.970	0.861	0.162	0.939/-0.425	{'C': 1.0, 'l1_ratio': 0.5, 'penalty': 'l2'}
LR	10	24/12	Age	0.763	0.861	0.162	0.939/-0.425	{'C': 1.0, 'l1_ratio': 0.5, 'penalty': 'l2'}
LR	10	24/12	CRI II	0.584	0.861	0.162	0.939/-0.425	{'C': 1.0, 'l1_ratio': 0.5, 'penalty': 'l2'}

LR	11	24/12	IgG anti-ApoB-100$^{1662-1683}$ MDA	0.877	0.875	0.143	1.825/0.537	{'C': 1.0, 'l1_ratio': 0.5, 'penalty': 'l2'}
LR	11	24/12	Age	0.781	0.875	0.143	1.825/0.537	{'C': 1.0, 'l1_ratio': 0.5, 'penalty': 'l2'}
LR	11	24/12	CRI II	0.471	0.875	0.143	1.825/0.537	{'C': 1.0, 'l1_ratio': 0.5, 'penalty': 'l2'}
LR	12	24/12	IgG anti-ApoB-100$^{1662-1683}$ MDA	1.134	0.819	0.192	0.526/-0.169	{'C': 1.0, 'l1_ratio': 0.5, 'penalty': 'l2'}
LR	12	24/12	Age	0.978	0.819	0.192	0.526/-0.169	{'C': 1.0, 'l1_ratio': 0.5, 'penalty': 'l2'}
LR	12	24/12	CRI II	0.861	0.819	0.192	0.526/-0.169	{'C': 1.0, 'l1_ratio': 0.5, 'penalty': 'l2'}
LR	13	24/12	IgG anti-ApoB-100$^{1662-1683}$ MDA	0.704	0.944	0.115	2.201/-0.295	{'C': 1.0, 'l1_ratio': 0.5, 'penalty': 'l2'}
LR	13	24/12	Age	0.515	0.944	0.115	2.201/-0.295	{'C': 1.0, 'l1_ratio': 0.5, 'penalty': 'l2'}
LR	13	24/12	CRI II	0.554	0.944	0.115	2.201/-0.295	{'C': 1.0, 'l1_ratio': 0.5, 'penalty': 'l2'}
LR	14	24/12	IgG anti-ApoB-100$^{1662-1683}$ MDA	1.060	0.743	0.198	0.661/-0.042	{'C': 1.0, 'l1_ratio': 0.5, 'penalty': 'l2'}
LR	14	24/12	Age	0.666	0.743	0.198	0.661/-0.042	{'C': 1.0, 'l1_ratio': 0.5, 'penalty': 'l2'}
LR	14	24/12	CRI II	0.717	0.743	0.198	0.661/-0.042	{'C': 1.0, 'l1_ratio': 0.5, 'penalty': 'l2'}
LR	15	24/12	IgG anti-ApoB-100$^{1662-1683}$ MDA	1.141	0.896	0.127	1.143/0.039	{'C': 1.0, 'l1_ratio': 0.5, 'penalty': 'l2'}
LR	15	24/12	Age	0.591	0.896	0.127	1.143/0.039	{'C': 1.0, 'l1_ratio': 0.5, 'penalty': 'l2'}
LR	15	24/12	CRI II	0.524	0.896	0.127	1.143/0.039	{'C': 1.0, 'l1_ratio': 0.5, 'penalty': 'l2'}
LR	16	24/12	IgG anti-ApoB-100$^{1662-1683}$ MDA	1.349	0.785	0.186	0.770/0.621	{'C': 1.0, 'l1_ratio': 0.5, 'penalty': 'l2'}
LR	16	24/12	Age	0.683	0.785	0.186	0.770/0.621	{'C': 1.0, 'l1_ratio': 0.5, 'penalty': 'l2'}
LR	16	24/12	CRI II	0.307	0.785	0.186	0.770/0.621	{'C': 1.0, 'l1_ratio': 0.5, 'penalty': 'l2'}
LR	17	24/12	IgG anti-ApoB-100$^{1662-1683}$ MDA	1.064	0.868	0.142	0.802/0.041	{'C': 1.0, 'l1_ratio': 0.5, 'penalty': 'l2'}

LR	17	24/12	Age	0.802	0.868	0.142	0.802/0.041	{'C': 1.0, 'l1_ratio': 0.5, 'penalty': 'l2'}
LR	17	24/12	CRI II	0.581	0.868	0.142	0.802/0.041	{'C': 1.0, 'l1_ratio': 0.5, 'penalty': 'l2'}
LR	18	24/12	IgG anti-ApoB-100$^{1662-1683}$ MDA	0.668	0.868	0.157	1.047/-0.241	{'C': 1.0, 'l1_ratio': 0.5, 'penalty': 'l2'}
LR	18	24/12	Age	0.537	0.868	0.157	1.047/-0.241	{'C': 1.0, 'l1_ratio': 0.5, 'penalty': 'l2'}
LR	18	24/12	CRI II	0.834	0.868	0.157	1.047/-0.241	{'C': 1.0, 'l1_ratio': 0.5, 'penalty': 'l2'}
LR	19	24/12	IgG anti-ApoB-100$^{1662-1683}$ MDA	0.702	0.833	0.163	0.767/-0.186	{'C': 1.0, 'l1_ratio': 0.5, 'penalty': 'l2'}
LR	19	24/12	Age	0.791	0.833	0.163	0.767/-0.186	{'C': 1.0, 'l1_ratio': 0.5, 'penalty': 'l2'}
LR	19	24/12	CRI II	1.084	0.833	0.163	0.767/-0.186	{'C': 1.0, 'l1_ratio': 0.5, 'penalty': 'l2'}
LR	20	24/12	IgG anti-ApoB-100$^{1662-1683}$ MDA	0.889	0.903	0.121	1.115/-0.477	{'C': 1.0, 'l1_ratio': 0.5, 'penalty': 'l2'}
LR	20	24/12	MDA-protein adduct	1.046	0.903	0.121	1.115/-0.477	{'C': 1.0, 'l1_ratio': 0.5, 'penalty': 'l2'}
LR	20	24/12	CRI II	0.374	0.903	0.121	1.115/-0.477	{'C': 1.0, 'l1_ratio': 0.5, 'penalty': 'l2'}
LR	21	24/12	IgG anti-ApoB-100$^{1662-1683}$ MDA	0.621	0.931	0.153	1.662/-1.770	{'C': 1.0, 'l1_ratio': 0.5, 'penalty': 'l2'}
LR	21	24/12	MDA-protein adduct	1.282	0.931	0.153	1.662/-1.770	{'C': 1.0, 'l1_ratio': 0.5, 'penalty': 'l2'}
LR	21	24/12	CRI II	0.327	0.931	0.153	1.662/-1.770	{'C': 1.0, 'l1_ratio': 0.5, 'penalty': 'l2'}
LR	22	24/12	IgG anti-ApoB-100$^{1662-1683}$ MDA	0.857	0.882	0.138	1.249/0.076	{'C': 1.0, 'l1_ratio': 0.5, 'penalty': 'l2'}
LR	22	24/12	Age	0.703	0.882	0.138	1.249/0.076	{'C': 1.0, 'l1_ratio': 0.5, 'penalty': 'l2'}
LR	22	24/12	CRI II	0.579	0.882	0.138	1.249/0.076	{'C': 1.0, 'l1_ratio': 0.5, 'penalty': 'l2'}
LR	23	24/12	IgG anti-ApoB-100$^{1662-1683}$ MDA	1.289	0.861	0.144	0.814/0.346	{'C': 1.0, 'l1_ratio': 0.5, 'penalty': 'l2'}
LR	23	24/12	Age	0.743	0.861	0.144	0.814/0.346	{'C': 1.0, 'l1_ratio': 0.5, 'penalty': 'l2'}

LR	23	24/12	CRI II	0.706	0.861	0.144	0.814/0.346	{'C': 1.0, 'l1_ratio': 0.5, 'penalty': 'l2'}
LR	24	24/12	IgG anti-ApoB-100$^{1662-1683}$ MDA	1.312	0.792	0.246	0.424/-0.230	{'C': 1.0, 'l1_ratio': 0.5, 'penalty': 'l2'}
LR	24	24/12	Age	0.928	0.792	0.246	0.424/-0.230	{'C': 1.0, 'l1_ratio': 0.5, 'penalty': 'l2'}
LR	24	24/12	CRI II	0.459	0.792	0.246	0.424/-0.230	{'C': 1.0, 'l1_ratio': 0.5, 'penalty': 'l2'}
LR	25	24/12	IgG anti-ApoB-100$^{1662-1683}$ MDA	0.749	0.889	0.142	0.950/0.037	{'C': 1.0, 'l1_ratio': 0.5, 'penalty': 'l2'}
LR	25	24/12	Age	0.650	0.889	0.142	0.950/0.037	{'C': 1.0, 'l1_ratio': 0.5, 'penalty': 'l2'}
LR	25	24/12	CRI II	1.030	0.889	0.142	0.950/0.037	{'C': 1.0, 'l1_ratio': 0.5, 'penalty': 'l2'}

a. LightGBM, Light Gradient Boosting Machine; CatBoost, Categorical Boosting; XGBoost: eXtreme Gradient Boosting; RF, Random Forest; LR, Logistic Regression.

b. MDA, malondialdehyde; MDA-protein adduct, malondialdehyde-modified protein adduct; ApoB-100, apolipoprotein B-100; FINC, fibronectin; C4A/C4B, complement C4A/complement C4B; PCr, Plasma creatinine; TyG index, Triglyceride-glucose index was calculated as the natural logarithm (ln) of [fasting triglycerides (mg/dL) × fasting glucose (mg/dL)/2]; HbA1c/HDL-c ratio, The ratio of glycated hemoglobin (%) to high-density lipoprotein cholesterol (mg/dL); CRI I, Castelli risk index I was calculated by dividing total cholesterol by high-density lipoprotein cholesterol (TC/HDL-C); CRI II, Castelli risk index II was determined as the ratio of low-density lipoprotein cholesterol to high-density lipoprotein cholesterol (LDL-C/HDL-C); T2DM, type 2 diabetes mellitus; T2DM/CAD <70% stenosis, T2DM patients with non-obstructive CAD; T2DM/CAD ≥70% stenosis, T2DM patients with obstructive CAD.

c. SHAP, Shapley additive explanations.

Table S7. Predictive performance and fairness assessment across subgroups using a calibrated CatBoost and LR

Characteristic	n	Pooled AUC (95% CI) [a]		Brier score (95% CI) [a]		NB at 20% (95% CI) [a]	
		CatBoost	LR	CatBoost	LR	CatBoost	LR
Overall	165	0.975 (0.952, 0.993)	0.922‡** (0.875, 0.960)	0.069 (0.051, 0.088)	0.149‡*** (0.133, 0.168)	0.586 (0.508, 0.667)	0.545‡** (0.455, 0.636)
Male	108	0.974	0.900‡**	0.076	0.158‡***	0.583	0.537‡**

		(0.944, 0.992)	(0.832, 0.953)	(0.055, 0.100)	(0.135, 0.182)	(0.474, 0.678)	(0.410, 0.641)
Female	57	0.968 (0.905, 1.000)	0.957 (0.898, 0.996)	0.057 (0.026, 0.097)	$0.134^{\ddagger***}$ (0.110, 0.161)	0.592 (0.438, 0.732)	0.561 (0.408, 0.715)
Age ≥65 years	79	0.992 (0.975, 1.000)	0.952 (0.894, 0.996)	0.046 (0.028, 0.067)	$0.121^{\ddagger***}$ (0.099, 0.144)	0.731 (0.617, 0.832)	$0.699^{\ddagger**}$ (0.573, 0.810)
Age <65 years	86	0.952 (0.901, 0.989)	0.891 (0.819, 0.954)	$0.091^{\S*}$ (0.062, 0.123)	$0.175^{\ddagger***\$**}$ (0.149, 0.204)	$0.453^{\S*}$ (0.337, 0.576)	$0.404^{\ddagger**\$**}$ (0.273, 0.535)

a. CatBoost, Categorical Boosting; LR, Logistic Regression; Pooled AUC, Pooled area under the receiver operating characteristic curve (AUC) from out-of-fold (OOF) predictions**;** NB at 20%, Net benefit at a 20% threshold probability; CI, Confidence interval. $p < 0.05$ was used to determine statistical significance in comparisons between the two groups. ‡ CatBoost vs. LR; † CatBoost: Male vs. Female; ! LR: Male vs. Female;§ CatBoost: Age ≥65 years vs. Age <65 years; $ LR: Age ≥65 years vs. Age <65 years. * $p < 0.05$; ** $p < 0.01$; *** $p < 0.001$. The *p* values for performance disparity were calculated using 1,000 bootstrap resamples. These results should be interpreted with caution, as they may be influenced by the specific sample density of this cohort.

Supplementary Methods

1.1 Using 10% (w/v) trichloroacetic acid (TCA) in an acetone solution to remove plasma albumin

Plasma proteins were isolated using TCA-acetone precipitation, following the Poltep method.[2] Twenty microliters of pooled plasma was mixed with 80 µL of 10% (w/v) TCA in an acetone solution (ratio of plasma to solution 1:5) and incubated at -20 °C for 90 min. Using a centrifuge at 15,000 *g* and 4 °C for 10 min, the supernatant (albumin-enrich) was removed from the solution. The pellet (depleted of albumin) was washed with 200 µL of acetone and centrifuged twice at 15,000 *g* and 4 °C for 10 min. Then, the pellet was resuspended with 50 µL of 8 M urea. Plasma proteins were quantified using a Pierce Coomassie Plus (Bradford) Assay Kit (ThermoFisher Scientific, Waltham, MA, USA). Briefly, 4 µL of a protein sample or bovine serum albumin (BSA) standard solution was mixed with 200 µL Coomassie protein assay

reagent in the dark for 10 min, and the absorbance was measured at 595 nm. The protein concentration was measured using a BSA standard curve.[2]

1.2 In-solution digestion

Three micrograms of plasma protein was digested in-solution using a modified Glatter protocol.[3] In the denaturing and reduction step, 10 mM dithiothreitol (DTT) and 3 μg of protein in 8 M urea were prepared and reacted at 37 °C for 1 h. In the alkylation step, iodoacetamide was added to the reaction solution to 25 mM and reacted in the dark for 1 h. Then, DTT was added until it reached 25 mM to stop the reaction. In the enzyme digestion step, 25 mM ammonium bicarbonate buffer was added to the reaction solution to decrease the urea concentration to 4 M, and then 0.15 μg of Lys-C was added to react at 37 °C for 3 h. After that, 25 mM ammonium bicarbonate buffer was added to the reaction solution to decrease the urea concentration to <1 M. Subsequently, 100 ng of modified trypsin (Promega, Mannheim, Germany) in 25 mM NH_4HCO_3 was added. The sample was then incubated overnight at 37 °C for tryptic digestion. The tryptic peptide mixture was dried in a Speed-Vac (Thermo Fisher Scientific) and stored at −20 °C. The tryptic peptide mixture was suspended in 0.1% formic acid before use.

1.3 Identification of malondialdehyde (MDA)-modified peptide adducts through nano-liquid chromatography-tandem mass spectroscopy (LC-MS/MS) and PEAKS 7 software

The tryptic peptide mixture was identified in triplicate with a nanoLC-nanoESi-MS/MS system on a nanoAcquity system (Waters, Milford, MA, USA) connected to an LTQ-Orbitrap Elite hybrid mass spectrometer (Thermo Fisher Scientific, Bremen,

Germany) equipped with a PicoView nanospray interface (New Objective, Woburn, MA, USA). A 75-μm inner diameter, 25-cm-long C18 BEH column (Waters) was packed with 1.7-μm particles with a pore width of 130 Å which were separated using a segmented gradient for 30 min from 5% to 35% solvent B (acetonitrile with 0.1% formic acid) at a flow rate of 300 nl/min and a column temperature of 35 °C. Solvent A was 0.1% formic acid in water. The mass spectrometer was operated in the data-dependent mode. Briefly, survey full-scan MS spectra were acquired in the orbitrap (*m/z* 350–1600) with the resolution set to 120K at *m/z* 400 and an automatic gain control (AGC) target of 10^6. The 20 most intense ions were sequentially isolated for charge-injected device (CID) MS/MS fragmentation and detection in a linear ion trap (AGC target at 10,000) with previously selected ions dynamically excluded for 60 s. Ions with single and unrecognized charge states were also excluded.

The MS dataset of protein identification was analyzed by automated de novo sequencing conducted using PEAKS 7 software (Bioinformatics Solutions, Waterloo, Canada). A homology search compared de novo sequence tags against a human protein database of the Universal Protein Resource Knowledgebase (UniProt; http://www.uniprot.org/) with 159,628 entries (UniProt, 2017/07). PEAKS 7 uses an expectation-maximization (EM)-based algorithm for feature detection, deconvolution, and refinement. An optimization model for simultaneous feature matching and retention time alignment was used in the analysis. MDA-peptide and MDA-acetaldehyde-modified peptide (MAA-peptide) sequences and sites were identified using the PeaksPTM module of PEAKS 7 software. Carbamidomethylation (C)/+57.02 Da was set as the fixed modification, and oxidation (M)/+15.99 Da was selected as the variable modification. MDA modification (KHRQNatN)/+54.010567 Da) and MAA modification (K/+134.13 Da) were selected as the variable

modifications. The maximum missed cleavage was set to 2. The mass tolerance of precursor ions was set to 10 ppm, and the fragment ion tolerance with a monoisotopic mass was set to 0.6 Da. Using a false detection rate (FDR) of 1.0% and a significant score (−10lgP) for peptides of ≥22, proteins with a target-decoy database search were accepted if at least two peptides and two unique peptides were detected. Maximum variable post-translation modifications (PTMs) were set to 5. Furthermore, all modified MS spectra were manually identified in this study, and fragmented ions (from the nano-LC/MS/MS) were labeled as b, y, y-NH_3, and b-H_2O ions.

1.4 Immunoprecipitation (IP) and Western blotting (WB)

Sodium dodecylsulfate polyacrylamide gel electrophoresis (SDS-PAGE) was conducted using Laemmli's protocol.[4] MDA-modified proteins (MDA-proteins) were immunoprecipitated from pooled and individual plasma samples. Pooled plasma proteins were derived from 200 μg of pooled TCA-acetone-purified plasma using 29 pairs of patients with T2DM, T2DM/CAD <70% stenosis, and T2DM/CAD ≥70% stenosis. A pair of 200 μg of IgG-depleted plasma samples of individuals randomly selected from T2DM, T2DM/CAD <70% stenosis, and T2DM/CAD ≥70% stenosis patients was used as controls. Two micrograms of rabbit polyclonal anti-ApoB-100 (PAA603Hu01, Cloud-Clone, Katy, TX, USA), a rabbit polyclonal anti-FINC (EZ0439-CA, Boster Biological Technology, Pleasanton, CA, USA), or a rabbit monoclonal anti-C4A/C4B(E6J7W, Cell Signaling Technology, Danvers, MA, USA) was mixed with 2 mg of Protein A Sepharose CL-4B (GE Healthcare Bio-Sciences, Pittsburgh, PA, USA) in 400 μL of NET-2 buffer (150 mM NaCl, 0.5% Triton X100, and 50 mM Tris-HCl; pH 7.4) then incubated with 200 μg of pooled TCA-acetone-purified plasma or 200 μg of IgG-removal plasma proteins, and stirred gently for 2 h

at 4 °C. Beads were washed three times with 1 mL of NET-2* buffer (150 mM NaCl, 0.05% Triton X100, and 50 mM Tris-HCl; pH 7.4) and centrifuged at 4000 rpm for 3 min. Then, 20 μg of immunoprecipitated proteins loaded into an 8% SDS-PAGE gel were subjected to WB. Finally, MDA modification of the immunoprecipitated protein was verified through WB using a rabbit polyclonal anti-MDA antibody (STA-032; Cell Biolabs, San Diego, CA, USA), followed by horseradish peroxidase (HRP)-conjugated donkey anti-rabbit IgG (BioLegend, San Diego, CA, USA). Reactive protein bands were visualized using the Luminata™ Forte Western HRP Substrate (Millipore, Billerica, MA, USA). The protein band was detected using an ImageQuant 400™ Imager (GE Healthcare Life Sciences, Chicago, Illinois, USA). A duplicate Coomassie brilliant blue (CBB)-stained gel (Bio-Rad Laboratories, Hercules, CA, USA) was used as a loading control.

1.5 Enzyme-linked immunosorbent assay (ELISA)

ELISA running protocols were as follows:

	ApoB-100	FINC	C4A	C4B
Primary antibody	Monoclonal capture antibody (Capture mAb)	Rabbit anti-human FN1 pAb	Pre-coated ELISA plate containing anti-C4a Ab	Pre-coated ELISA plate containing anti-C4b Ab
Dilution	1:500	1:100	-	-
Incubation	Overnight, 4–8 °C	120 min at 37 °C	90 min at 37 °C	120 min at 37 °C
Secondary antibody	100 μL Detection mAb (biotin-conjugated)	100 μL Biotinylated Goat anti-human FN1 pAb	100 μL Biotinylated Detection Ab	100 μL Biotin-conjugated anti-C4b Ab
Dilution	1:1000	1:100	1:100	1:100
Incubation	60 min at room temperature	60 min at 37 °C	60 min at 37 °C	60 min at 37 °C
Conjugate	Streptavidin-ALP	Avidin-Biotin-Peroxidase Complex	Avidin-HRP Conjugate	Avidin-HRP Conjugate
Dilution	1:1000	1:100	1:100	1:100

Incubation	60 min at room temperature	30 min at 37 °C	30 min at 37 °C	60 min at 37 °C
Substrate	pNPP Substrate	TMB Substrate	TMB Substrate	TMB Substrate
Incubation	60 min at room temperature	20 min at 37 °C	15 min at 37 °C	15 min at 37 °C

Ab, antibody; mAb, monoclonal antibody; pAb, polyclonal antibody; HRP, horseradish peroxidase; pNPP, p-nitrophenylphosphate.

1.6 Thiobarbituric acid (TBA)-reactive substance (TBARS) assay

Ten microliters of plasma sample were mixed with 100 μL of a TBARS solution (15% (w/v) trichloroacetic acid, 0.38% (w/v) TBA, and 0.25 N HCl) and heated to 100 °C for 30 min. Then, the mixture was cooled in an ice bath for 2 min and subsequently centrifuged at 3000 rpm for 2 min. After centrifugation, 80 μL of the supernatant was transferred to a 96-well microtiter plate. The absorbance was detected at 535 nm. MDA levels of plasma were determined in accordance with the difference in absorbance between the test and standard samples, with MDA serving as a standard. Results are expressed in μmol/L.

1.7 Measurement of MDA-modified protein adducts

Fresh MDA preparation followed the Senthilkumar protocol.[5] Eighty-eight microliters of MDA bis(dimethyl acetal) was treated with 12 μL of 4 N HCl and 400 μL of Milli-Q® water at 37 °C for 10 min. The reaction was stopped by adjusting the pH to 7.4 with the addition of 1 N NaOH, and the volume was adjusted to 1 mL by adding Milli-Q® water to prepare a 0.5 M fresh MDA solution. One milligram of the MDA-modified BSA (MDA-BSA) adduct standards was prepared by adding a 150 mM MDA solution and dried fatty acid-free bovine serum albumin (BSA; Sigma-Aldrich, St. Louis, MO, USA) for 16 h at 4 °C in the dark. One hundred microliters of

plasma samples (10 μg/mL) or MDA-BSA adduct standards (0~10 μg/mL) were loaded on a 96-well microtiter plate and incubated overnight at 4 °C. Afterwards, the plates were washed with phosphate-buffered saline (PBS) with 0.05% Tween-20 (PBST), and they were blocked with PBS containing 3% BSA (Sigma-Aldrich) at 37 °C for 1 h. After washing the plates with PBST, we added a rabbit polyclonal anti-MDA antibody (dilution 1:1000, Cell Biolabs) and incubated samples and standards at room temperature (RT) for 5 h. Next, after washing the plates, we added a horseradish peroxidase (HRP)-conjugated goat anti-rabbit IgG antibody (sc-2054; dilution 1:1000; Santa Cruz Biotechnology, Dallas, TX, USA) as the secondary antibody at RT for 1 h and measured the bound antibody-HRP using the SureBlue Reserve™ TMB Microwell Peroxidase Substrate (Kirkegaard & Perry Laboratories, Gaithersburg, MD, USA) after incubation for 40 min at RT. The color reaction was stopped using 1 N HCl, and the absorbance was measured at 450 and 620 nm. Levels of MDA-protein adducts were detected using a standard MDA-BSA adduct curve.

1.8 Detection of autoantibody isotypes against BSA, MDA-BSA adduct, unmodified and MDA-modified peptides

First, 100 μL of 0.5 M MDA was mixed with 1 mg of peptide and incubated at 37 °C for 3 h. Dialysis was performed against PBS containing 1 mM EDTA at pH 7.4. The solution was incubated at 4 °C for 18 h (overnight). A solution of 10 μg of BSA, MDA-BSA adduct, unmodified or MDA-peptides in PBS was loaded onto a 96-well microplate and incubated overnight at 4 °C. After washing and blocking the plates at 37 °C for 1 h, 100-fold-diluted plasma was added and incubated for 2 h. After washing the plates, rabbit anti-human IgG-HRP (dilution 1: 30,000, Santa Cruz Biotechnology) and rabbit anti-human IgM-HRP (dilution 1:10,000, Santa Cruz

Biotechnology) antibodies were used to detect the deposition of autoantibodies against unmodified peptides or MDA-modified peptide adducts. The plates were incubated at 37°C for 1 h. The bound antibody-HRP was measured using the SureBlue Reserve™ TMB Microwell Peroxidase Substrate (Kirkegaard & Perry Laboratories) and incubated for 30 min at RT; the color reaction was stopped using 1 N HCl. The optical density (OD) was measured at wavelengths of 450 and 620 nm. OD values were used to determine the concentration in μg/mL based on a standard curve generated for each plate.

1.9 Feature selection and ranking

Feature selection was conducted using a consensus procedure across four tree-based algorithms (LightGBM, CatBoost, XGBoost, and RF) to identify features differentiating T2DM/CAD ≥70% stenosis from the combined group of T2DM/CAD <70% stenosis and T2DM.[6] Within the Shap-Cov framework, we outline a conceptual Python workflow using SHAP with coefficient of variation (SHAP-Cov), integrating explainable machine learning with statistical uncertainty quantification.[7] The core principle utilizes SHAP values to quantify the marginal contribution of each variable to the model's predictions.[8] To verify the reliability of these attributions, a Bootstrap-based procedure (100 iterations) was used to calculate the SHAP-Cov (95% confidence interval (CI)), AUC (95% CI), and Brier score (95% CI) for each candidate feature. The statistical significance test required that only features with a SHAP-Cov (95% CI) showing significant (not overlapping zero) and consistent effect directions across the first (Q1) and fourth (Q4) quartiles be retained. Following stability filtering, the selection was optimized to achieve the maximum AUC (95% CI) for discrimination, while maintaining a Brier score (95% CI) < 0.2 to ensure precise probability calibration.[9] Finally, multicollinearity was addressed by ensuring a

variance inflation factor (VIF) < 5.0.[10] This multi-step selection process identifies predictors that balance statistical rigor with clinical interpretability. Features were ranked by their mean |SHAP value|, integrating criteria such as SHAP-Cov, AUC-based discrimination, Brier score calibration, and VIF constraints.

1.10 Model construction and interpretation

We applied five algorithms, including RF, XGBoost, LightGBM, CatBoost, and LR, to our small-scale cohort, comparing T2DM vs. T2DM/CAD <70% stenosis, T2DM vs. T2DM/CAD ≥70% stenosis, combined T2DM and T2DM/CAD <70% stenosis vs. T2DM/CAD ≥70% stenosis, and T2DM/CAD <70% stenosis vs. T2DM/CAD ≥70% stenosis. Data preprocessing involved median imputation for missing values and feature standardization to ensure numerical stability. To strictly prevent information leakage and ensure unbiased performance evaluation, we implemented a nested CV design. The outer loop comprised 5-fold repeated stratified CV to preserve class balance, and performance uncertainty was quantified using 2,000 bootstrap resamples. Within each outer training fold, an inner 3-fold stratified CV loop was used exclusively for feature selection and hyperparameter tuning. To ensure robustness and clinical interpretability, feature selection was embedded within a nested CV framework, with all preprocessing confined to the inner training partitions to prevent leakage. The workflow followed a hierarchical, stability-oriented approach. First, correlation pre-filtering was performed using Spearman's ρ, where one of each highly collinear pair ($\rho > 0.60$) was discarded, while clinically essential markers (e.g., IgG anti-APOB-100$^{1662-1683}$ MDA) were preserved. Second, multicollinearity elimination was conducted by iteratively calculating Variance Inflation Factors (VIF)

and removing variables with VIF>10 to mitigate multi-way dependencies. Third, SHAP stability filtering was applied within each training fold, with SHAP values computed using 500 bootstrap resamples; predictors with unstable attributions (SHAP-CoV >1.5) were excluded to ensure reproducible interpretability.[8] Finally, Sequential Forward Selection (SFS) was deployed under the constraint of the Events per Variable rule (EPV ≥ 15), yielding parsimonious subsets (typically 2-3 predictors) tailored to each learner.[11] This multi-stage pipeline reduced redundancy, controlled variance inflation, and ensured that retained predictors were both statistically stable and clinically meaningful.

Discriminative performance was quantified using Mean AUC, Pooled AUC, sensitivity, specificity, and F1-score with 95% CIs. Model calibration was optimized via Platt scaling (sigmoid calibration) for small-sample validation and was evaluated using Brier scores and calibration slopes (95% CI). Clinical utility was determined by DCA, specifically evaluating net benefit at a 20% threshold probability. Model interpretability was established through SHAP analysis. Global insights were derived from SHAP bar plots (ranking predictors by mean |SHAP value|) and SHAP summary plots (visualizing impact direction via dots distribution), supplemented by SHAP dependence plots for feature effect patterns and potential interactions. Local interpretability was assessed using SHAP waterfall plots and force plots for selected case indices to visualize individualized prediction contributions.

1.11 Hyperparameters for small-sample machine learning models: Random Forest (RF), eXtreme Gradient Boosting (XGBoost), Light Gradient Boosting Machine (LightGBM) Categorical Boosting (CatBoost), and Logistic Regression (LR)

To classify comorbid type 2 diabetes mellitus and coronary artery disease (T2DM/CAD) within our clinical cohort (N = 165), five machine learning paradigms

were implemented: RF, XGBoost, LightGBM, CatBoost, and LR. Given the constrained sample size and complex pathophysiological interactions, default hyperparameters risk variance inflation and empirical overfitting[12]. Accordingly, we constructed a conservative and regularized hyperparameter search space to prioritize simplicity and generalizability. Model selection and evaluation were embedded within a nested CV framework. The outer loop employed repeated stratified k-fold CV ($k = 5$, 5 repeats; 25 iterations), preserving class distributions and mitigating data-splitting bias[13]. The inner loop executed a systematic grid search combined with stratified k-fold CV ($k = 3$), ensuring unbiased hyperparameter optimization while maintaining balanced class representation across folds. Reproducibility was ensured by setting the random seed to 42. For ensemble models, structural complexity was constrained by limiting maximum tree depth to 2-3, estimator counts capped (30-50 for RF/XGBoost/LightGBM; 150-200 for CatBoost), and node-splitting thresholds elevated (RF leaf 4, split 10, using localized feature subset selection; XGBoost min_child_weight 3-5; LightGBM min_child_samples 4-20). Gradient boosting frameworks adopted low learning rates (0.02-0.1) and strong $L2$ penalties (reg_lambda or l2_leaf_reg up to 50-60), shrinking leaf weights toward the global mean[14]. LR was restricted to pure $L2$ regularization (ridge), with inverse strength C spanning 0.01-2.0 to mitigate multicollinearity[15]. Optimization employed the saga solver with extended iterations (max_iter 5000) to ensure convergence stability under data perturbations. Table S2 summarizes the hyperparameter search spaces for all models, reflecting the conservative boundaries adopted to maintain model stability under small-scale cohorts.

1.12 Fairness analysis using a calibrated Categorical Boosting

(CatBoost) and Logistic Regression (LR)

To ensure a robust and unbiased fairness evaluation, a calibrated CatBoost model was benchmarked against an uncalibrated LR baseline using the same validation framework. Model evaluation was conducted within a nested CV. The outer loop used 5-fold repeated stratified CV with 5 repetitions (25 folds, N=165), and inner loop performed a systematic grid search integrated with a stratified 3-fold CV. For CatBoost, structural complexity was constrained by limiting maximum tree depth to 2-3, boosting iterations capped at 150-200, and learning rates restricted to 0.02-0.1. Leaf-weight L2 regularization was restricted to the range 15.0-50.0 to impose strong shrinkage and stabilize model complexity, as detailed in Table S2. Post-hoc miscalibration was corrected through Platt scaling implemented within a nested 3-fold CalibratedClassifierCV framework.[16] LR maintained parity through an identical 3-fold grid search, confined to an $L2$ penalty range of 0.01-2.0, solved with the SAGA algorithm (max_iter 5000), without additional calibration. OOF prediction probabilities were aggregated into a cohort-level latent risk profile comprising 165 subjects. Predictive performance was assessed using pooled AUC, calibration measured by Brier score,[17] and clinical net utility evaluated through decision curve analysis (DCA) at a pre-specified threshold of 20%.[18] NB at 20% was explicitly calculated to reflect the clinical utility of the models under a conservative, clinically actionable intervention threshold. Empirical uncertainty was estimated using non-parametric bootstrapping with 1,000 resamples, driving 95% CIs.[19] Algorithmic fairness was audited by stratifying the cohort into sex (Male vs. Female) and age (≥65 vs. <65 years). Subgroup differences in pooled AUROC, Brier score, and net benefit at the 20% threshold were quantified using independent two-sample tests and 1,000-iteration bootstrap permutation analyses, under established fairness evaluation

protocols. Between-group p-values were formally reported to identify systematic bias or disparities in clinical utility, supporting ethical eligibility for deployment.[20]

Reference:

(1) Faul, F.; Erdfelder, E.; Buchner, A.; Lang, A. G. Statistical power analyses using G*Power 3.1: tests for correlation and regression analyses. *Behav. Res. Methods* **2009**, *41* (4), 1149-1160.

(2) Poltep, K.; Tesena, P.; Yingchutrakul, Y.; Taylor, J.; Wongtawan, T. Optimisation of a serum albumin removal protocol for use in a proteomic study to identify the protein biomarkers for silent gastric ulceration in horses. *J. Equine Sci.* **2018**, *29* (3), 53-60.

(3) Glatter, T.; Ludwig, C.; Ahrne, E.; Aebersold, R.; Heck, A. J.; Schmidt, A. Large-scale quantitative assessment of different in-solution protein digestion protocols reveals superior cleavage efficiency of tandem Lys-C/trypsin proteolysis over trypsin digestion. *J. Proteome Res.* **2012**, *11* (11), 5145-5156.

(4) Cannon-Carlson, S.; Tang, J. Modification of the Laemmli sodium dodecyl sulfate-polyacrylamide gel electrophoresis procedure to eliminate artifacts on reducing and nonreducing gels. *Anal. Biochem.* **1997**, *246* (1), 146-148.

(5) Senthilkumar, M.; Amaresan, N.; Sankaranarayanan, A. (2021). Estimation of Malondialdehyde (MDA) by Thiobarbituric Acid (TBA) Assay. In: Plant-Microbe Interactions. Springer Protocols Handbooks. Humana, New York, NY.

(6) Bentéjac, C.; Csörgő, A.; Martínez-Muñoz, G. A comparative analysis of gradient boosting decision tree algorithms. *Artificial Intelligence Review* **2021**, *54* (3), 1937-1967.

(7) Brooks, L.; Harun, R.; Jin, J. Y.; Lu, J. Shap-Cov: An Explainable Machine Learning Based Workflow for Rapid Covariate Identification in Population Modeling. *CPT Pharmacometrics Syst. Pharmacol.* **2025**, *14* (8), 1322-1331.

(8) Lundberg, S. M.; Lee, S.-I. A Unified Approach to Interpreting Model Predictions. *Advances in Neural Information Processing Systems 30 (NIPS 2017)*.

(9) Steyerberg, E. W.; Vickers, A. J.; Cook, N. R.; Gerds, T.; Gonen, M.; Obuchowski, N.; Pencina, M. J.; Kattan, M. W. Assessing the performance of prediction models: a framework for traditional and novel measures. *Epidemiology* **2010**, *21* (1), 128-138. (10) Dormann, C. F.; Elith, J., Bacher, S.; Buchmann, C.; Carl, G.; Carré, G.; Marquéz, J. R. G.; Gruber, B.; Lafourcade, B.; Leitão, P. J.; Münkemüller, T.; McClean, C.; Osborne, P. E.; Reineking, B.; Schröder, B.; Skidmore, A. K.; Zurell, D.; Lautenbach, S. Collinearity: A review of methods to deal with it and a simulation study evaluating their performance. *Ecography* **2013**, *36* (1), 27-46.

(11) Peduzzi, P.; Concato, J.; Kemper, E.; Holford, T. R.; Feinstein, A. R. A simulation study of the number of events per variable in logistic regression analysis. *J. Clin. Epidemiol.* **1996**, *49* (12), 1373-1379.

(12) Hawkins, D. M. The problem of overfitting. *J. Chem. Inf. Comput. Sci.* **2004**, *44* (1), 1-12.
(13) Varma S, S. R. Bias in error estimation when using cross-validation for model selection. *BMC Bioinformatics* **2006**, *7*, 91.
(14) Friedman, J. H. Greedy function approximation: A gradient boosting machine. *Annals of Statistics* **2001**, *29* (5), 1189-1232.
(15) Hui Zou, T. H. Regularization and Variable Selection Via the Elastic Net. *Journal of the Royal Statistical Society Series B: Statistical Methodology* **2005**, *67* (2), 301-320.
(16) Platt, J. Probabilistic Outputs for Support Vector Machines and Comparisons to Regularized Likelihood Methods. *Advances in Large Margin Classifiers* **1999**, *10* (3), 61-74.
(17) Brier, G. W. Verification of forecasts expressed in terms of probability. *Monthly Weather Review* **1950**, *78* (1), 1-3.
(18) Vickers, A. J.; Elkin, E. B. Decision curve analysis: a novel method for evaluating prediction models. *Med. Decis. Making* **2006**, *26* (6), 565-574.
(19) Simkus, A.; Coolen, F. P. A.; Coolen-Maturi, T.; Karp, N. A.; Bendtsen, C. Evaluation of bootstrap methods for coverage estimation and prediction accuracy with small samples. *Communications in Statistics - Simulation and Computation* **2026**, 1-7.
(20) Chen, R. J.; Wang, J. J.; Williamson, D. F. K.; Chen, T. Y.; Lipkova, J.; Lu, M. Y.; Sahai, S.; Mahmood, F. Algorithmic fairness in artificial intelligence for medicine and healthcare. *Nature Biomedical Engineering* **2023**, *7* (6), 719–742.
